# Comparative analysis of tandem repeats from hundreds of species reveals unique insights into centromere evolution

**DOI:** 10.1186/gb-2013-14-1-r10

**Published:** 2013-01-30

**Authors:** Daniël P Melters, Keith R Bradnam, Hugh A Young, Natalie Telis, Michael R May, J Graham Ruby, Robert Sebra, Paul Peluso, John Eid, David Rank, José Fernando Garcia, Joseph L DeRisi, Timothy Smith, Christian Tobias, Jeffrey Ross-Ibarra, Ian Korf, Simon WL Chan

**Affiliations:** 1 Department of Molecular and Cell Biology and Genome Center, University of California, Davis, 1 Shields Ave, Davis, CA 95616, USA; 2 Department of Plant Biology, University of California, Davis, 1 Shields Ave, Davis, CA 95616, USA; 3 USDA-ARS, Western Regional Research Center, 800 Buchanan St, Albany, CA 94710, USA; 4 Department of Evolution and Ecology, University of California, Davis, 1 Shields Ave, Davis, CA 95616, USA; 5 Department of Biochemistry and Biophysics, University of California, San Francisco, 1700 4th St, San Francisco, CA 94158, USA; 6 Pacific Biosciences, 1380 Willow Rd, Menlo Park, CA 94025, USA; 7 Department of Animal Production and Health, Universidade Estadual Paulista, IAEA Collaborating Centre in Animal Genomics and Bioinformatics, Rua Clóvis Pestana, 793-16050-680, Aracatuba, SP, Brazil; 8 Howard Hughes Medical Institute, 4000 Jones Bridge Rd, Chevy Chase, MD 20815, USA; 9 USDA-ARS, US Meat Animal Research Center, State Spur 18D, Clay Center, NE 68933, USA; 10 Department of Plant Sciences, Center for Population Biology, and Genome Center, University of California, Davis, 1 Shields Ave, Davis, CA 95616, USA

## Abstract

**Background:**

Centromeres are essential for chromosome segregation, yet their DNA sequences evolve rapidly. In most animals and plants that have been studied, centromeres contain megabase-scale arrays of tandem repeats. Despite their importance, very little is known about the degree to which centromere tandem repeats share common properties between different species across different phyla. We used bioinformatic methods to identify high-copy tandem repeats from 282 species using publicly available genomic sequence and our own data.

**Results:**

Our methods are compatible with all current sequencing technologies. Long Pacific Biosciences sequence reads allowed us to find tandem repeat monomers up to 1,419 bp. We assumed that the most abundant tandem repeat is the centromere DNA, which was true for most species whose centromeres have been previously characterized, suggesting this is a general property of genomes. High-copy centromere tandem repeats were found in almost all animal and plant genomes, but repeat monomers were highly variable in sequence composition and length. Furthermore, phylogenetic analysis of sequence homology showed little evidence of sequence conservation beyond approximately 50 million years of divergence. We find that despite an overall lack of sequence conservation, centromere tandem repeats from diverse species showed similar modes of evolution.

**Conclusions:**

While centromere position in most eukaryotes is epigenetically determined, our results indicate that tandem repeats are highly prevalent at centromeres of both animal and plant genomes. This suggests a functional role for such repeats, perhaps in promoting concerted evolution of centromere DNA across chromosomes.

## Background

Faithful chromosomal segregation in mitosis and meiosis requires that chromosomes attach to spindle microtubules in a regulated manner via the kinetochore protein complex. As the site of kinetochore assembly, the centromere is the genetic locus that facilitates accurate inheritance. Deletion of the centromere or mutation of critical kinetochore proteins results in chromosome loss [1,2]. Proteins and DNA sequences involved in most essential cellular functions are characterized by their high degree of conservation. Given their conserved function, the observed rapid evolution of kinetochore proteins [3] and lack of homology of centromere repeats thus poses somewhat of a paradox [4].

Centromeres differ greatly in their sequence organization among species. In the budding yeast *Saccharomyces cerevisiae *a 125-bp sequence is sufficient to confer centromere function, and essential kinetochore proteins bind to this 'point centromere' in a sequence-dependent manner [5]. Point centromeres are a derived evolutionary characteristic, as ascomycete fungi more distantly related to *S. cerevisiae *have much longer centromere DNAs and do not rely on specific sequences to recruit kinetochore proteins [5,6]. In the limited set of plant and animal species that have been previously analyzed, centromere DNAs consist of megabase-sized arrays of simple tandem repeats (or satellite DNA), sometimes interspersed with long terminal repeat transposons [7-9]. Some taxa exhibit higher order repeat (HOR) structures, in which multiple polymorphic monomers make up a larger repeating unit [10,11]. When centromeric tandem repeat sequences of different species are compared, sequence similarity appears limited to short evolutionary distances [4,5]. In fact, specific DNA sequences are probably dispensable for centromere function in most eukaryotes, as kinetochore proteins in diverse organisms can assemble on non-centromeric sequences [2,12-16]. In humans, these 'neocentromeres' have been found through karyotype analysis and can arise at many different loci [17]. In some animals and plants, individual chromosomes - or even the entire chromosome complement - may lack high-copy tandem repeat arrays [2,13,15,16] and in rare cases centromere repeat sequences differ between chromosomes [18,19] The epigenetic nature of centromere location may be explained by the fact that kinetochores assemble on nucleosomes containing a centromere-specific histone H3 variant, CENH3 (CENP-A in human). Extreme cases of kinetochore protein assembly on diverse sequences are seen in polycentric [18] and holocentric chromosomes [20]. The former has a single very large primary constriction that contains three-to-five CENH3 foci [18], whereas the latter has CENH3-bound sequences and microtubule attachment sites along the entire length of mitotic chromosomes [21]. Despite their dispensable nature, the presence of tandem repeats at the centromere locus of most animals and plants suggests that they serve a function.

Many questions about centromere repeat evolution remain unanswered. How prevalent are high-copy tandem repeat arrays at the centromeres of different animal and plant species? Studies of centromere DNA in animals and plants have so far focused on single organisms or on small clades [5,22] and few review articles have been dedicated to a broad survey of tandem repeats [23]. No conserved motif has been found for centromere DNA except in small clades (for example, the CENP-B box found in mammalian centromeres [24]). Are there shared properties among centromeric tandem repeats from diverse animals and plants? In *Saccharomyces cerevisiae *and closely related yeast species, short centromere DNA sequences evolve three times faster than other intergenic regions of its genome [25,26]. How rapidly do centromere tandem repeats evolve and which molecular processes govern their evolution? We performed a survey of tandem repeats in a large and phylogenetically diverse set of animal and plant species in order to address these questions.

Conventional methods used to identify centromeric tandem repeats, particularly CENH3 chromatin immunoprecipitation, are labor intensive and thus difficult to do on a large scale. In this paper, we identified and quantified the most abundant tandem repeats from 282 animal and plant species using a newly developed bioinformatic pipeline. Our method can utilize shotgun whole genome shotgun (WGS) sequence data from various sequencing platforms with varying read lengths, including Sanger, Illumina, 454, and Pacific Biosciences (PacBio). Candidate centromere repeat sequences were characterized by a seemingly unbiased nature. Repeat monomers varied widely in length, GC composition and genomic abundance. Despite great differences in sequence composition, centromere DNAs appeared to evolve by expansion and shrinkage of arrays of related repeat variants (the 'library' hypothesis [27]). Using PacBio single molecule real-time sequencing to span many contiguous monomers, we characterized the mixing of repeat variants within a single array and the presence of higher-order repeating units. Our data greatly broaden the phylogenetic sampling of centromere DNA, putting evolutionary conclusions about this fast evolving chromosome region on a firmer footing.

## Results

### A bioinformatic pipeline to identify candidate centromere tandem repeats

**Figure 1:**
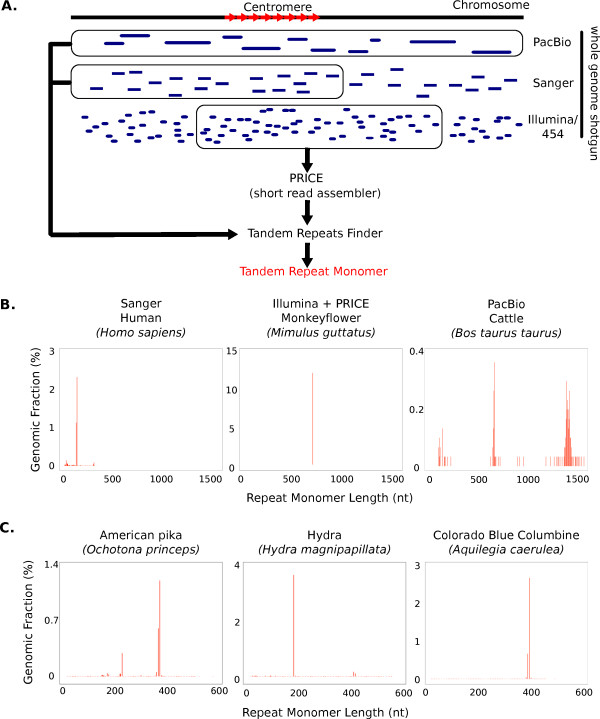
**A bioinformatic pipeline to identify candidate centromere DNAs based on their tandem repeat nature and abundance**. **(a) **Random shotgun sequences from a variety of platforms can be used to identify the most common tandem repeat monomer. Sanger and PacBio reads are usually long enough to contain multiple copies of a tandem repeat. Illumina and 454 reads are generally too short, and must be assembled to create longer sequences. Tandem repeat monomers were identified by Tandem Repeats Finder (TRF). **(b) **Identification of known centromere tandem repeats from three species. The human centromere repeat is 171 bp in length. The 728-bp monkeyflower centromere repeat is too long to be found in Sanger reads, but a PRICE assembly of Illumina reads reveals the known repeat. The 1,419-bp cattle centromere repeat and a less abundant 680-bp tandem repeat were directly identified from PacBio reads. Note that the graph for monkeyflower has no background of low abundance tandem repeats because these were not assembled by PRICE. **(c) **Three examples of *de novo *identification of centromere tandem repeats. Sanger WGS reads from the American pika, Hydra, and Colorado Blue Columbine revealed 253-bp, 183-bp, and 329-bp repeat monomers, respectively. nt, nucleotides.

Centromere DNAs in most animal and plant species share two distinctive properties: the presence of tandem repeats, and their extremely high repeat abundance (often >10,000 copies per chromosome). Therefore, we hypothesized that the most abundant tandem repeat in a given genome would be the prime candidate for the centromere repeat (our method is not designed to find centromere-specific retrotransposons or chromosome-specific repeat sequences). To find such sequences *de novo *from WGS sequence data, we developed a bioinformatic pipeline that identifies tandem repeats from a variety of sequencing technologies with different read lengths (see Materials and methods; Figure 1a). For example, the 171-bp human centromere repeats [11] were identified from Sanger reads and the approximately 1,400-bp Bovidae repeats [28-30] were identified from PacBio reads (Figure S5 in Additional file 2). In both cases tandem repeats were directly identified from WGS reads (Figure 1a). As few as 1,000 Sanger reads were needed to identify the human repeat monomer, confirming that highly abundant tandem repeats can be found from a very small amount of shotgun sequence data. Tandem Repeats Finder (TRF) requires the presence of at least two tandem copies within a read to find a tandem repeat. The 728-bp monkeyflower (*Mimulus guttatus*) repeats [31] were identified from Illumina reads, which were assembled with the short-read assembler PRICE (Paired-Read Iterative Contig Extension; Figure 1a). The assembly steps allowed for identification of candidate centromere repeats that were too long to be identified directly from Sanger reads, with the caveat that these newly assembled repeats are consensus sequences (see Materials and methods). Identifying candidate centromere tandem repeat from ribosomal repeats, zinc-finger domain repeats, retrotransposons, and knob repeats was done by all-versus-all BLAST search combined with nucleotide BLAST (blastn function in [32]) search. In each case, the most abundant tandem repeat unit was considered to be the candidate centromere DNA.

### Validating the bioinformatic pipeline by identifying known centromere tandem repeats

To validate candidate centromere tandem repeats, we compared our results to sequences described in the literature (Table S2 in Additional file 3). Centromere DNAs have been characterized by restriction enzyme-based methods (for example, laddering on ethidium bromide-stained gels) combined with fluorescence *in situ *hybridization (FISH), and by chromatin immunoprecipitation (ChIP) with antibodies raised against a kinetochore protein (typically the centromere-specific histone CENH3). Overall, centromere DNA sequences have been described from 43 of the 282 species in this study. In 38 out of 43 cases, we identified a similar repeat to that reported in the literature (Table S2 in Additional file 3). In the case of opossum (*Monodelphis domestica*) and elephant (*Loxodonta africana*), centromere repeat monomers are believed to be very long (528 and 936 bp, respectively) [33] and therefore cannot be found using Sanger reads. We lacked suitable Illumina or 454 data to allow assembly of long tandem repeats from these species, and did not have PacBio data to find long repeats directly. Potato and pea are unusual in that centromere repeats differ across chromosomes [18,19], with some potato chromosomes lacking tandem centromere repeats entirely [19]. These repeats are too diverse and too long to be identified by our pipeline (upper limit of 2 kbp or half the length of a WGS read). Other discrepancies between our candidate centromere repeats and published sequences may be explained by the fact that many previous studies used experimental methods that did not quantify all tandem repeats in the genome (see Table S2 in Additional file 3 for a per species explanation).

In limited cases, an assembled reference genome can assist in identifying a *bona fide *centromere tandem repeat. As expected for a true centromere DNA sequence, the 1,419 bp repeat from cattle is generally clustered into one large array on all 30 chromosomes in the UMD3.0 genome assembly [34]. These putative centromere arrays contain hundreds of repeat copies (notably, secondary arrays elsewhere in this genome assembly contain only five to ten copies of the monomer).

CENH3 ChIP followed by sequencing is the most definitive method to confirm that a given sequence underlies the functional kinetochore. Only 13 species out of the 43 had CENH3 ChIP-seq data, and our method correctly identified the published centromere tandem repeat in 10 out of 13 of these cases. The three exceptions were opossum, elephant, and potato, where we lacked appropriate sequencing reads to find long tandem repeats (opossum and elephant) or the tandem repeats were too diverse (potato). In summary, our bioinformatic pipeline identified the correct centromere tandem repeat in the large majority of cases where experimental data were available.

In two cases, the most abundant tandem repeat was not the known centromere DNA sequence. In the sequenced maize strain B73 (*Zea mays*) [35], heterochromatic 'knobs' contain highly abundant tandem repeats that outnumber the centromere tandem repeat CentC [36]. Knob number, size, repeat abundance and distribution can differ depending on the particular maize variety analyzed, as repeat abundance is variable between isolates [37,38]. A 178-bp tandem repeat is present at the centromere of the Tammar wallaby (*Macropus eugenii*), but this sequence was only the third most abundant tandem repeat in our analysis [38]. By mammalian standards, Tammar wallaby centromeres are unusually small (approximately 450 kbp per chromosome), and tandem repeats make up a minority of this chromosome region because it is also populated by a centromere-specific retroelement [39].

### Candidate centromere tandem repeats from many uncharacterized animal and plant species

**Figure 2:**
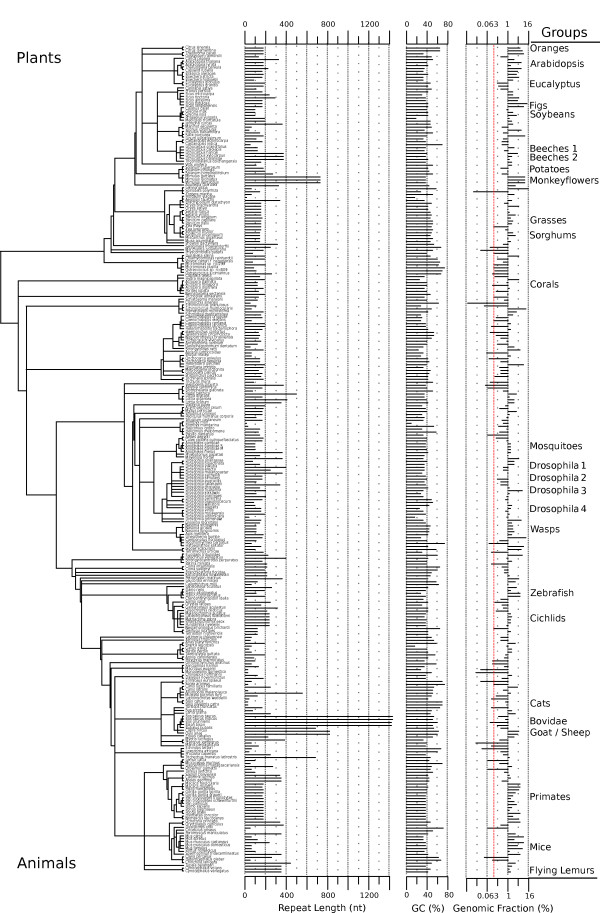
**Centromere tandem repeat details from diverse animal and plant genomes**. The phylogenetic relationships between 282 species (204 Animalia and 78 Plantae) are shown. For each species, the figure shows tandem repeat length, GC content, and genomic fraction (log 2 scale) for the (candidate) centromere repeat monomer. Taxonomic relationships were derived from the NCBI taxonomy website. Approximately one-third of the species (84 out of 282) could be clustered into 26 groups (light red horizontal bars) that exhibited sequence similarity of the tandem repeat monomer within each group. No sequence similarity was found outside these groups, or between them. The most distantly related species within a group diverged about 50 million years ago.

To detect candidate centromere repeats, we analyzed a total of 282 species, comprising 78 plants and 204 animals spanning 16 phyla (Figure 2; Table S1 in Additional file 3). Sanger, Illumina, and 454 sequences were obtained from public databases, and we also performed our own PacBio sequencing. The WGS data included 171 species from Sanger sequencing, 132 from Illumina, 13 from 454, and 9 from PacBio. For the 37 species that had both Sanger and assembled Illumina data, both data types yielded the same candidate centromere repeat in the majority of cases (28 out of 37). In most cases where analysis of unassembled Sanger reads revealed a different repeat to Illumina data, individual Sanger reads were too short to find the long repeat monomers (see Table S4 in Additional file 3 for a per species explanation).

Many species whose centromere DNAs had not been previously characterized showed a single tandem repeat whose abundance was much greater than all other tandem repeats in the genome. For example, the American pika (*Ochotona princeps*), *Hydra *(*Hydra magnipapillata*), and Colorado Blue Columbine (*Aquilegia caerulea*) had candidate centromere DNAs of 341 bp, 183 bp, and 329 bp, respectively (Figure 1c).

The most accurate measurements of centromere tandem repeat array size in animals and plants are generally in the range of approximately 500 kbp to several Mbp [10,39-41]. Although estimated repeat abundance is subject to several experimental biases, we calculated the average amount of repeat per chromosome, and most organisms in our survey were estimated to contain hundreds of kilobase pairs (Table S1 in Additional file 3). Since our analysis was based on WGS data, it is not possible to detect chromosome-to-chromosome variation [2,15,16].

### How rapidly do centromere DNA sequences evolve?

**Figure 3:**
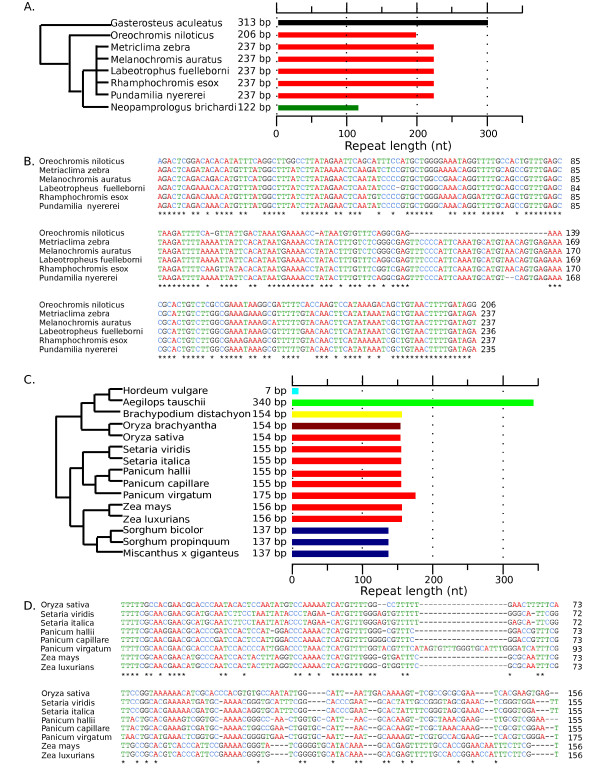
**Evolution by indel acquisition and coexistence of repeat variants support the 'library' hypothesis**. **(a) **Candidate centromere repeat sequences of eight cichlids were analyzed for interspecies sequence similarity. The Princess cichlid *Neolamprologus brichardi *lacked centromere repeat similarity with its sister clade of Lake Malawi cichlids (shown in orange, and also including Nile tilapia). **(b) **Sequence alignment of candidate centromere repeats shows that Nile tilapia (*Oreochromis niloticus*) has a deletion relative to other cichlid species. **(c) **Candidate centromere repeat sequences of 15 grass species were analyzed for interspecies sequence similarity. We found two groups of species with centromere repeat sequences that were similar. The closely related *Sorghum *and *Miscanthus *species have similar 137 bp repeats (blue bars). The clade shown by red bars contains *Oryza sativa *(rice), which is relatively distant from the other species that have similar centromere tandem repeats (red bars). Although the centromere repeats of *Oryza brachyantha *and *Brachypodium distachyon *have repeat monomer length similar to the orange-highlighted group, no sequence similarity was found between them. Interestingly, no sequence similarity was found between the closely related *Zea *species and *Sorghum *species or between *Oryza *species and *Brachypodium, Aegilops*, or *Hordeum*. **(d) **Sequence alignment of candidate centromere repeats from eight grass species. Switchgrass (*Panicum virgatum*) is distinguished by the presence of a short insertion relative to the other species.

An all-versus-all BLAST search of our consensus repeats revealed that sequence conservation was limited to only very closely related species. We found 26 groups of species that showed sequence similarity between centromere tandem repeats (Figure 2; Figure S1 in Additional file 2). Notable groupings of species with substantial sequence similarity included the primates (Figure S2 in Additional file 2), cichlids (Figure 3a,b) and grasses (Figure 3c,d).

The well-studied nature of human centromeres, and the availability of many closely related species, make primates an excellent clade to illustrate the evolution of centromere DNAs [11,42,43]. Candidate centromeric tandem repeats in primates showed similarity between monkeys and apes (Figure S2 in Additional file 2), but these candidate centromere DNAs were unrelated to those in more basal primates (tarsiers and prosimians). We inspected lower abundance tandem repeat sequences from the TRF output, and no tandem repeat in tarsiers or prosimians was found to have sequence similarity to the primate candidate centromeric tandem repeat. These results reinforce recent findings showing that the aye-aye (*Daubentonia madagascariensis*) has centromere repeats with no similarity to monkeys and apes [43].

Cichlid fish are another clade in which we identified both conservation and rapid divergence of centromere repeats. Lake Malawi cichlids and the Nile tilapia (*Oreochromis niloticus*) had candidate centromere DNAs that shared 78% sequence similarity, although tilapia diverged from other cichlids 45 million years ago (MYA). The Princess cichlid *Neolamprologus brichardi *(from Lake Tanganyika) had a candidate centromere repeat with no sequence similarity to either the Lake Malawi cichlids or Nile tilapia, though *Neolamprologus *diverged from Lake Malawi cichlids only 30 MYA. Similar patterns of both conservation and rapid change can be seen in the grasses (Figure 3c,d). A maize-like centromere repeat can be found in *Panicum, Setaria*, and even in a species as distant as rice (*Oryza*), which diverged from maize approximately 41 MYA. In contrast, sorghum-maize (9 MYA) and *Hordeum*-*Aegilops *(14 MYA) comparisons show little to no sequence similarity.

**Figure 4:**
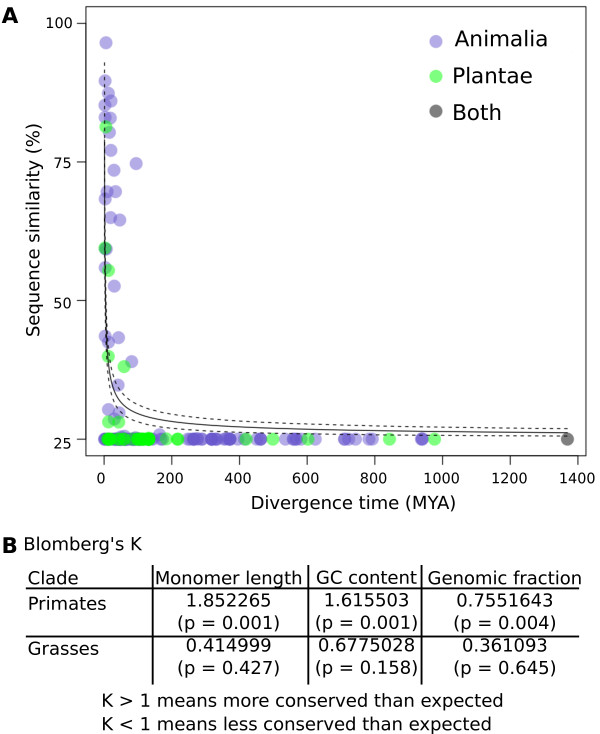
**Centromere tandem repeat monomers are conserved only between closely related species**. **(a) **Percentage identity between candidate centromere repeat sequences plotted against estimated divergence time. We averaged percentage identity between comparisons to generate a single value for each node in the phylogenetic tree (Figure 2). To accommodate unresolved relationships, we repeated the analysis on random resolutions of the tree. One such analysis is shown (quantitative results were very similar between analyses). **(b) **For primates and grasses, the phylogenetic signal was tested using Blomberg's *K *analysis for three different parameters: repeat monomer length, repeat monomer GC content and genomic abundance. In primates both repeat length and GC content were more conserved than expected (*K *> 1), whereas genomic abundance was less conserved than expected by a model of Brownian evolution (*K *< 1). Though *K *< 1 for all three traits in the grasses, none were significantly different from 1. *P*-values are shown in brackets.

To evaluate the rate of sequence evolution across the entirety of our sampled taxa, we assessed the conservation of sequence identity across the phylogeny using a node-averaged comparative analysis (Figure 4a). We fit a model of exponential decay with divergence, finding that, on average, sequence identity falls rapidly to background levels (that is, random 25% identity) after approximately 50 MYA.

### Candidate centromere tandem repeats from 282 animals and plants display no readily apparent conserved characteristics

If centromere DNAs are fast evolving, do their repeat monomers at least possess other conserved properties? As our survey is the broadest phylogenetic analysis of tandem repeats to date, we asked if candidate centromere DNAs from 282 species shared common characteristics. Our analyses showed that this was not the case.

**Figure 5:**
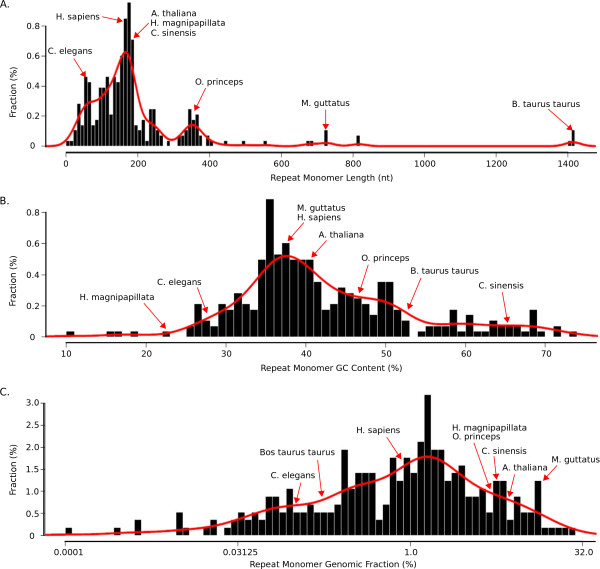
**Centromere tandem repeats lack conserved sequence properties**. **(a-c) **No strong bias was observed in distribution of centromere repeat monomer length (a), GC content (b), or genomic fraction (c).

First, centromere tandem repeat monomer length is not conserved. As CENH3 is essential for kinetochore nucleation, it has been hypothesized that centromere repeat monomers may tend to be about the size of one nucleosomal DNA [9,44], as is seen with human (171 bp), *Arabidopsis thaliana *(178 bp), and maize (156 bp) centromere DNAs. This is clearly not a universal rule, as some centromere tandem repeat monomers are much shorter and longer than nucleosomal sizes (for example, soybean at 92 bp [45,46] and cattle at 1,419 bp [30]) (Figure 5a). Plant species tended to have repeat sequences with lengths of approximately 180 bp, whereas we found a broader length distribution in animals. Modest trends in our data, however, may reflect sampling bias in the species for which WGS data were available in public archives rather than biologically meaningful preferences in centromere tandem repeat length.

Second, GC content of centromere tandem repeats is not conserved. Based on limited analysis of animal centromere repeats, it was suggested that centromeric DNA is AT-rich [4]. Our analysis of 282 species revealed that centromeric DNA can be very GC-rich (Figure 5b), although a slight preference for AT-rich tandem repeats was observed in animals. Plant species do not appear to have a preference for AT- or GC-rich centromere tandem repeats.

Third, the abundance of centromere tandem repeats varies widely (Figure 5c). We calculated repeat abundance by finding the proportion of reads that matched the repeat monomer (using a set of randomly sampled reads; Materials and methods; Additional file 1). Tandem repeat abundance can be compared between species, but is subject to variability introduced by different library construction protocols at particular sequencing centers, and by biases in the way different sequencing technologies capture high-copy repeats. We compared repeat abundance of 40 species for which there was sequence data from multiple sequencing technologies. On average, sequences derived from Illumina sequencing had higher estimated repeat abundances compared to Sanger, 454 or PacBio data. For most species we estimated that at least 0.5% of the genome was composed of the candidate centromeric tandem repeat, but the overall percentage was highly variable (Figures 2 and 5c).

Simple non-phylogenetic correlations found no relation between repeat length, GC content, and genomic fraction of candidate centromere tandem repeats (Figure S3 in Additional file 2). Similarly, we did not find a correlation between these factors and genome size, genome-wide GC content or chromosome number.

To explicitly test for conservation of sequence characteristics at a finer phylogenetic resolution, we searched for signals in phylogenetic trees that represented the grass and primate clades (Figure 4b). Both clades are of a similar age (40 to 45 MYA for the most divergent species) and show substantial sequence similarity among taxa. We calculated Blomberg's *K *statistic [47], a measure of phylogenetic conservation, for various tandem repeat characteristics. The *K *statistic indicates the amount of phylogenetic signal in the data. Values of *K *> 1 suggest that related taxa resemble each other more than would be expected given a null model in which the trait evolves along the tree according to Brownian motion. Values of *K *< 1 are observed when related taxa are less similar than expected under the null model. Although repeat monomer length, GC content, and genome fraction all had values of *K *< 1 in the grasses, none were statistically significant. In contrast, values for all three characteristics were significantly different from the null model in primates, with GC content and repeat monomer length showing *K *> 1 and repeat abundance *K *< 1. These data suggest that individual clades likely differ in terms of their tendency for closely related species to have centromere repeats that share conserved sequence characteristics.

### Which species lack candidate high-copy tandem repeats at their centromeres?

Which animal and plant genomes lack high-copy centromere tandem repeats? The nematode *Caenorhabditis elegans *is a useful negative control for measuring tandem repeat abundance (see red dashed line in the genomic fraction column of Figure 2), because it has holocentric chromosomes and has been reported to lack centromere tandem repeat arrays in its genome [21]. In total, 41 species had a lower abundance of tandem repeats than in *C. elegans*, and these could be assumed to lack high-copy centromere tandem repeats. Nine of these species are known to be holocentric [48] and are not expected to have large tandem arrays. Fungi such as *Saccharomyces cerevisiae, Candida albicans*, and *Schizosaccharomyces pombe *have small genomes and do not contain high-copy tandem repeat arrays at their centromeres [5]. Many of the other genomes that exhibited low tandem repeat abundance also had small genomes, including seven species of basal plants (green algae, moss, and liverwort) and 11 animals. A few species exhibited low tandem repeat abundance despite possessing large genomes (hedgehog (*Erinaceus europaeus*), tenrec (*Echinops telfairi*), seal (*Leptonychotes weddellii*) and dolphin (*Tursiops truncatus*). This may be due to these species having large repeat units that could not be identified in the available Sanger reads. While a definitive answer is not possible yet, it appears that species lacking large tandem arrays tend to have holocentric centromeres or small genomes.

### Higher order repeat structure and evolution of novel repeat monomers

**Figure 6:**
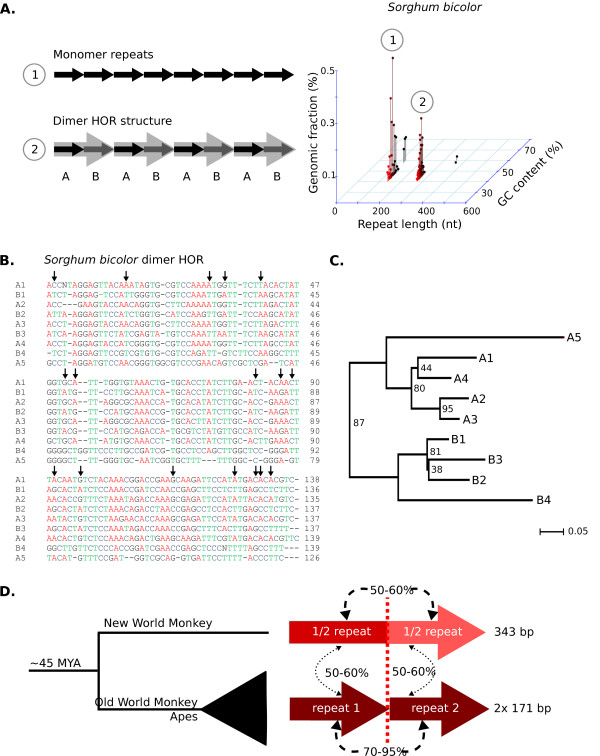
**Higher order repeat structures are prevalent in diverse animals and plants**. **(a) **Graphical representation of higher order repeat structure compared to simple monomer repeats. In the higher order repeat, two variants, A and B, form a single dimer repeat that is repeated in tandem. When plotting repeat monomer length by GC content by genomic fraction, two distinct peaks are seen for *Sorghum bicolor*. The second peak (2) is exactly double the length of the first peak (1). **(b) **Sequence alignment of repeat units from a single *Sorghum bicolor *Sanger read that exhibits a higher order repeat structure consisting of an AB dimer. The arrows point to SNPs unique for either the A or B repeat of the dimer. **(c) **Neighbor joining analysis showing grouping of A and B repeats from sequence alignment in B. Bootstrap numbers are shown. **(d) **Higher order repeat structures can lead to novel centromere repeats. In New World monkeys, the two halves of the 343-bp monomer are weakly related to each other and to the 171-bp repeat in Old World monkeys and apes.

Primate centromeres contain HOR structures [11,49], in which multiple repeat monomers with specific polymorphisms form a unit that itself is repeated (Figure 6a). HOR structure was easiest to observe in Sanger data, which combines relatively long reads with high sequence accuracy. We used the output from TRF [50] to identify higher order repeat structures among Sanger sequences from the NCBI trace archive. TRF reports both the repeat monomer, as well as repeating units carrying multimers of the monomer that may represent HOR structure. TRF-defined repeats that occupied approximately the same coordinates within a single read were compared to identify whether longer repeats were dimers of the basic monomer. In true HOR structures, the percentage identity between adjacent multimers should be much higher than between individual monomers (TRF should also report higher scores for the repeats with the longer monomer). Therefore, we filtered TRF output to detect these multimers that had both a higher percentage identity and a higher TRF score compared to the monomeric repeat that spanned the same coordinates.

Clear cases of HOR structure were identified in 76 of the 171 species with Sanger data. Phylogenetic trees constructed with individual monomers extracted from a single read showed that the 'A' monomers and 'B' monomers from a dimeric 'AB' structure that clustered separately (Figure 6b,c), confirming that the AB structure indeed represented a HOR unit. HOR structure has been previously described in primates, but our analysis shows that it is widespread across both plant and animal kingdoms. The capability to detect HOR units is limited by Sanger read length, so shorter repeat monomers were more likely to display HOR structures. We rarely identified HOR structures that had three or more copies of a repeat monomer, because such structures require at least six monomers to be found in a single Sanger read.

Can HOR structure result in evolution of a new centromere tandem repeat? The centromere repeat monomer has only been reported for one New World monkey and its length (343 bp) is essentially double the size of human alpha satellite [51]. We extended this analysis to three New World monkeys and fifteen Old World monkeys and apes (Figure 6d; Figure S2 in Additional file 2). All Old World monkeys and apes had a 171-bp candidate centromeric tandem repeat, whereas New World monkeys had a 343 bp candidate centromeric tandem repeat. If the 343 bp repeat is split into two equal halves and aligned to the 171 bp repeat, both halves align, but each has specific polymorphisms and indels (Figure 6d; Figure S1 in Additional file 2). These data suggest that in the New World monkey clade, a doubled version or dimer of the ancestral 171-bp repeat became the dominant centromeric tandem repeat. Such patterns of evolution are likely to be general, as they depend only on acquisition of polymorphism and a particular pattern of recombination within a repeat array [52].

Where HOR structure is present, it means that our calculated values for the abundance of candidate centromere repeats are most likely underestimates. A notable example of this occurs in the gorilla genome (*Gorilla gorilla gorilla*). We correctly identify the 171 bp centromere repeat as the most abundant repeat and this accounts for approximately 1.3% of the genome. However, we also identify a separate, but related, 340-bp repeat that represents a doubled version of the 171-bp repeat. This second repeat accounts for a further 1.2% of the genome, showing that dimeric HOR structure may be especially common among gorilla centromere repeats.

### Coexistence of related repeats support the 'library' hypothesis

The 'library' hypothesis aims to explain how centromere DNA evolves so rapidly [27]. This hypothesis assumes that variants of centromere tandem repeats co-exist within the same tandem arrays. Over time, the abundance of particular variants stochastically changes through both expansion and shrinkage [53,54], resulting in replacement of the most abundant variant with a different variant. Centromere repeat variants could arise by point mutation, deletion, insertion or by mixing of different parental sequences during allopolyploid formation (in all cases, a process such as gene conversion would be required to transfer variants between chromosomes) [55]. Are there cases in our data set that support the 'library' hypothesis? Specifically, do repeat variants differ and are there cases where such a repeat was able to colonize a genome and replace the original monomer?

Several Lake Malawi cichlids contained a 237-bp candidate centromeric tandem repeat, whereas the closely related Nile tilapia contained a shorter repeat of 206 bp (Figure 3a,b). However, the Nile tilapia did contain a less abundant, 237-bp repeat that was similar to the Lake Malawi cichlid repeat (Figure S4 in Additional file 2). This suggests that the centromere tandem repeat in the common ancestor of Lake Malawi cichlids and Nile tilapia was replaced by a related sequence (having either an insertion or deletion of 29 bp) in one of the two modern clades.

More support for the 'library' hypothesis was seen in the grasses (family *Poaceae*); this was the largest plant clade in our dataset that exhibited sequence similarity among most of its members. The modal length of repeat monomers in grasses was 156 bp, but deletions and insertions were found in several species (an 80 bp conserved motif between rice and maize was previously noted within this sequence [41]). Eight of the fifteen grass species had candidate centromere repeats that displayed no similarity to the common 156-bp sequence (Figure 3c,d). We then searched our data for less abundant tandem repeats related to the dominant repeat monomer. Sanger sequence data for four grass species revealed distinct centromere tandem repeat variants. Maize and foxtail millet (*Setaria italica*) only contained one variant each (variants A and D, respectively), witchgrass (*Panicum capillare*) had two variants (B and C) and the switchgrass genome contained three variants (A, B, and C). Variant B itself consists of two distinct repeats, one of 175 bp (variant B1) and another of 166 bp (variant B2). B2 differs from B1 by the deletion of 9 bp, but these two subvariants are otherwise very similar in sequence, so we consider them as one variant (variant B). The existence of related repeat variants in switchgrass and witchgrass is similar to our observations in Lake Malawi cichlids and Nile tilapia, and both these cases further support the 'library' hypothesis [27].

**Figure 7:**
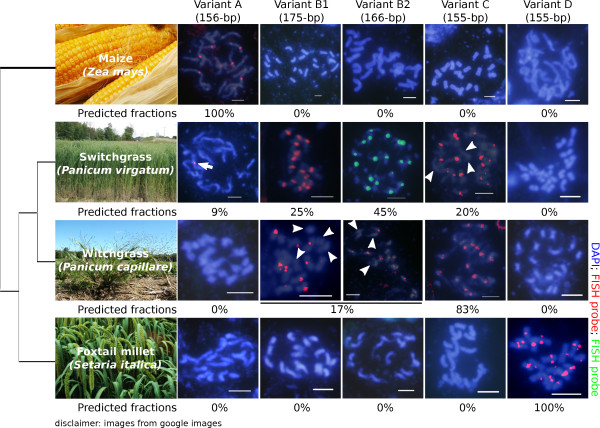
**Chromosomal localization of repeat variants in grasses is consistent with repeat abundance measured by our bioinformatic pipeline**. Chromosomal localization of the different grass repeat variants (maize variant A, switchgrass variants B1 and B2, witchgrass variant C, and foxtail millet variant D) was determined by FISH on metaphase chromosomes of maize (*Zea mays*), switchgrass (*Panicum virgatum*), witchgrass (*Panicum capillare*), and foxtail millet (*Setaria italica*). Switchgrass variants B1 and B2 differ by a 9-bp deletion, whereas both variants differ from maize, witchgrass and foxtail millet by a 20-bp insertion. Maize and foxtail millet chromosomes hybridized only to variants A and D, respectively. Only one switchgrass chromosome hybridized to variant A (arrow), but variants B1, B2 and C labeled most chromosomes (arrowheads indicate chromosomes that showed weaker hybridization to variant C). Witchgrass chromosomes were most consistently labeled by variant C, but showed chromosome-specific hybridization to variants B1 and B2, consistent with their lower abundance in the genome. In all cases the FISH probes hybridized to the primary constriction, which is indicative of centromere localization. The percentages below the panels represent computational predictions of repeat variant ratios in each species.

**Figure 8:**
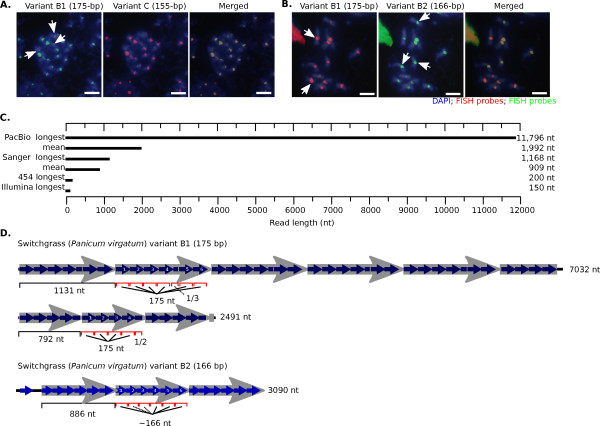
**Pacific Biosciences sequencing shows homogeneity of repeat arrays and detects long higher order repeat structures**. **(a) **Switchgrass variant B1 hybridized to all switchgrass chromosomes, whereas witchgrass variant C hybridized to all but three switchgrass chromosomes. The three chromosomes that only showed hybridization of variant B1 (arrows) were stained green (see merged). **(b) **Although both switchgrass variants B1 and B2 co-hybridize to all switchgrass chromosomes, the hybridization signal showed a chromosome-specific pattern. The arrows highlight chromosomes with stronger hybridization signal for one sub-variant over the other. **(c) **The strength of PacBio sequencing is the extreme length of a small fraction of the reads. In the AP13 switchgrass PacBio sequencing run, the longest inserted sequence was almost 12 kbp in length, although the mean of all the PacBio reads was about 2 kbp. Sanger reads are shorter, but have a more consistent length, whereas both Illumina and 454 reads are very short and very homogeneous in length (longest reads in our study only shown). **(d) **Although no repeat variant mixing was detected in the PacBio reads, several HOR structures were found in longer PacBio reads. These HOR structures consisted of a mixture of complete and trunctated repeats. Two switchgrass variant B1 centromere reads with higher order structure and one switchgrass variant B2 centromere repeat are shown. The 1,131-bp HOR structure consisted of six repeat monomers and a truncated repeat (about one-third the size of 175 bp repeat). In total, five-and-half copies of the 1,131-bp repeat were found within the 7 kbp read. One variant B2-containing read is shown, containing three copies of a 886-bp HOR structure (composed of six 166-bp repeats).

Next we asked if switchgrass repeat variants occupied the same tandem repeat arrays by using computationally derived repeat monomers as probes in FISH experiments. FISH analysis confirmed that these repeat variants were found at centromeres (Figure 7). Variants not found in a given genome did not stain chromosomes from that species, showing that our hybridization conditions were specific. The variant A probe only hybridized strongly to one switchgrass chromosome. Variant B in switchgrass was composed of two repeats (B1 = 175 bp and B2 = 166 bp) and FISH experiments revealed that all switchgrass chromosomes showed hybridization to variants B1, B2 and C, but with differing hybridization intensities (Figure 8a,b). These data indicate that specific chromosomes harbor different amounts of particular repeat variants, again suggesting that repeat arrays can grow and shrink over evolutionary time.

### Pacific Biosciences sequencing reveals that switchgrass repeat arrays are homogeneous and contain long higher order repeat structures

Centromere repeat variants in switchgrass were found on the same chromosomes using FISH (Figure 8a,b), but the resolution of these experiments could not distinguish large homogeneous arrays of two variants (in close proximity) from arrays that showed more significant mixing of repeat variants. Theoretical simulations predict that an array of polymorphic repeats can become rapidly homogenized by unequal crossing over [52]. Conversely, gene conversion can introduce novel variants into the middle of a repeat array. To determine the degree to which variants were mixed in a given array, we used the PacBio sequencing platform, which yields much longer reads (up to 16.5 kbp) than other sequencing technologies (Figure 8c) [56]. As PacBio sequencing has a very high indel rate, we focused on repeat variants that differ by indels of at least 9 bp. Switchgrass genomic DNA was sequenced on four runs of the PacBio RS system using the C2 chemistry and an approximately 10-kbp insert library (see Materials and methods for details). All switchgrass chromosomes stained positive for both variant B1 and B2 FISH probes and both repeat variants were present in the PacBio sequence data. However, individual PacBio sequencing reads never contained a mixture of the two variants. This shows that centromere repeat arrays in switchgrass are composed of long homogeneous array variants, but that these arrays are mixed together on the same chromosome.

Another benefit of PacBio sequence reads is their ability to detect HOR structure that extends beyond the dimer and trimer structures typically visible in shorter Sanger reads (Figure 6). We found a novel pattern of HOR structure in switchgrass centromeres using PacBio sequencing: large repeating units that contain deleted versions of a canonical centromere repeat (Figure 8d). A 2,491-bp read contained a higher order repeat composed of four B1-type monomers followed by a truncated variant approximately half the size of the B1 repeat. The B1 repeat is 175 bp long, and the HOR repeat is 792 bp, too long to be detected by Sanger sequencing. Similarly, a 7,032-bp PacBio read contained a 1,131-bp HOR repeat made of six B1-type monomers and a truncated B1 repeat of 53 bp. In this case, the HOR repeat itself is longer than almost all Sanger sequence reads. This application shows that long reads have the benefit of directly revealing long repeat structures that could previously only be seen through painstaking and indirect assembly strategies or by chromosome-specific cytogenetic methods [11,43,49,57].

## Discussion

The ready availability of WGS sequence from a wide variety of eukaryote genomes makes comparative genomics an appealing way to study rapidly evolving tandem repeat sequences, such as those commonly associated with centromeres. Animals and plants are evolutionarily distant, so previous studies showing the presence of high-copy centromere tandem repeats in these organisms raised the question of whether this was indeed a general property. Recently, bioinformatic methods for identifying centromere tandem repeats have been described, and applied to several previously uncharacterized mammals [33,58,59] and plants [60]. We have performed the largest survey of animal and plant tandem repeats to date, encompassing every species with sufficient WGS sequence in the NCBI trace archive and DDBJ sequence read archive. The bioinformatic methods we used are amenable to every available DNA sequencing technology, making our study expandable as future DNA sequences are generated. In species with previously reported centromere repeats, the most abundant tandem repeat identified in our analysis matched the published sequence in almost every case. The presence of highly abundant tandem repeats in the large majority of species that we analyzed suggests that tandem repeats likely underlie the functional centromere in most animals and plants. Candidate centromere tandem repeats did not share conserved properties such as monomer length, GC content, or common sequence motifs. We found that higher-order tandem repeat structures were prevalent across a broad phylogenetic distribution, as was the evolution of repeats by mutation and indel acquisition. This confirms theoretical predictions that the tandem repeat nature of centromere DNA in animals and in plants can facilitate the rapid evolution of these sequences [52].

As centromeres can form on non-centromeric DNA sequences in both animals and plants, the function of tandem repeats at centromeres is enigmatic [12,13,17,61]. Our finding that centromere tandem repeats are common reinforces the argument that they have a functional, albeit subtle, role, although careful experiments may be required to detect this *in vivo*. Further evidence for this comes from both evolutionary and functional experiments. Neocentromeres formed during evolution eventually acquire tandem repeats [62], and neocentromeres lacking tandem repeats are subtly defective in one human cell culture assay [63]. It is possible that centromere specification will be a balance between epigenetic and genetic factors in most plants and animals, although it is clear that epigenetic memory provided by the centromere-specific histone CENH3 is the most important factor.

High-copy tandem repeats have a propensity to form heterochromatin [64], but it is unlikely that this property alone explains their presence at centromeres. Transposons in pericentromeric regions are also highly heterochromatic, and there is little in the chromatin landscape of large repeat-rich genomes such as maize that distinguishes centromeres from similarly gene-poor regions. Transposons inserted into the tandem repeat arrays of cereals and other plant genomes have not been shown to have a function in centromere biology, although they are bound by CENH3 [46,65,66] and centromere-specific transposons localize exclusively to the centromeres of close relatives [67]. Most interestingly, the tandem repeats within the CENH3-binding domain of the centromere have significantly different chromatin modifications from typical heterochromatin [68]. In *A. thaliana *and maize, tandem repeats at the functional centromere have been observed to have lower DNA methylation than those at the edge of the repeat array [69]. Extended chromatin fiber microscopy has shown that centromeres in *Drosophila melanogaster *and humans contain some modifications typical of euchromatin (for example, lack of H3K9 di- or trimethylation), in addition to those associated with gene silencing (hypoacetylation of H3 and H4) [68]. Tethering a transcriptional silencer to a human artificial chromosome or altering its acetylation/methylation balance can lead to centromere inactivation [70,71]. Lastly, it is possible that non-coding RNAs may have a role in centromere function, and transcription of such molecules may not be compatible with heterochromatic marks [39,72-74].

If specific DNA sequences play a role at centromeres, and heterochromatin is not needed for kinetochore function, why do so many animal and plant centromeres contain high-copy tandem repeats? The lack of conserved properties among these sequences suggests that it is the tandem nature of the repeats that in itself is useful. Nucleosome phasing may be beneficial for centromeres, and the sequence preferences of histones should lead to phasing on any tandem repeat even if this is a subtle property. Although one study failed to detect nucleosome phasing (translational positioning) at the maize centromere tandem repeats, periodicity based on AA/TT dimers (rotational positioning) within CentC repeats, which suggests that CentC repeats could contribute to a highly stable nucleosome arrangement in centromeres [75]. Nucleosome phasing over the entire centromere should be dominated by nucleosomes containing conventional histone H3, as CENH3 nucleosomes bind to only a small fraction of the tandem repeat array. In a phasing model, the acquisition and accumulation of tandem repeat arrays would be fostered by the chromatin arrangement of centromeres. The phenomenon of centromere reactivation, in which a centromere first loses kinetochore-nucleating activity and then regains it, could suggest that tandem repeats encourage centromeric chromatin states. Notably, centromere reactivation has been observed in both maize [76,77] and possibly in humans [78].

Rapid evolution itself may explain the fact that centromere DNA in so many animals and plants is composed of tandem repeats. A prevailing model to explain fast evolution of centromere DNA sequences and CENH3 is that asymmetric meiosis during oogenesis encourages centromeric drive [4,79]. In this model, competition of centromeres for preferential segregation into the single meiotic cell that survives to become the egg can drive rapid sequence evolution. Eventually, centromere DNA and CENH3 differences could introduce reproductive barriers, causing speciation. CENH3 binding domains in animal and plant chromosomes cover many kilobase pairs of DNA. How is it possible that these large stretches of DNA could co-evolve with a histone H3 variant? Similarly, how do centromere DNA sequences on different chromosomes co-evolve? In a tandem repeat array, CENH3 is necessarily binding to the same sequences throughout the centromere, and all chromosomes in the cell typically share versions of the same repeat monomer [80]. In addition, tandem repeats foster rapid evolution, and this property may be favored by meiotic drive [4,52]. A mutation that arises in any copy of a tandem repeat can be amplified and spread throughout the array by unequal crossing over [52] or by replication fork collapse [81]. Repeat variants can move between different chromosomes in the cell via gene conversion, or possibly through the mobilization of retrotransposons inserted into tandem repeat arrays [82,83]. As we have shown, the centromere tandem repeat array can be a 'library' of sequence variants that show expansion and shrinkage [53,54], creating opportunities for new variants to colonize a chromosome, likely via concerted evolution or molecular drive [84]. Centromeres with sequence differences would be immediately exposed to selection in organisms with asymmetric female meiosis. Thus, the ability of tandem repeats to facilitate concerted evolution may explain their prevalence at animal and plant centromeres. Yeast species with symmetrical meiosis lack high copy tandem repeats at centromeres [5]. Similarly, the centromere-specific histone does not show positive selection in *Tetrahymena *species with symmetrical meiosis [85]. In the future, it will be interesting to test whether tandem repeats are found at centromeres of diverse eukaryotes that lack asymmetric meiosis.

## Conclusions

Our study is the largest survey of tandem repeats in eukaryotes. We identified tandem repeats from reads of widely varying lengths. It has to be noted that the most definitive verification of centromeric localization of tandem repeats (ChIP with an antibody against the fast evolving CENH3 protein) was not realistically feasible at the scale of this study. Therefore, we validated our results to published work (Table S2 in Additional file 3). Overall, our results indicate that tandem repeats are highly prevalent at centromers of animal and plant genomes, yet we found no sequence similarity between repeats from species that diverged more than 50 MYA. This suggests a substable yet functional role for such repeats, perhaps in promoting concerted evolution of centromere DNA across chromosomes.

## Materials and methods

### Obtaining sequence data from online archives

Only WGS or whole chromosome shotgun (WCS) data were used in our analysis. Sanger DNA sequences (FASTA and corresponding ancillary files) were downloaded from the NCBI Trace Archive [86]. For each of the 170 species with WGS or WCS Sanger data, we downloaded up to 5 randomly selected FASTA files (up to 500,000 sequences/file). Illumina and 454 data were downloaded from the DDBJ Sequence Read Archive [87]. As of 1 April 2012, 146 species had WGS Illumina or 454 data. For these species, two random FASTQ files were downloaded (one per direction, on average 2 Gb/file). For 37 species both Sanger and Illumina data were obtained. A complete list of species, and associated sequence data, that were used in our study can be found in Table S1 in Additional file 3.

### Bioinformatics pipeline for Sanger and Pacific Biosciences data

WGS or WCS data were processed using a Perl-based bioinformatics pipeline. First, Sanger sequences were clipped for quality and/or vector contamination. Subsequently, sequences that had >5% Ns were removed, as were any sequences shorter than 100 bp (Sanger) or 1,000 bp (PacBio). Low complexity sequences were then masked using the DUST filter. The remaining sequences were analyzed by TRF [50] to identify tandem repeats. We assumed that candidate centromeric tandem repeat arrays should be continuous and occupy the majority - if not all - of any individual read. We therefore excluded repeats that accounted for <80% of the entire read. TRF sometimes predicted multiple tandem repeats occupying the same span within a read (with different repeat monomer lengths). In these situations we only retained the shortest repeat for further analysis. Very short repeats, with monomer lengths <50 bp, were also excluded from further analysis.

After producing a set of tandem repeats for each species of interest (using the consensus repeat sequence from TRF), we then used WU-BLASTN [88] with parameters M = 1 N = -1 Q = 3 R = 3 W = 10 (with post-processing from various Perl scripts) to produce a set of 'global' and 'local' clusters of repeats in each species (see Additional file 1 for full details). Global clusters contained repeats with very similar sequences that also had near-identical lengths. This clustering step used just a sample of the total number of tandem repeats produced by TRF and we identified the source reads of all of the sample repeats. This allowed us to identify what fraction of the input sample reads was represented by each global or local cluster. Repeats in the top clusters are presumed to be the candidate centromeric repeat.

### Bioinformatics pipeline for Illumina and 454 data

Illumina and 454 reads are often too short to contain at least two copies of a tandem repeat. Therefore, these shorter reads have to be assembled to create contigs that contain at least two copies of a tandem repeat (even if such contigs are not biologically real). To assemble contigs containing tandem repeats, repeat monomers must be polymorphic (a property shared by all centromere tandem repeats described so far [4]). Some short read assemblers do not work well with sequences containing polymorphisms. To assemble polymorphic centromere tandem repeats, we used the short read assembler PRICE [89]. For most of the Illumina and 454 data we used PRICE beta version 0.6. This version could only handle paired-end Illumina and 454 data. The later PRICE beta version 0.13 and subsequent versions also allowed for use of single end Illumina and 454 data. For each species, we used 200,000 randomly selected reads, which were assembled on 20,000 seed sequences (see PRICE manual) with at least 85% sequence similarity. The contigs were analyzed for the presence of tandem repeats by TRF, allowing for a tandem repeat monomer of 2,000 bp (upper limit of TRF). To determine genomic fraction, 1,000,000 short reads were aligned to the obtained tandem repeat monomers (see Additional file 1 for more details).

### Data analysis of centromere tandem repeats

To compare candidate centromeres from all species to each other, we performed a BLASTN [90] search. We used WU-BLAST version 2.0 with parameters M = 1 N = -1 Q = 2 R = 2 W = 8. Since tandem repeat boundaries are arbitrary, it is possible for related repeats to align in a staggered fashion and align over only a fraction of their true length. We therefore aligned a file of repeats to a file of duplicated repeats. Since BLAST produces local alignments and we were interested in overall similarity, we calculated a global percent identity by adding additional alignment length assuming a 25% match rate in unaligned regions.

To assess the rate at which sequence similarity decays on phylogenetic timescales, we performed node-averaged phylogenetically independent contrasts [91,92]. In order to account for shared history in comparisons of sequence similarity, this method calculates the average sequence similarity between each pair of taxa spanning a node to generate a single value for each node in the tree. Since the taxa of interest span a wide range of eukaryotes and our analyses are relatively insensitive to branch length estimates, we used a tree based on the NCBI taxonomy [93] and repeated our analyses on ten random resolutions of the tree in order to accommodate unresolved relationships. As most unresolved nodes were shallow, these random resolutions had little effect on the quantitative results of the analyses performed (data not shown). All phylogenetic analyses were conducted using the R package APE [94]. We then performed regression analysis in order to determine the relationship between node age (as determined with TimeTree [95]) and node-averaged sequence similarity. We used the R package bbmle2 to fit the simple exponential model H ~ αt^λ^, where H is the node-averaged homology and t is node age, and α is the intercept.

To determine the conservation of several repeat characteristics on a finer scale, we performed phylogenetic comparative analysis using the R packages GEIGER [96] and picante [97]. We estimated Blomberg's *K *measure of phylogenetic conservation for repeat length, GC content, and repeat abundance using chronograms estimated for primates [98] and grasses [99].

### Pacific Biosciences single molecule real time sequencing

Switchgrass (tetraploid *Panicum virgatum *AP13) DNA was isolated using a modified protocol for Chen and Ronald [100] (Additional file 1). Library preparation and sequencing was performed according to the manufacturer's instructions (Pacific Biosciences). In short, 3 to 10 μg of genomic DNA was isolated and fragmented to 7- to 10-kbp fragments using HydroShear for 15 minutes (switchgrass), or Covaris G-tube (cattle, yak, water buffalo). The first of five Ampure XP bead purifications was performed (0.45X Ampure beads added to DNA dissolved in 200 μl EB, vortexed for 10 minutes at 2,000 rpm, followed by two washes with 70% alcohol and finally diluted in elution buffer). After each Ampure XP purification step a quality control was performed consisting of DNA concentration determination by nanodrop and fragment size distribution by bioanalyzer. Next, the DNA fragments were repaired using DNA Damage Repair solution (1× DNA Damage Repeat Buffer, 1× NAD+, 1 mM ATP high, 0.1 mM dNTP, and 1× DNA Damage Repeat Mix with a final volume of 85.3 μl) with a volume of 21.1 μl and incubated at 37°C for 20 minutes. DNA ends were repaired next by adding 1× End Repeat Mix to the solution, which was incubated at 25°C for 5 minutes, followed by the second Ampure XP purification step. Next, 0.75 μM of blunt adapter was added to the DNA, followed by 1× template prep buffer, 0.05 mM ATP low and 0.75 U/μl T4 ligase to ligate (final volume of 47.5 μl) the bell adapters to the DNA fragments. This solution was incubated at 25°C for 30 minutes, followed by a 65°C 10 minute heat-shock. The exonuclease treatment to remove unligated DNA fragments consists of 1.81 U/μl Exo III and 0.18 U/μl Exo IV (final volume of 3.8 μl), which is incubated at 37°C for 1 hour. Next, three Ampure XP purifications steps were performed. Finally, the bell primer is annealed to the PacBio bell with inserted DNA fragment (80°C for 2.5 minutes followed by decreasing the temperature by 0.1°/s to 25C°). This complex was loaded into PacBio RS SMRT cells, which were loaded onto the machine for either 2 × 30, 2 × 45, 1 × 75, or 1 × 90 minute runs. Four cells each were used for *Zea mays, Zea luxurians, Panicum virgatum, Bos taurus taurus, Bos taurus indicus, Bos grunniens, Bison bison *and *Bubalus bubalis*, while two cells were sufficient for *Panicum capillare*.

### Fluorescence *in situ *hybridization

Mitotic chromosome spreads were generated following a protocol by Zhang and colleagues [101] with a few modifications (Additional file 1). Plasmid vectors containing a single copy of each repeat variant (A, B1, B2, C, or D) were synthesized by Bio Basic Inc. (Ontario, Canada) and used as probes for FISH analyses. Probe hybridization signals were detected using anti-digoxigenin (dig) conjugated FITC (green), anti-dig conjugated Rhodamine (red), or Streptavidin conjugated Rhodamine (red) antibodies (Roche Applied Sciences, Indianapolis, IN, USA). Chromosomes were counter-stained with 4',6-diamidino-2-phenylindole (DAPI). Digital images were recorded using an Olympus BX51 epifluorescence microscope (Olympus Corporation, Center Valley, PA, USA) (see Additional file 1 for more details).

### Data access

PacBio sequences for *Panicum capillare *and *Panicum virgatum *were deposited in the NCBI Sequence Read Archive under accession number SRA052051. A list of GenBank and Sequence Read Archive accession numbers for all sequences used in this study are provided in Additional file 1. A spreadsheet containing all of the tandem repeat information for each species in this study, along with copies of all Perl scripts used, are available to download online [102].

## Abbreviations

CENH3: centromere-specific histone variant H3; ChIP: chromatin immunoprecipitation; FISH: fluorescent *in situ *hybridization; HOR: higher order repeat; MYA: million years ago; PacBio: Pacific Biosciences; PRICE: Paired-Read Iterative Contig Extension; TRF: Tandem Repeats Finder; WCS: whole chromosome shotgun; WGS: whole genome shotgun.

## Authors' contributions

DM, KB, NT, JR-I, IK and SC designed experiments and interpreted bioinformatic results. DM, KB, NT, MM and IK performed bioinformatic analyses. HY performed FISH experiments. GR and JD provided the PRICE short read assembler. DM, RS, PP, JE and DR performed PacBio sequencing. JG contributed genome sequence data for Nellore. TS contributed genome sequence data for cattle, bison, yak and water buffalo. DM, KB, JR-I, IK and SC wrote the paper, with substantial assistance from HY, TS and CT. All authors read and approved the final manuscript.

## Supplementary Material

Additional file 2 **Supplementary figures**. Click here for file

Additional file 3 **Supplementary tables**. Click here for file

Additional file 1 **Supplemental methods**. Click here for file
