# Supplementary material for: Comparative analysis of tandem repeats from hundreds of species reveals unique insights into centromere evolution
Source: Genome Biol. 2013 Jan 30;14(1):R10. doi: 10.1186/gb-2013-14-1-r10 (PMC4053949; doi:10.1186/gb-2013-14-1-r10)
Supplement: Additional file 2 — Supplementary figures. [file gb-2013-14-1-r10-S2.PDF]

# Supplementary Figure S1

```
Orange

Citrus_sinensis        CATTCCGAGTCCGGCGGACGAACTTCGCCCACGCCCCCCACCAAGGCTATAGCCCACCCG 60
Citrus_clementina      CATTCCGAGTCCGGCGGACGAAGTTGGCCAGCCCCACCCCCCAAGGCTATAGCCCACCCG 60
                       ********************** ** ***  * ** *** ********************

Citrus_sinensis        ATTTT-TGGCCATTTTTCCGCTGG-CGAGTCTTGGCGCCCCGACCTTCGGGCGCTCATTT 118
Citrus_clementina      TTTTTGTGGCC-TTTTTCCGCTGGACGAA-CTTGGCGCCCCGGCCTTCGGGCGGTTATTT 118
                        **** ***** ************ ***  ************ ********** * ****

Citrus_sinensis        TTGGGCGCGGCTGTGCCCGTGGCCTATTTTTGGCACACGGAGGCCGCCCGCAAAGTCTGG 178
Citrus_clementina      TTGGGCGCGGCTTTGCCCGCGGCCTATTTTTGGCACACGGAGGCCCCTCGCAAAGTCTCG 178
                       ************ ****** ************************* * ********** *

Citrus_sinensis        GGC 181
Citrus_clementina      CGG 181
                        *
Candidate centromere repeat monomer:
>Citrus_sinensis
CATTCCGAGTCCGGCGGACGAACTTCGCCCACGCCCCCCACCAAGGCTATAGCCCACCCG
ATTTTTGGCCATTTTTCCGCTGGCGAGTCTTGGCGCCCCGACCTTCGGGCGCTCATTTTT
GGGCGCGGCTGTGCCCGTGGCCTATTTTTGGCACACGGAGGCCGCCCGCAAAGTCTGGGG
C
>Citrus_clementina
CATTCCGAGTCCGGCGGACGAAGTTGGCCAGCCCCACCCCCCAAGGCTATAGCCCACCCG
TTTTTGTGGCCTTTTTCCGCTGGACGAACTTGGCGCCCCGGCCTTCGGGCGGTTATTTTT
GGGCGCGGCTTTGCCCGCGGCCTATTTTTGGCACACGGAGGCCCCTCGCAAAGTCTCGCG
G

#################################################################################################
Arabidopsis

Arabidopsis_lyrata        TTGCTTCTCAAATCTTTGTGGGTGTGGCCGAAGTCC-TATGAGTTTTCGGTTTTGGAGCT 59
Arabidopsis_thaliana      TTGCTTCTCAAAGCTTTCATGGTGTAGCCAAAGTCCATATGAGTCTTTGGCTTTGTGTCT 60
                          ************ ****   ***** *** ****** ******* ** ** ****   **

Arabidopsis_lyrata        TCTAAACGGAAAAACACTACTTTAGCTTTCGGGATCCGGTTGCGGCTCTAGTTCTTATAC 119
Arabidopsis_thaliana      TCTAA-CAAGGAAACACTACTTAGGCTTTTAAGATCGGGTTGCGGTTTAAGTTCTTATAC 119
                          ***** *    ***********  *****   **** ******** *  ***********

Arabidopsis_lyrata        CCAATCATAAACACGAGATCTAGTCATATTTGACTCCAAAAACACTAACCAAGCTTCTTA 179
Arabidopsis_thaliana      TCAATCATACACATGACATCAAGTCATATTCGACTCCAAAA-CACTAACCAACCTTCTTC 178
                           ******** *** ** *** ********* ********** ********** ******
Centromere repeat monomers:
>Arabidopsis_lyrata
TTGCTTCTCAAATCTTTGTGGGTGTGGCCGAAGTCCTATGAGTTTTCGGTTTTGGAGCTT
CTAAACGGAAAAACACTACTTTAGCTTTCGGGATCCGGTTGCGGCTCTAGTTCTTATACC
CAATCATAAACACGAGATCTAGTCATATTTGACTCCAAAAACACTAACCAAGCTTCTTA
>Arabidopsis_thaliana
TTGCTTCTCAAAGCTTTCATGGTGTAGCCAAAGTCCATATGAGTCTTTGGCTTTGTGTCT
TCTAACAAGGAAACACTACTTAGGCTTTTAAGATCGGGTTGCGGTTTAAGTTCTTATACT
CAATCATACACATGACATCAAGTCATATTCGACTCCAAAACACTAACCAACCTTCTTC

#################################################################################################
Eucalyptus

Eucalyptus_grandis       ATTTTGGAGATCCCGATACCTTGAAAGTCGGCTGAAATCAAGAAACGGAGACTCGGCACA 60
Eucalyptus_globulus      ATTTTGGACATCACAGCACCCTGAACGCCCGGCGAAACAAGAGAACGAAGATTCCACAGA 60
                         ******** *** *   *** **** * * *  ****  *   **** *** **  ** *

Eucalyptus_grandis       AAAAATTCCCTTGTGTTCCATTAAAAATTCCTTTTCGG----TATTTTCACT-ATAGACC 115
Eucalyptus_globulus      GAAAATTCCCTGTTTTTCCATAGAAAATTCCCTTTCCGGCCATTTTTTGGCTGAAAAACC 120
                          **********  * ******  ******** **** *    * ****  ** * * ***

Eucalyptus_grandis       CCGGCAGGCGGAAA-CGGCATGGATAAGTGGGCTCCAAAAGCTTTATTTCGATCGGATTT 174
Eucalyptus_globulus      CCGACAGTTGGAAATCTGTTGGGAAACATGGCTTCCAAAAGCATTAGATCAGTCCGACCC 180
                         *** ***  ***** * *   *** *  ***  ********* ***  **  ** **
```

```
Eucalyptus_grandis       AAT 177
Eucalyptus_globulus      ATC 183
                         *
Candidate centromere repeat monomer:
>Eucalyptus_grandis
ATTTTGGAGATCCCGATACCTTGAAAGTCGGCTGAAATCAAGAAACGGAGACTCGGCACA
AAAAATTCCCTTGTGTTCCATTAAAAATTCCTTTTCGGTATTTTCACTATAGACCCCGGC
AGGCGGAAACGGCATGGATAAGTGGGCTCCAAAAGCTTTATTTCGATCGGATTTAAT
>Eucalyptus_globulus
ATTTTGGACATCACAGCACCCTGAACGCCCGGCGAAACAAGAGAACGAAGATTCCACAGA
GAAAATTCCCTGTTTTTCCATAGAAAATTCCCTTTCCGGCCATTTTTTGGCTGAAAAACC
CCGACAGTTGGAAATCTGTTGGGAAACATGGCTTCCAAAAGCATTAGATCAGTCCGACCC
ATC

##########################################################################################
Figs

Ficus_fistulosa         TTAAGCCAGAATCAAGCATAAAGGGACC-AAAATG-AGCAAATCTCAGTTCAAGGCACAG 58
Ficus_langkokensis      TCAAGCCCGAATGAGGCAAAAAGTGACCCAAAATGCAATGTAA-TCAGATCAAGGTCGTG 59
                        * ***** **** * *** **** **** ****** *    *  **** ******    *

Ficus_fistulosa         TGGATCATCTAGGCAGCCCCTAAATGACCAAAACAAGCTTTGAGAATGATCAAAACACAA 118
Ficus_langkokensis      TAAACCAGTGAGGTGGCCTGTAAATGACCTAAACAAGGTTTGACAAGGATCAAAACACAA 119
                        *  * **   ***  ***  ********* ******* ***** ** *************

Ficus_fistulosa         AAAAACCAGACCCAATCTGGTTTGGGTCAAAAAAGTGGCAAAA 161
Ficus_langkokensis      AAAA-CCAGATCCTAACAACTTTGGGTCAAGAAACCAGCAAAA 161
                        **** ***** ** * *   ********** ***   ******

Candidate centromere repeat monomer:
>Ficus_fistulosa
TTAAGCCAGAATCAAGCATAAAGGGACCAAAATGAGCAAATCTCAGTTCAAGGCACAGTG
GATCATCTAGGCAGCCCCTAAATGACCAAAACAAGCTTTGAGAATGATCAAAACACAAAA
AAACCAGACCCAATCTGGTTTGGGTCAAAAAAGTGGCAAAA
>Ficus_langkokensis
TCAAGCCCGAATGAGGCAAAAAGTGACCCAAAATGCAATGTAATCAGATCAAGGTCGTGT
AAACCAGTGAGGTGGCCTGTAAATGACCTAAACAAGGTTTGACAAGGATCAAAACACAAA
AAACCAGATCCTAACAACTTTGGGTCAAGAAACCAGCAAAA

##########################################################################################
Soybean

Glycine_max       TCACTCGGATGTCCGATTCAGGCGCATAATATATCGAGACGCTCGAAATTGAACAACGGA 60
Glycine_soja      TCACTCGGATGTCCGATTCAGGCGCATAATATATCGAGACGCTCGAAATTGAACAACGGA 60
                  ************************************************************

Glycine_max       AGCTCTCGAGAAATTCAAATGATCATAACTTT 92
Glycine_soja      AGCTCTCGAGAAATTCAAATGGTCATAACTTT 92
                  ********************* **********

Centromere repeat monomer:
>Glycine_max
TCACTCGGATGTCCGATTCAGGCGCATAATATATCGAGACGCTCGAAATTGAACAACGGA
AGCTCTCGAGAAATTCAAATGATCATAACTTT
>Glycine_soja
TCACTCGGATGTCCGATTCAGGCGCATAATATATCGAGACGCTCGAAATTGAACAACGGA
AGCTCTCGAGAAATTCAAATGGTCATAACTTT

##########################################################################################
Beechs 1

Lithocarpus_calolepis           TATTTTGGTCATTTTTTGGGTTTCGGGGGTATTTTGGTCATTTTTTGGGT 50
Lithocarpus_grandilofolius      TATTTTGGTAATTTTTTTG-TTTCGAGGGTATTTTGGTCATTTTT-AGGT 48
                                ********* ******* * ***** *******************  ***

Lithocarpus_calolepis           TTCGGGAGTATTTTGGTCATTTTT-AGGTTTCGGGGGTTATTTTGGTAAT 99
Lithocarpus_grandilofolius      TTCGGGGGTATTTTGGTCATTTTTTGGGTTTCGGGGGT-ATTTTGGTCAT 97
                                ****** *****************  ************ ******** **
```

```
Lithocarpus_calolepis           TTTT-AGGTTTCGGGGG 115
Lithocarpus_grandilofolius      TTTTTGGGTTTCGGAGG 114
                                ****  ******** **

Candidate centromere repeat monomer:
>Lithocarpus_calolepis
TATTTTGGTCATTTTTTGGGTTTCGGGGTATTTTGGTCATTTTTTGGGTTTCGGGAGTA
TTTTGGTCATTTTTAGGTTTCGGGGGTTATTTTGGTAATTTTTAGGTTTCGGGGG
>Lithocarpus_grandilofolius
TATTTTGGTAATTTTTTTGTTTCGAGGGTATTTTGGTCATTTTTAGGTTTCGGGGGTATT
TTGGTCATTTTTTGGGTTTCGGGGGTATTTTGGTCATTTTTTGGGTTTCGGAGG

##############################################################################################################
Beechs 2

Lithocarpus_balansae        TTTGAGCCCTTACATCGGCACGAAATTGGAATCCAAATTTCGGGTAACTC 50
Lithocarpus_hancei          TTTGAGCCCTTATATCGGCACAAAATTGGAATCCAACTTTCGGGTAACTC 50
Lithocarpus_xylocarpus      TTTACGCCTTTATATCGGCACAAAATTGGAGTCCAAATTTCGGGGAACTC 50
                            ***  *** *** ******** ******** ***** ******* *****

Lithocarpus_balansae        GGTGGCCCACATTCAAACGAGGATAACTCTCCCGATTTTTATCAAAAAAA 100
Lithocarpus_hancei          AGTGGCCCATATTCAAACGATAATAACTCTCATGATTTTTGTCGAAAAAA 100
Lithocarpus_xylocarpus      GGTGGCCCATATTCAAACGACAATAACTCTCTTGATTTTTATCGAAAAAA 100
                             ******** **********  *********  ******* ** ******

Lithocarpus_balansae        TACAAAATTTGTGCTCAAATTCAAGCTCAGGATGTCTACTTTCTAAAACA 150
Lithocarpus_hancei          TACAAAATTTGTGTTCAAATTCAAGCTCAGGACGTCTACTTTCTAAAACA 150
Lithocarpus_xylocarpus      CACCAAATTTGTGTTCAAATTCAAGCTCAGAATGTCTACTTTCTAAAATA 150
                             ** ********* *************** * *************** *

Lithocarpus_balansae        CGAAGGCCGCGTCAAAGAATTCCTCACGGTTCAAAAGTTATTGACGAAAC 200
Lithocarpus_hancei          CTTAGGCCGCGTTAAAGAATTCCTTTCGGTTCAAAAGTTATTGACGAAAC 200
Lithocarpus_xylocarpus      TTAAGGCCGCGTTGAAGAATTCCCTACGGTTCAAA-GTTATTGTCGAAAG 199
                               *********  *********   ********* ******* *****

Lithocarpus_balansae        GGTGGCCAAAGCACACTTTTCGTCACACTTCCTAACCGATTAACGCCCGC 250
Lithocarpus_hancei          GGTGGCCAAAGGTCACTTTTCGTCTCATTTCCTAACCGATTAACGCTCGT 250
Lithocarpus_xylocarpus      GGCGACCAAAGGTCACTTTTCGACACATTTCCAAACCGATGAAGGCTCGT 249
                            ** * ******  ********* * ** **** ******* ** ** **

Lithocarpus_balansae        GGATAACTACCCACTCACGTATTTTTTCCCCGAAACTTGATTTTGGGAAG 300
Lithocarpus_hancei          GGATAACAACCCACTCACATATTTTTTCCCCGAAACTTGATTTTGGGAAG 300
Lithocarpus_xylocarpus      GAATAACTACCCACCAAAGTATTTTTTACACAAAACTTCACTTTGAGAGG 299
                            * ***** ******  *  ******** * * ****** * **** ** *

Lithocarpus_balansae        ATTGATCTCGCGATATAGGAAAAATATTTCCCAGCCAAATTCGTGAGCAA 350
Lithocarpus_hancei          ATTGATCTCGCGATGTAGGAAAAATATTTCATGCACAAATTCGTGAGCAA 350
Lithocarpus_xylocarpus      ATTGACCTCAAGATATAGAAAAAAATATTTCATCGCCAAATCCGAGAAAAA 349
                            ***** ***  *** *** ***********     ***** ** **  **

Lithocarpus_balansae        AACTTCAAACTTCCCCT 367
Lithocarpus_hancei          AACCACAGACTTCTCCT 367
Lithocarpus_xylocarpus      AACCTTAGACATCAATG 366
                            ***   * ** **

Candidate centromere repeat monomer:
>Lithocarpus_balansae
TTTGAGCCCTTACATCGGCACGAAATTGGAATCCAAATTTCGGGTAACTCGGTGGCCCAC
ATTCAAACGAGGATAACTCTCCCGATTTTTATCAAAAAAATACAAAATTTGTGCTCAAAT
TCAAGCTCAGGATGTCTACTTTCTAAAACACGAAGGCCGCGTCAAAGAATTCCTCACGGT
TCAAAAGTTATTGACGAAACGGTGGCCAAAGCACACTTTTCGTCACACTTCCTAACCGAT
TAACGCCCGCGGATAACTACCCACTCACGTATTTTTTCCCCGAAACTTGATTTTGGGAAG
ATTGATCTCGCGATATAGGAAAAATATTTCCCAGCCAAATTCGTGAGCAAAACTTCAAAC
TTCCCCT
>Lithocarpus_hancei
TTTGAGCCCTTATATCGGCACAAAATTGGAATCCAACTTTCGGGTAACTCAGTGGCCCAT
ATTCAAACGATAATAACTCTCATGATTTTTGTCGAAAAAATACAAAATTTGTGTTCAAAT
TCAAGCTCAGGACGTCTACTTTCTAAAACACTTAGGCCGCGTTAAAGAATTCCTTTCGGT
TCAAAAGTTATTGACGAAACGGTGGCCAAAGGTCACTTTTCGTCTCATTTCCTAACCGAT
TAACGCTCGTGGATAACAACCCACTCACATATTTTTTCCCCGAAACTTGATTTTGGGAAG
ATTGATCTCGCGATGTAGGAAAAATATTTCATGCACAAATTCGTGAGCAAAACCACAGAC
```

```
TTCTCCT
>Lithocarpus_xylocarpus
TTTACGCCTTTATATCGGCACAAAATTGGAGTCCAAATTTCGGGGAACTCGGTGGCCCAT
ATTCAAACGACAATAACTCTCTTGATTTTTATCGAAAAAACACCAAATTTGTGTTCAAAT
TCAAGCTCAGAATGTCTACTTTCTAAAATATTAAGGCCGCGTTGAAGAATTCCCTACGGT
TCAAAGTTATTGTCGAAAGGGCGACCAAAGGTCACTTTTCGACACATTTCCAAACCGATG
AAGGCTCGTGAATAACTACCCACCAAAGTATTTTTTACACAAAACTTCACTTTGAGAGGA
TTGACCTCAAGATATAGAAAAAATATTTCATCGCCAAATCCGAGAAAAAAAACCTTAGACA
TCAATG

#################################################################################################
Potato

Solanum_tuberosum     CGTTAAGACCTTAGCTATGGAGCCAGTTAGCCCTCACGGCCAAAACGTCCCATTTTAAAG 60
Solanum_phureja       CGTTAAGACCTTAGCTATGGAGCCAGTTAGCCCTCACGGCCAAAACATCCCATTTTGAAG 60
                      ********************************************** ********* ***

Solanum_tuberosum     GTCAAATGTGCCCCAAATCAGGAAAACCCC-AATTTTGTCGATTTTCGTATGCTATAGTC 119
Solanum_phureja       GTCAAATGTGCCCCAGAGCAGGTAAACCCCCAATTTTGCCGATTTTCGTGTGCTATAGTC 120
                      *************** * **** ******* ******* ********** **********

Solanum_tuberosum     CATGGACTTTTTGGTGATCTGGAATTCCGACAAAATTTT-GCCAAAATTTTTCGTGGACG 178
Solanum_phureja       CATGGACTTTTTGGTGATCTGGAATTCCGACATAATTTTTGCCAAAATTTTTCATGGACG 180
                      ******************************** ****** ************* ******

Solanum_tuberosum     TC 180
Solanum_phureja       TC 182
                      **

Candidate centromere repeat monomer:
>Solanum_tuberosum
CGTTAAGACCTTAGCTATGGAGCCAGTTAGCCCTCACGGCCAAAACGTCCCATTTTAAAG
GTCAAATGTGCCCCAAATCAGGAAAACCCCAATTTTGTCGATTTTCGTATGCTATAGTCC
ATGGACTTTTTGGTGATCTGGAATTCCGACAAAATTTTGCCAAAATTTTTCGTGGACGTC
>Solanum_phureja
CGTTAAGACCTTAGCTATGGAGCCAGTTAGCCCTCACGGCCAAAACATCCCATTTTGAAG
GTCAAATGTGCCCCAGAGCAGGTAAACCCCCAATTTTGCCGATTTTCGTGTGCTATAGTC
CATGGACTTTTTGGTGATCTGGAATTCCGACATAATTTTTGCCAAAATTTTTCATGGACG
TC

#################################################################################################
Monkeyflower

Mimulus_guttatus        CTTTGATCATTTTTGCTTCCTGGACACCTAATTTTCGATTTGTACCTAAAAGATACCAAA 60
Mimulus_laciniatus      CTTTGATCATTTTTGCTTCCTGGACACCTAATTTTCGATTTGTACCTATAAGATACCAAA 60
Mimulus_dentilobus      CTTTGATCATTTTTGCTTCCTCGACACCTAATTTTCGATTTGTACCTATAAGATACCAAA 60
                        ********************* ************************** ***********

Mimulus_guttatus        TCCACCAATAAAAACAGAAATATAAATTAATCCAAACAAAAATCCACGTAAACGTAAACG 120
Mimulus_laciniatus      TCCACCAATTAAAACAGAAATATAAATTAATCCAAACAAAAATCCACGTAAACGTAAACG 120
Mimulus_dentilobus      TCCACCAATTAAAACAGAAATATAAATTAATCCAAAAAAAAATCCACGTAAACGTAAACG 120
                        ********* ************************** ***********************

Mimulus_guttatus        TTGAGTGTTATTTCTACACTCCACTGTATCTTTTGTATCTAGTGTTTTTGCTGTTGTTTG 180
Mimulus_laciniatus      TCGAGTTTTATTTCTACACTCCATTGTATCTTTTTTATCTAGTGTTTTTGCTGTTGTTTG 180
Mimulus_dentilobus      TAGAGTGTTATTTCTACACTCCACTGTATCTTTTTTATCTAGTGTTTTTGCTGTTGTTTG 180
                        * **** **************** ********** *************************

Mimulus_guttatus        GTTATGCGTATTAAAGTCCTAGTATTGTAGTTCGTGTTTTTAAGAACGTTGTTGCGAATG 240
Mimulus_laciniatus      TTGATGCGTATTAAAGTCATAGTATTGTAGTTCGTGTTTTTAAGAACGTTGTTGCGGATG 240
Mimulus_dentilobus      TTGATGCGTATTAAAGTCCTAGTATTGTAGTTCGTGATTTTAAGAACGTTGTTGCGAATG 240
                         * *************** ***************** ******************* ***

Mimulus_guttatus        CACTTTTGCGCATGTTTTTTTACATGTACGTTTGTGCATGTCCATTTGAGCGAGTGTGTT 300
Mimulus_laciniatus      CACTTTTGCGCATGTTTTTTTACATGTACGTTTGTGCATGTCCATTTGAGGGAGTGTGTT 300
Mimulus_dentilobus      CACTTTGGCGCATGTTTTTTTACATGTACGTTTGTGCATGTCCATTTGAGCGAGTGTGTT 300
                        ****** ******************************************* *********

Mimulus_guttatus        TGTGCATGTTTGTTGATTTGTATTAATGTCCTAGGATTGTAGTTTGTGTTTTCATGATCC 360
Mimulus_laciniatus      TGTGCATGTTTGTTGATTTGTATTAATGTCCTAGGATTGTAGTTTGTGTTTTCATGATCC 360
Mimulus_dentilobus      TGTGCATGTTTGTTGATTTGTATTAATGTCCTAGGATTGTAGTTTGTGTTTTCATGATCC 360
                        ************************************************************
```

```
Mimulus_guttatus        TTTTTGCGAATGCACTTTTGCGTGAGTACTTTTGTGCATGCTTTTGAACTTTTACTCATG 420
Mimulus_laciniatus      TTTTTGCGAATGCACTTTTGCGTGAGTACTTTTGTGCATGCTTTTGTACTTTTACTTATG 420
Mimulus_dentilobus      TTTTTGCGAATGCACTTTTGCGTGAGTACTTTTATGCATGTTTTTGTACTTTTACTTATG 420
                        ********************************* ****** ***** ********* ***

Mimulus_guttatus        TGCGTATCTTTTCTAGCGAGTGTTTTTGTGCACGTTTCTTGATGCGCATTCATGTCCTCG 480
Mimulus_laciniatus      TGCGTATCTTTTCTAGCGAGTGTTTTTGTGCACGTTTCTTGATGCGCATTCATGTCCTTG 480
Mimulus_dentilobus      TGCGTATCTTTTCTAGCGAGTGTTTTTGTGCACGTTTCATGATGCGCATTCATGTCCTCG 480
                        ************************************** ******************* *

Mimulus_guttatus        AACTGTAGCACGTATTTTCATGATCGCTTTTGGAATCCAATTTTGACCATGTTCGAGTCC 540
Mimulus_laciniatus      AATTGTAGTACGTATTTTCATGATCGCTTTTGTTATCCAATTTTGACCATGTTCGAGTCC 540
Mimulus_dentilobus      AACTGTAGTACGTATTTTCATGATCGCTTTTGGAATCCAATTTTGACCATGTTCGAGTCC 540
                        ** ***** ***********************  **************************

Mimulus_guttatus        GAATTTGGAATTTTGACGTTAGACAGGAATTGTAGCCAACTGAAATAGCTTTCTAATGCC 600
Mimulus_laciniatus      GAATTTGGAATTTTGACGTTAGACGGGAATTGTAGCCCGCTGAAATAGCTTTCTAATGCC 600
Mimulus_dentilobus      GAATTTGGAAATTTGCCATTAGACGGGAATTGTAGCCAACTGAAATAGCTTTCTAATGCC 600
                        ********** **** * ****** ************  *********************

Mimulus_guttatus        ACCGGAACCAGTCGATTTGGAGTCCGGATGAGAAAGTTATGCTCGACTTCGTGGACACTA 660
Mimulus_laciniatus      GCCGGAACCAGTTGATTTGGAGTCCGGATGAGAGAGTTATGCTCGACTTCGTGGACACTA 660
Mimulus_dentilobus      GCCGGAACCAGGTCATTTGGAGTCCGGATGAGAAAGTTATGCTCGACTTCGTGGACACTA 660
                         **********   ******************* **************************

Mimulus_guttatus        CCAAAGATGTTGAGGGATTCTCTCTTCTAATTTGATTTTATTTTCTTAATTGCAACTCTT 720
Mimulus_laciniatus      CCAAAGATGTTGAGGGATTCTCTCTTCTAATTTGATTTTATTTTCTTAATTGCAACTCTT 720
Mimulus_dentilobus      CCAAAGATGTTGAGGGATTCTCTCTTCTAATTTGATTTTATTTTCCTAATTGCAACTCCT 720
                        ********************************************* ************ *

Mimulus_guttatus        TTCTTACC 728
Mimulus_laciniatus      TTCTTACC 728
Mimulus_dentilobus      TTCTTACT 728
                        *******
```

Candidate centromere repeat monomer:

```
>Mimulus_guttatus
CTTTGATCATTTTTGCTTCCTGGACACCTAATTTTCGATTTGTACCTAAAAGATACCAAA
TCCACCAATAAAAACAGAAATATAAATTAATCCAAACAAAAATCCACGTAAACGTAAACG
TTGAGTGTTATTTCTACACTCCACTGTATCTTTTGTATCTAGTGTTTTTGCTGTTGTTTG
GTTATGCGTATTAAAGTCCTAGTATTGTAGTTCGTGTTTTTAAGAACGTTGTTGCGAATG
CACTTTTGCGCATGTTTTTTTACATGTACGTTTGTGCATGTCCATTTGAGCGAGTGTGTT
TGTGCATGTTTGTTGATTTGTATTAATGTCCTAGGATTGTAGTTTGTGTTTTCATGATCC
TTTTTGCGAATGCACTTTTGCGTGAGTACTTTTGTGCATGCTTTTGAACTTTTACTCATG
TGCGTATCTTTTCTAGCGAGTGTTTTTGTGCACGTTTCTTGATGCGCATTCATGTCCTCG
AACTGTAGCACGTATTTTCATGATCGCTTTTGGAATCCAATTTTGACCATGTTCGAGTCC
GAATTTGGAATTTTGACGTTAGACAGGAATTGTAGCCAACTGAAATAGCTTTCTAATGCC
ACCGGAACCAGTCGATTTGGAGTCCGGATGAGAAAGTTATGCTCGACTTCGTGGACACTA
CCAAAGATGTTGAGGGATTCTCTCTTCTAATTTGATTTTATTTTCTTAATTGCAACTCTT
TTCTTACC
>Mimulus_dentilobus
CTTTGATCATTTTTGCTTCCTCGACACCTAATTTTCGATTTGTACCTATAAGATACCAAA
TCCACCAATTAAAACAGAAATATAAATTAATCCAAAAAAAAATCCACGTAAACGTAAACG
TAGAGTGTTATTTCTACACTCCACTGTATCTTTTTTATCTAGTGTTTTTGCTGTTGTTTG
TTGATGCGTATTAAAGTCCTAGTATTGTAGTTCGTGATTTTAAGAACGTTGTTGCGAATG
CACTTTGGCGCATGTTTTTTTACATGTACGTTTGTGCATGTCCATTTGAGCGAGTGTGTT
TGTGCATGTTTGTTGATTTGTATTAATGTCCTAGGATTGTAGTTTGTGTTTTCATGATCC
TTTTTGCGAATGCACTTTTGCGTGAGTACTTTTATGCATGTTTTTGTACTTTTACTTATG
TGCGTATCTTTTCTAGCGAGTGTTTTTGTGCACGTTTCATGATGCGCATTCATGTCCTCG
AACTGTAGTACGTATTTTCATGATCGCTTTTGGAATCCAATTTTGACCATGTTCGAGTCC
GAATTTGGAAATTTGCCATTAGACGGGAATTGTAGCCAACTGAAATAGCTTTCTAATGCC
GCCGGAACCAGGTCATTTGGAGTCCGGATGAGAAAGTTATGCTCGACTTCGTGGACACTA
CCAAAGATGTTGAGGGATTCTCTCTTCTAATTTGATTTTATTTTCCTAATTGCAACTCCT
TTCTTACT
>Mimulus_laciniatus
CTTTGATCATTTTTGCTTCCTGGACACCTAATTTTCGATTTGTACCTATAAGATACCAAA
```

TCCACCAATTAAAACAGAAATATAAATTAATCCAAACAAAAATCCACGTAAACGTAAACG
TCGAGTTTTATTTCTACACTCCATTGTATCTTTTTTATCTAGTGTTTTTGCTGTTGTTTG
TTGATGCGTATTAAAGTCATAGTATTGTAGTTCGTGTTTTTAAGAACGTTGTTGCGGATG
CACTTTTGCGCATGTTTTTTTACATGTACGTTTGTGCATGTCCATTTGAGGGAGTGTGTT
TGTGCATGTTTGTTGATTTGTATTAATGTCCTAGGATTGTAGTTTGTGTTTTCATGATCC
TTTTTGCGAATGCACTTTTGCGTGAGTACTTTTGTGCATGCTTTTGTACTTTTACTTATG
TGCGTATCTTTTCTAGCGAGTGTTTTTGTGCACGTTTCTTGATGCGCATTCATGTCCTTG
AATTGTAGTACGTATTTTCATGATCGCTTTTGTTATCCAATTTTGACCATGTTCGAGTCC
GAATTTGGAATTTTGACGTTAGACGGGAATTGTAGCCCGCTGAAATAGCTTTCTAATGCC
GCCGGAACCAGTTGATTTGGAGTCCGGATGAGAGAGTTATGCTCGACTTCGTGGACACTA
CCAAAGATGTTGAGGGATTCTCTCTTCTAATTTGATTTTATTTTCTTAATTGCAACTCTT
TTCTTACC

#################################################################################################
Grasses

```
Zea_mays               -TTTTTCGCAACGAACATGCCCAATCCACTACTTTAGGTCCAAAACTCATGTTTGGG-GT 58
Zea_luxurians          -TTTTTCGCAACGAACATGCCCAATCCACTACTTTAGGTCCAAAACTCATGTTTGGG-GT 58
Setaria_italica        TTTTTTCGCAACGAACGCATGCAATCTTCCTAATTATACCCTAGAA-CATGTTTGGGAGT 59
Setaria_viridis        TTTTTTCGCAACGAACGCATGCAATCTTCCTAATTATACCCTAGAA-CATGTTTGGGAGT 59
Panicum_capillare      --TTTTCGCAAGGAACGCACCCGATCCACTCCAT-GGACCCAAAACTCATGTTTTGGG-C 56
Panicum_hallii         -TTTTTCGCAACGAACGCACCCGATCCACTCCATTGGACCCAAAACTCATGTTTTGGGGC 59
Panicum_virgatum       -TTTTTCGCAACGAACGCACCCAATCCACCCCATTGGACCCTAAACTCATGTTTTGGTAC 59
Oryza_sativa           --TTTTTGCCACGAACGCACCCAATACACTCCAATATGTCCAAAAATCATGTTTTGG--C 56
                         **** ** * ****     * **   *          ** * *  ******* **

Zea_mays               GATTTC-------------------GCGCAATTTCGTTGCCGCACGTCACCCATTCCGAA 99
Zea_luxurians          GGTTTC-------------------GCGCAATTTCGTTGCCGCACGTCACCCATTCCGAA 99
Setaria_italica        GTTTTT-------------------GAGCA-TTCGGTTCCGGCACGAAAAACGATGC-AA 98
Setaria_viridis        GTTTTT-------------------GGGCA-TTCGGTTCCGGCACGAAAAATGATGC-AA 98
Panicum_capillare      GTTTC--------------------GGACCGTTTCGTTACTGCACGAAAGTCGGTGC-AA 95
Panicum_hallii         GTTTC--------------------GGACCGTTTCGTTACTGCACGAAAGTCGATGC-AA 98
Panicum_virgatum       GTTTCATAGTGTTTGGGTGCATTTGGGATCATTTCGTAACTGCATGAAACTCGGTGC-AA 118
Oryza_sativa           CTTTTT-------------------GAACTTTTTCATTCCGGTAAAAAACATCGCACCCA 97
                         **                     *     **   *  * * *    *       *  *

Zea_mays               AACGGGTA----TCGGGGTGCATACAAA---GCACGAGTTTTTGCCACCGGAACAATTTC 152
Zea_luxurians          AACGGGTG----TCGGGGTGCATACAAA---GCACGAGTTTTTGCCACCGGAACCATTTC 152
Setaria_italica        AACAGGTGCATTTCGG----CACCCGAAT--GCACTATTTTCGGGTAGCGAAAC--TCGG 150
Setaria_viridis        AACGGGTGCATTTCGG----CACCCGAAT--GCACTATTGCCGGGTAGCGAAAC--TCGG 150
Panicum_capillare      AACGGGCC-AA-CTGGTGC-CATT--AAC--GCACAAGT-TCGCTAAACGAAG---TTGC 144
Panicum_hallii         AACTGGCCGAA-CTGGTGC-CATT--AAC--GCACAAGT-TCGCTAAACAAAG---TCGC 148
Panicum_virgatum       AACGGGGTGAA-CTGGTGC-AATT--AAT--GCAAAAGT-TCGTGCCACGAAG---TCAC 168
Oryza_sativa           CGTGTGCCAATATTGG----CATT--AATTGACAAAAGT-TCGCCGCGCGAA----TCAC 146
                            *        **     *    **    **  * *         *  *    *

Zea_mays               TTCG---- 156
Zea_luxurians          TTCG---- 156
Setaria_italica        GTGGA--- 155
Setaria_viridis        GTGGA--- 155
Panicum_capillare      GTCGGAA- 151
Panicum_hallii         GTCGGAA- 155
Panicum_virgatum       GTCGGGA- 175
Oryza_sativa           GAAGTGAG 154
                          *
```

Centromere repeat monomer:
>Zea_mays
TTTTTCGCAACGAACATGCCCAATCCACTACTTTAGGTCCAAAACTCATGTTTGGGGTGA
TTTCGCGCAATTTCGTTGCCGCACGTCACCCATTCCGAAAACGGGTATCGGGGTGCATAC
AAAGCACGAGTTTTTGCCACCGGAACAATTTCTTCG
>Zea_luxurians
TTTTTCGCAACGAACATGCCCAATCCACTACTTTAGGTCCAAAACTCATGTTTGGGGTGG
TTTCGCGCAATTTCGTTGCCGCACGTCACCCATTCCGAAAACGGGTGTCGGGGTGCATAC
AAAGCACGAGTTTTTGCCACCGGAACCATTTCTTCG
>Setaria_italica
TTTTTTCGCAACGAACGCATGCAATCTTCCTAATTATACCCTAGAACATGTTTGGGAGTG
TTTTTGAGCATTCGGTTCCGGCACGAAAAACGATGCAAAACAGGTGCATTTCGGCACCCG
AATGCACTATTTTCGGGTAGCGAAACTCGGGTGGA
>Setaria_viridis
TTTTTTCGCAACGAACGCATGCAATCTTCCTAATTATACCCTAGAACATGTTTGGGAGTG

```
TTTTTGGGCATTCGGTTCCGGCACGAAAAATGATGCAAAACGGGTGCATTTCGGCACCCG
AATGCACTATTGCCGGGTAGCGAAACTCGGGTGGA
>Panicum_virgatum
TTTTTCGCAACGAACGCACCCAATCCACCCCATTGGACCCTAAACTCATGTTTTGGTACG
TTTCATAGTGTTTGGGTGCATTTGGGATCATTTCGTAACTGCATGAAACTCGGTGCAAAA
CGGGGTGAACTGGTGCAATTAATGCAAAAGTTCGTGCCACGAAGTCACGTCGGGA
>Panicum_capillare
TTTTCGCAAGGAACGCACCCGATCCACTCCATGGACCCAAAACTCATGTTTTGGGCGTTT
CGGACCGTTTCGTTACTGCACGAAAGTCGGTGCAAAACGGGCCAACTGGTGCCATTAACG
CACAAGTTCGCTAAACGAAGTTGCGTCGGAA
>Panicum_hallii
TTTTTCGCAACGAACGCACCCGATCCACTCCATTGGACCCAAAACTCATGTTTTGGGGCG
TTTCGGACCGTTTCGTTACTGCACGAAAGTCGATGCAAAACTGGCCGAACTGGTGCCATT
AACGCACAAGTTCGCTAAACAAAGTCGCGTCGGAA
>Oryza_sativa
TTTTTGCCACGAACGCACCCAATACACTCCAATATGTCCAAAAATCATGTTTTGGCCTTT
TTGAACTTTTTCATTCCGGTAAAAAACATCGCACCCACGTGTGCCAATATTGGCATTAAT
TGACAAAAGTTCGCCGCGCGAATCACGAAGTGAG


#################################################################################################
Sorghum

Sorghum_bicolor           GATGCAAGATAGGTGCACGGTTTGCACGGAACGCACCATAGGCTAAGAAACCATTTTGGA 60
Sorghum_propinquum        GATGCAAGATAGGTGCACGGTTTGCATGGAACATACCATATGCTAAGAAATCAATTTGGA 60
Miscanthus_giganteus      GATGCAAGATAGGTGCACGGTTTGCGCCAAACGTACCATAGGCTCAGAAATCATTTTGGA 60
                          *************************    ***  ****** *** ***** ** ******

Sorghum_bicolor           CGCACCCGATGGAACTCCTAGATGAAGTGTGTCAAATGGAAGCTCGGTTCGGTCTGTTTG 120
Sorghum_propinquum        CGCACCCGATGGAACTCCTAGATGACGTGTGTCAAATGGAAGCTCGCTTTGGTCTGTTTG 120
Miscanthus_giganteus      CGCACCCGATGGAACTCCTAGGTGACGTGGGTCATGTGGAATCTCGTTTCGGTCCGTTTG 120
                          ********************* *** *** ****  ***** **** ** **** *****

Sorghum_bicolor           GAGATAGTGTTAATCTT 137
Sorghum_propinquum        GAGACAGTGCTAATCTC 137
Miscanthus_giganteus      GAGATAGTGTTAGTGTT 137
                          **** **** ** * *

--ACTUAL READS---
>Sorghum_bicolor
GATGCAAGATAGGTGCACGGTTTGCACGGAACGCACCATAGGCTAAGAAACCATTTTGGA
CGCACCCGATGGAACTCCTAGATGAAGTGTGTCAAATGGAAGCTCGGTTCGGTCTGTTTG
GAGATAGTGTTAATCTT
>Sorghum_propinquum
GATGCAAGATAGGTGCACGGTTTGCATGGAACATACCATATGCTAAGAAATCAATTTGGA
CGCACCCGATGGAACTCCTAGATGACGTGTGTCAAATGGAAGCTCGCTTTGGTCTGTTTG
GAGACAGTGCTAATCTC
>Miscanthus_giganteus
GATGCAAGATAGGTGCACGGTTTGCGCCAAACGTACCATAGGCTCAGAAATCATTTTGGA
CGCACCCGATGGAACTCCTAGGTGACGTGGGTCATGTGGAATCTCGTTTCGGTCCGTTTG
GAGATAGTGTTAGTGTT

#################################################################################################
Corals

Acropora_millepora      CAAAACCCTAGTGCACGGTACTTTGCACAAAAAGTTGCTTATCTCGAGGAG-ATCG---A 56
Acropora_palmata        CAAAAC----GTGT-CGAT-CTCCTCGAAATAAGCAACTTTTTGTGCAAAGTACCGTGCA 54
                        ******    ***  ** * **   *  ** ***   *** *   *   ** * **   *

Acropora_millepora      CAAG--TTTTGGTG-GTTTTTCAGCAAA-TGCCCTACTTTTTGCAACATTTTTCTAAAAA 112
Acropora_palmata        CTAGGGTTTTGACCCAATTTTGAGCAAAATGTTGCAAAAAGTATGGCATTTGGTGAAAAA 114
                        * **  *****      **** ****** **    *     *    *****    *****

Acropora_millepora      TTGGGT 118
Acropora_palmata        CCAC-- 118

Candidate centromere repeat monomer:
>Acropora_millepora
CAAAACCCTAGTGCACGGTACTTTGCACAAAAAGTTGCTTATCTCGAGGAGATCGACAAG
TTTTGGTGGTTTTTCAGCAAATGCCCTACTTTTTGCAACATTTTTCTAAAAATTGGGT
```

```
>Acropora_palmata
CAAAACGTGTCGATCTCCTCGAAATAAGCAACTTTTTGTGCAAAGTACCGTGCACTAGGG
TTTTGACCCAATTTTGAGCAAAATGTTGCAAAAAGTATGGCATTTGGTGAAAAACCAC

###############################################################################################
Mosquitos
anopheles_gambiae        ATGGCCGTAACAACGATAATAGATGGCAACAAAATTCAACATCAAATTTCAAGGCCATTC 60
anopheles_gambiae_S      ATGGCCGTAACAACGATAATAGATGGCAACAAAATTCAACATCAAATTTCAAGGCCATTC 60
anopheles_gambiae_M      ATGGCCGTAACAACGATAATAGATGGCAACAAAATTCAACATCAAATTTCAAGGCCATTC 60
                         ************************************************************

anopheles_gambiae        AAATAGTGCAAGATGGCTTCATTTGGATGAAAC 93
anopheles_gambiae_S      AAATAGTGCAAGATGGCTTCATTTGGATGAAAC 93
anopheles_gambiae_M      AAATAGTGCAAGATGGCTTCATTTGGATGAAAC 93
                         *********************************

Candidate centromere repeat monomer:
>anopheles_gambiae
ATGGCCGTAACAACGATAATAGATGGCAACAAAATTCAACATCAAATTTCAAGGCCATTC
AAATAGTGCAAGATGGCTTCATTTGGATGAAAC
>anopheles_gambiae_M
ATGGCCGTAACAACGATAATAGATGGCAACAAAATTCAACATCAAATTTCAAGGCCATTC
AAATAGTGCAAGATGGCTTCATTTGGATGAAAC
>anopheles_gambiae_S
ATGGCCGTAACAACGATAATAGATGGCAACAAAATTCAACATCAAATTTCAAGGCCATTC
AAATAGTGCAAGATGGCTTCATTTGGATGAAAC

###############################################################################################
Drosophila 1

Drosophila_erecta      TAATTCCCAACTTGTTATGGCTTATATTTCATTATACGTTCCCTCTAACAGCCTATAAAG 60
Drosophila_yakuba      TAATTCCCAACTTGTTCTGG-TTATTTTTCCTTATATGTACTCTCTACCTGCCCATAAAC 59
                       **************** *** **** **** ***** ** * ***** * *** *****

Drosophila_erecta      TAGTGGACAGGAAG--TTCCGTGAAT-TTAGT-TAATAAATGTG--TTCATG-------- 106
Drosophila_yakuba      TAGAGAATGTAATGGCCTCTATAAACGTTGATGCAGGCGATGGGGATTCAGGAGCTGCGC 119
                       *** * *    * *   **  * **  **  *  *    *** *  **** *

Drosophila_erecta      ----------TTTGTGTTTG---CGC-----------ACGAAA------------AGTGG 130
Drosophila_yakuba      GGGTGTAAAATCTGCATTCAGGACGCCGTGAACCAGGACCAAACCAAGCCAGGTTAGTAG 179
                                 * **  **     ***           ** ***            *** *

Drosophila_erecta      T--------------------------------TTCAT---GTGGTGCGCAGATAAA--C 153
Drosophila_yakuba      TGGAATGAACTATATTTTTTAAAACTATATGTGTTCATTGAATAATACACAATTAAAGTC 239
                       *                                *****    *  * * **  ****  *

Drosophila_erecta      AATCTACATCCAG----AAAGAA---GAA-------------------AATATAA----- 182
Drosophila_yakuba      CATCGACATTTAATATTAAAAAAACTGAATTACTGTCAGCTTACACCAAATATGACATGT 299
                        *** ****  *     *** **   ***                   ***** *

Drosophila_erecta      ------ACTCT--AAACTCTAGAC--------CAAGTCA---TCGG-----TAATTGTAA 218
Drosophila_yakuba      CAACTGATTCCCGAAAATATACACAGTCAAAACAAGTTAAGTTTGGCCACGTAATTGTTA 359
                             * **   *** * ** **        ***** *   * **     ******* *

Drosophila_erecta      --------TTAAAAACTGGTGCACATAGTGTTCAAAAA 248
Drosophila_yakuba      ATGATTGTTTGAAAAGTA-TATACATGATGCTTGTAAA 396
                               ** **** *  *  ****  ** *   ***

Candidate centromere repeat monomer:
>Drosophila_erecta
TAATTCCCAACTTGTTATGGCTTATATTTCATTATACGTTCCCTCTAACAGCCTATAAAG
TAGTGGACAGGAAGTTCCGTGAATTTAGTTAATAAATGTGTTCATGTTTGTGTTTGCGCA
CGAAAAGTGGTTTCATGTGGTGCGCAGATAAACAATCTACATCCAGAAAGAAGAAAATAT
AAACTCTAAACTCTAGACCAAGTCATCGGTAATTGTAATTAAAAACTGGTGCACATAGTG
TTCAAAAA
>Drosophila_yakuba
TAATTCCCAACTTGTTCTGGTTATTTTTCCTTATATGTACTCTCTACCTGCCCATAAACT
AGAGAATGTAATGGCCTCTATAAACGTTGATGCAGGCGATGGGGATTCAGGAGCTGCGCG
GGTGTAAAATCTGCATTCAGGACGCCGTGAACCAGGACCAAACCAAGCCAGGTTAGTAGT
GGAATGAACTATATTTTTTAAAACTATATGTGTTCATTGAATAATACACAATTAAAGTCC
ATCGACATTTAATATTAAAAAAACTGAATTACTGTCAGCTTACACCAAATATGACATGTC
```

```
AACTGATTCCCGAAAATATACACAGTCAAAACAAGTTAAGTTTGGCCACGTAATTGTTAA
TGATTGTTTGAAAAGTATATACATGATGCTTGTAAA

###############################################################################################
Drosophila 2

Drosophila_sechellia      TTTGTGCAAAATTTTTGGATTTTTCGATTTTAGATACCAGGCGATGATAATCAGTAGCGG 60
Drosophila_simulans       TTTGTGCAAAATTTTTGGATCTTTCGATTTTAGATACCAGGCGATGATAATCAGTAGCGG 60
                          ******************** ***************************************

Drosophila_sechellia      GTGTCTACTGAAAACCAACTAATCGTTGGTCACCTTCTGGAATTCTTGTTCGCCTGGTAA 120
Drosophila_simulans       GTGTCTACAGAAAACCA-CTTATCGTTGGTCACCTTCTGGAATTCTTGTTCGCCTGGTAG 119
                          ******** ******** ** ************************************** 

Drosophila_sechellia      TTTAAACCGAAAAATCTCTCAATTTG--CAACAAAATGCGTATT 162
Drosophila_simulans       TTTAAACCGAAAAATCTCTCAATTTGGCCTACAAAATGCGCATT 163
                          **************************  * ********** ***

Candidate centromere repeat monomer:
>Drosophila_sechellia
TTTGTGCAAAATTTTTGGATTTTTCGATTTTAGATACCAGGCGATGATAATCAGTAGCGG
GTGTCTACTGAAAACCAACTAATCGTTGGTCACCTTCTGGAATTCTTGTTCGCCTGGTAA
TTTAAACCGAAAAATCTCTCAATTTGCAACAAAATGCGTATT
>Drosophila_simulans
TTTGTGCAAAATTTTTGGATCTTTCGATTTTAGATACCAGGCGATGATAATCAGTAGCGG
GTGTCTACAGAAAACCACTTATCGTTGGTCACCTTCTGGAATTCTTGTTCGCCTGGTAGT
TTAAACCGAAAAATCTCTCAATTTGGCCTACAAAATGCGCATT

###############################################################################################
Drosophila 3

Drosophila_ficusphila      CCATAACTTTTGAAATTTTTACCCGATTTAAAAGTGGAATACCTCTCTGA 50
Drosophila_rhopaloa        CCATAACTTTACCAAACTTAAGCCGATTCCAAAGTGGCATACCTCTAAAG 50
                           **********   **  ** * ******  ******* ********    

Drosophila_ficusphila      ATTTGTTATTAAAATATCTATCTAGCTGCATTATTGGTTTATTTTCGAAT 100
Drosophila_rhopaloa        ACTTCTGATTCGATTCTCTAAAAATCTGCATT-----CAAATTTTTCAAT 95
                           * ** * ***  * * ****   * *******        *****  ***

Drosophila_ficusphila      TT----TTGGTCAATATTTTGTAATTTTTTATGACCCCCGACCTGTCAAA 146
Drosophila_rhopaloa        TTAACGTTGATCAA-ATTTT-TGACCCATTTTCATGTCAGTTATTTCTAA 143
                           **    *** **** ***** * *    ** * *   * *   * ** **

Drosophila_ficusphila      ATTTGCAAAAAATGGGTTTGCAGAAAAGTGACCAGATCCCAGCACTG--C 194
Drosophila_rhopaloa        -TTTCCGA-----GGACTTGTAACAGAGTGACT------CAAAATTGGAC 181
                            *** * *     **  *** *  * ******       **  * **  *

Drosophila_ficusphila      TTAGC---- 199
Drosophila_rhopaloa        TTAGAGAGT 190
                           ****

Candidate centromere repeat monomer:
>Drosophila_ficusphila
CCATAACTTTTGAAATTTTTACCCGATTTAAAAGTGGAATACCTCTCTGAATTTGTTATT
AAAATATCTATCTAGCTGCATTATTGGTTTATTTTCGAATTTTTGGTCAATATTTTGTAA
TTTTTTATGACCCCCGACCTGTCAAAATTTGCAAAAAATGGGTTTGCAGAAAAGTGACCA
GATCCCAGCACTGCTTAGC
>Drosophila_rhopaloa
CCATAACTTTACCAAACTTAAGCCGATTCCAAAGTGGCATACCTCTAAAGACTTCTGATT
CGATTCTCTAAAAATCTGCATTCAAATTTTTCAATTTAACGTTGATCAAATTTTTGACCC
ATTTTCATGTCAGTTATTTCTAATTTCCGAGGACTTGTAACAGAGTGACTCAAAATTGGA
CTTAGAGAGT

###############################################################################################
Drosophila 4

Drosophila_virilis      ACTATATCATATAGCTGCCATAGGAACGATCGGTCGAAAATTAAGTTTTTGTATGAAA-- 58
Drosophila_elegans      ACTATATCATATAGCTCCCATAGGAACAA----TCGAAAAATAAAT----GAAAAAAATT 52
                        **************** ********** *    ******* *** *    * *  ***  

Drosophila_virilis      --AACATTT-TGTTTTTCAAGATATCTTGACCAAAC--TCGGCATTTATTAGTTTTACTA 113
```

```
Drosophila_elegans      ATAACTTTTCTGTTTTT---AACTTTTTGTTTAGTTCTTCGACATATAGCAATGGTTAAA 109
                          *** *** *******    *  * ***   *     *** *** **  * *  *   *

Drosophila_virilis      TACTCCTCATATATATGCAAAATCCTATTAAGATCGGACC 153
Drosophila_elegans      TATTTCAGAATTACGGTTTAAATTTCATCAAAATCGGACG 149
                        ** * *  *  **      ****   ** ** *******
Candidate centromere repeat monomer:
>Drosophila_virilis
ACTATATCATATAGCTGCCATAGGAACGATCGGTCGAAAATTAAGTTTTTGTATGAAAAA
CATTTTGTTTTTCAAGATATCTTGACCAAACTCGGCATTTATTAGTTTTACTATACTCCT
CATATATATGCAAAATCCTATTAAGATCGGACC
>Drosophila_elegans
ACTATATCATATAGCTCCCATAGGAACAATCGAAAAATAAATGAAAAAAATTATAACTTT
TCTGTTTTTAACTTTTTGTTTAGTTCTTCGACATATAGCAATGGTTAAATATTTCAGAAT
TACGGTTTAAATTTCATCAAAATCGGACG

##################################################################################################
Wasps

Nasonia_giraulti         CGCTTTGGTTTTAGATTTTATACTCGCTTCGCTCGCCTTCCTTCGATGTGCCGAGGTGTT 60
Nasonia_longicornis      CGCTTTGGTTTTAGATTTTATACTCGCTTCGCTCGCCTTCCTTCGATGTGCCGAGGTGTT 60
Nasonia_vitripennis      CGCTTTGGTTTTTGATTT-ATACTCGCTTTGCTCGCCTTCCTCCGATGTGCCGAGGTGTT 59
                         ************ ***** ********** ************ *****************

Nasonia_giraulti         TTTTAAATAAATTATTGAGCTCGGGGAGCGTGGTGTAGTTGGGTTTAAG 109
Nasonia_longicornis      GATTAAATAAATTATTGAGCTCGGGGAGCGTGGTGTAGTTGGGTTTAAG 109
Nasonia_vitripennis      GTTTAAATAAATTATTGAGCTCGGGGAGCGTGGTGTAGTTGGGTTTAAG 108
                           ***********************************************

Candidate centromere repeat monomer:
>Nasonia_giraulti
CGCTTTGGTTTTAGATTTTATACTCGCTTCGCTCGCCTTCCTTCGATGTGCCGAGGTGTT
TTTTAAATAAATTATTGAGCTCGGGGAGCGTGGTGTAGTTGGGTTTAAG
>Nasonia_longicornis
CGCTTTGGTTTTAGATTTTATACTCGCTTCGCTCGCCTTCCTTCGATGTGCCGAGGTGTT
GATTAAATAAATTATTGAGCTCGGGGAGCGTGGTGTAGTTGGGTTTAAG
>Nasonia_vitripennis
CGCTTTGGTTTTTGATTTATACTCGCTTTGCTCGCCTTCCTCCGATGTGCCGAGGTGTTG
TTTAAATAAATTATTGAGCTCGGGGAGCGTGGTGTAGTTGGGTTTAAG

##################################################################################################
Zebrafish

Danio_rerio               ATTTTCACTTGCAACTTGCTTTAA----CATGTTTAACAGTGCATGAATTGTT---GTCT 53
Danio_nigrofasciatus      ATTTTCAGCTTTCTCTTGCTCTGAAAAGCGTGTTTAGAGTTGCCTGTACTGTTAGTGATC 60
Danio_albolineatus        ATTTTCACTTGCAAGTTGTTAAAA----CATGCTTAATACAGCATGCACTGTTG--TTTT 54
                          *******  *     *** *   *    * ** ***     ** ** * ****

Danio_rerio               AAAACAAAAAAGAATGATGCATTTTATGGCTGGATC--GTTTT-ATCACTTTCTGAATTG 110
Danio_nigrofasciatus      AAAACAAAAA-GAATGATGGTTTTCATCATTTCTTTTTGTTTTGATCACTCTCTGTTCTG 119
Danio_albolineatus        TAAACTTAAA-GAATGATGATTTTCACTGTTTTAAGCAAATTCTGTCACTTGCTCTTTTT 113
                           ****  *** ********  *** *    *         **   *****  **    *

Danio_rerio               G-ACGTTGAAAATGAGCTTATCTCAG---CCAGCAGAGAGGTCA----AATCACAGTAAT 162
Danio_nigrofasciatus      G-GCCCTCTAAACG----TGTTTTAA---TAAGTTGCAAGTGAC----AATCA--GCAGA 165
Danio_albolineatus        GCACTTACCAAGC-------TCTGAAAAGCAAGTTGCAGAATTCTAGGAATTG--ACAAT 164
                          *  *     **         * * *      **  *            ***      *

Danio_rerio               GCAAACTCAGTCAGAGACATAG---- 184
Danio_nigrofasciatus      TCTGGCTGAGTTTGCCATACTGTTT- 190
Danio_albolineatus        TATGAAACA--TTATCCTTTTGCTTA 188
                                  *            *

Candidate centromere repeat monomer:
>danio_rerio
TAAAACGATCCAGCCATAAAATGCATCATTCTTTTTTGTTTTAGACAACAATTCATGCAC
TGTTAAACATGTTAAAGCAAGTTGCAAGTGAAAATCTATGTCTCTGACTGAGTTTGCATT
ACTGTGATTTGACCTCTCTGCTGGCTGAGATAAGCTCATTTTCAACGTCCAATTCAGAAA
GTGA
>Danio_albolineatus
TTTAAGCAAATTCTGTCACTTGCTCTTTTTGCACTTACCAAGCTCTGAAAAGCAAGTTGC
```

```
AGAATTCTAGGAATTGACAATTATGAAACATTATCCTTTTGCTTAATTTTCACTTGCAAG
TTGTTAAAACATGCTTAATACAGCATGCACTGTTGTTTTTAAACTTAAAGAATGATGATT
TTCACTGT
>Danio_nigrofasciatus
GTTTAGAGGGCCCAGAACAGAGAGTGATCAAAACAAAAAGAAATGATGAAAACCATCATT
CTTTTTGTTTTGATCACTAACAGTACAGGCAACTCTAAACACGCTTTTCAGAGCAAGAGA
AAGCTGAAAATAAACAGTATGGCAAACTCAGCCAGATCTGCTGATTGTCACTTGCAACTT
ATTAAAACAC

#################################################################################################
Cichlids

Pundamilia_nyererei            GTAACTTTTGATAGAAGACTCAGACAAACATGTTTATGGCTTTATCTTAT 50
melanochromis_auratus          GTAACTTTTGATAGTAGACTCAGACAGACATGTTCATGGCTTTATCTTAT 50
labeotropheus_fuelleborni      GTAACTTTTGATAGAAGACTCAGAAACACATGTTTATGGCTTTATCTTAT 50
metriaclima_zebra              GTAACTTTTGATAGAAGACTCAGATACACATGTTTATGGCTTTATCTTAT 50
rhamphochromis_esox            GTAACTTTTGATAGAAGACTCAGACAAACATGTTTATGGCTTTATCTTAT 50
oreochromis_niloticus          GTAACTTTTGATAGGAGACTCGGACACACATATTTCAGGCTTGGCCTTAT 50
                               ************** ****** ** * **** **   *****   *****

Pundamilia_nyererei            AGAACTCAATATCCCCGTGCTGGGCAAACAGGTTTTGCAGCCGTTTGAGC 100
melanochromis_auratus          AGAACTCAATATCCCCGTGCTGGCCGAACAGGTTTTGCAGCCGTTTGAGC 100
labeotropheus_fuelleborni      AGAACTCAATATCCC-GTGCTGGGCAAACAGGTTTTGCAGCCGTTTGAGC 99
metriaclima_zebra              AAAACTCAAGATCTCCGTGCTGGGAAAACAGGTTTTGCAGCCGTTTGAGC 100
rhamphochromis_esox            AGAACTCAATGTCCCCGTGCTGGGAAAACAGGATTTGCAGCTGTTTGAGC 100
oreochromis_niloticus          AGAATTCAGCATTTCCATGCTGGGGAAATAGGTTTTGCCACTGTTTGAGC 100
                               * ** ***   *  *  ******   ** *** *****  * ********

Pundamilia_nyererei            TAAGATTTTAAAATTATTCACATAATGAAAACCTATACTTTGTTTCAGGC 150
melanochromis_auratus          TAAGATTTTAAAATTATTCACATAATGAAAACCTATACTTTGTCTCGGGC 150
labeotropheus_fuelleborni      TAAGATTTTAAAATTATTCACATAATGAAAACCTATACTTTGTTTCAGGC 149
metriaclima_zebra              TAAGATTTTAAAATTATTCACATAATGAAAACCTATACTTTGTCTCGGGC 150
rhamphochromis_esox            TAAGATTTTCAAGTTATACACATAATGAAAACCTATACTTTGTTTCGGTC 150
oreochromis_niloticus          TAAGATTTTCA-GTTATTGACTAAATGAAAACC-ATAATGTGTTTCAGGC 148
                               ********* *  ****  **  ********** *** * *** ** * *

Pundamilia_nyererei            GAGTTTCCCATTCAAATGCATGT--CAGTGAGAAACGCACTGTCTTGGCG 198
melanochromis_auratus          GAGTTTCCCATTCAAATGCATGTAACAGTGAGAAACGCACTGTCTTGGCG 200
labeotropheus_fuelleborni      GAGTTTCCCATTCAAATGCATGTAACAGTGAGAAACGCATTGTCTTGGCG 199
metriaclima_zebra              GAGTTCCCCATTCAAATGCATGTAACAGTGAGAAACGCATTGTCTTGGCG 200
rhamphochromis_esox            GAGTTCCCCATTCAAATGCATGTAACAGTGAGAAACGCACTGTCTTGGCG 200
oreochromis_niloticus          GAG-----------------------------AAACGCACTGTCTCGCCG 169
                               ***                             ******* ***** * **


Pundamilia_nyererei            AAATAAAGCGTTTTTGTACAACTTCATATAAATCGCT 235
melanochromis_auratus          AAATAAAGCATTTTTGTACAACTTCATATAAATCGCT 237
labeotropheus_fuelleborni      AAATAAAGCGTTTTTGAACAACTTCATATAAATCGCT 236
metriaclima_zebra              AAAGAAAGCGTTTTTGTACAACTTCATATAAATAGCT 237
rhamphochromis_esox            AAAGAAAGCGTTTTTGTACAACTTCATAAAAATCGCT 237
oreochromis_niloticus          AAATAAGGCGATTTTCACCAAGTCCATAAAGACAGCT 206
                               *** ** **  ****   *** * **** * *  ***

Candidate centromere repeat monomer:
>pundamilia_nyererei
GTAACTTTTGATAGAAGACTCAGACAAACATGTTTATGGCTTTATCTTATAGAACTCAAT
ATCCCCGTGCTGGGCAAACAGGTTTTGCAGCCGTTTGAGCTAAGATTTTAAAATTATTCA
CATAATGAAAACCTATACTTTGTTTCAGGCGAGTTTCCCATTCAAATGCATGTCAGTGAG
AAACGCACTGTCTTGGCGAAATAAAGCGTTTTTGTACAACTTCATATAAATCGCT
>melanochromis_auratus
GTAACTTTTGATAGTAGACTCAGACAGACATGTTCATGGCTTTATCTTATAGAACTCAAT
ATCCCCGTGCTGGCCGAACAGGTTTTGCAGCCGTTTGAGCTAAGATTTTAAAATTATTCA
CATAATGAAAACCTATACTTTGTCTCGGGCGAGTTTCCCATTCAAATGCATGTAACAGTG
AGAAACGCACTGTCTTGGCGAAATAAAGCATTTTTGTACAACTTCATATAAATCGCT
>labeotropheus_fuelleborni
GTAACTTTTGATAGAAGACTCAGAAACACATGTTTATGGCTTTATCTTATAGAACTCAAT
ATCCCGTGCTGGGCAAACAGGTTTTGCAGCCGTTTGAGCTAAGATTTTAAAATTATTCAC
ATAATGAAAACCTATACTTTGTTTCAGGCGAGTTTCCCATTCAAATGCATGTAACAGTGA
GAAACGCATTGTCTTGGCGAAATAAAGCGTTTTTGAACAACTTCATATAAATCGCT
>metriaclima_zebra
GTAACTTTTGATAGAAGACTCAGATACACATGTTTATGGCTTTATCTTATAAAACTCAAG
```

```
ATCTCCGTGCTGGGAAAACAGGTTTTGCAGCCGTTTGAGCTAAGATTTTAAAATTATTCA
CATAATGAAAACCTATACTTTGTCTCGGGCGAGTTCCCCATTCAAATGCATGTAACAGTG
AGAAACGCATTGTCTTGGCGAAAGAAAGCGTTTTTGTACAACTTCATATAAATAGCT
>rhamphochromis_esox
GTAACTTTTGATAGAAGACTCAGACAAACATGTTTATGGCTTTATCTTATAGAACTCAAT
GTCCCCGTGCTGGGAAAACAGGATTTGCAGCTGTTTGAGCTAAGATTTTCAAGTTATACA
CATAATGAAAACCTATACTTTGTTTCGGTCGAGTTCCCCATTCAAATGCATGTAACAGTG
AGAAACGCACTGTCTTGGCGAAAGAAAGCGTTTTTGTACAACTTCATAAAAATCGCT
>oreochromis_niloticus
GTAACTTTTGATAGGAGACTCGGACACACATATTTCAGGCTTGGCCTTATAGAATTCAGC
ATTTCCATGCTGGGGAAATAGGTTTTGCCACTGTTTGAGCTAAGATTTTCAGTTATTGAC
TAAATGAAAACCATAATGTGTTTCAGGCGAGAAACGCACTGTCTCGCCGAAATAAGGCGA
TTTTCACCAAGTCCATAAAGACAGCT

#################################################################################################
Cats

Felis_catus           GCACTGGGTTCACTGGAGGCTGCAGTGCCGCGG 33
Felis_silvestris      GCACTGGGTTCACTGGAGGCTGCAGTGCCGCGG 33
                      *********************************

Candidate centromere repeat monomer:
>Felis_catus
GCACTGGGTTCACTGGAGGCTGCAGTGCCGCGG
>Felis_silvestris
GCACTGGGTTCACTGGAGGCTGCAGTGCCGCGG

#################################################################################################
Bovine

Bison_bison             AATGGAAGATTGGACTT-CCCTGGGCCAAACACAAGAGG--CATC-CTGAATTCCCCGT- 55
Bos_taurus_taurus       AATGGAAGATTGGACTT-CC-TGGGC--AACACAAGAAGGCCATCACTGATTTCCCCGTT 56
Bos_grunniens           -ATGGAAGATTGGACTT-CCCTGGGCC-AACACAAGAGG--CATC-CTGAATTCCCCGT- 53
Bos_taurus_indicus      AATGGAAGATTGAACTTGCCCTGGGCC-AAC-CAAAAGG--CATC-CTGACTTCCCCGT- 54
                         *********** **** ** *****  *** *** * *  **** **** ********

Bison_bison             CGTAATTCGAGAATCC--TGCC-GACACTCGAGAAAATCCACGTG--TTCCCCCGTCATC 110
Bos_taurus_taurus       CGTAACTCGAGATTCCGCCGCC-AA-ACTCGAGACA-CCAACGTGGATTCCCCCGTCATC 113
Bos_grunniens           CGTAACTCGAGAATCC--CGCC-GCAACTCGAGAAAAACCACGTGG-TTCCCCCGTCATC 109
Bos_taurus_indicus      CGTAACTC-AGAATCC-CCGCCCGTAACTCGAGAAAAACCACGTGG-CTCCCCGTCCATC 111
                        ***** ** *** ***   ***    ******** *  * *****   *****   ****

Bison_bison             -GCA--AGAT-GAAGCCCTTTCCCGCTACAGT--GTCT--CAGGAGAAGTCCCACGTTAG 162
Bos_taurus_taurus       -GCACAAGATTGAAGCCCTTTCC-GCTACAGC--GTCT--CAGGAGAAGTCCCACGTTAG 167
Bos_grunniens           -GCA--AGAT-GAAGCCCTTTCCCGCTACAGC--GTCT--CAGGAGAAGTCCCACGTTAG 161
Bos_taurus_indicus      CGCA--AGAT-GAAGGCCCTTCCCGCAACAGCCGGCCCTCCAGGAGAAGTCCCACGT-AG 167
                         ***  **** **** ** **** ** ****   * *   ***************** **

Bison_bison             GTATTGGAGGTCGAAACGGTACTTGGC-ACC-TTGATG-CGACC--ACAAAGTGCCCCGA 217
Bos_taurus_taurus       GAATTGAGCGGTGGAACGGTACTTGGC-ACCCTTGACGGCGGCCCCACAAAGTTCCCCGG 226
Bos_grunniens           GAATTGGAGGTCGAAACGGTACTTGGCCACCCTTGATG-CGACCC-ACAAAGTTCCC-GA 218
Bos_taurus_indicus      G-ATTGGAGGTCGAAAGGGCACTTGGC-GCCCTTGATG-CGACCC-ACAAAGTTCCCCGA 223
                        * ****   *  * ** ** *******  ** **** * ** **  ******* *** *

Bison_bison             CATCCCGGTCCTCCCTCGAGAGGAACACC-GAAGTTTTCCGGC-ACCACTTCCTCTGAGC 275
Bos_taurus_taurus       CATCCCG--TCTCCCTGGAGAGGAACACC-GAG--TTTCCGGC-ACCACTTCCTCTGAGC 280
Bos_grunniens           CATCCCGGTCTCCCTCGGAGAGGAACCCCTGAGGTTTTCCGGCCACCACTTCCTCTGAGC 278
Bos_taurus_indicus      AATCCCCGGTCTCCCTCGAGAGGAACACT-GAGGCTTTC--GC-ACCCCCTCCTCTGACC 279
                         *****      **   ********* *  **   ****  ** *** * ******** *

Bison_bison             CCCTTCTACCC-TCCTGA-TCTGGACAGG-AGGGTCGACTCCCCTGCTTTGTCTGGAAGG 332
Bos_taurus_taurus       CCCTTCTACCC-TCCTGA-TCTCGACAGGGAGGGTCGACTCCCCTGCTTTGTCTGGAAGG 338
Bos_grunniens           CCCTTCTACCC-TCCTGA-TCTGGACAGG-AGGGTCGACTCCCCTGCTTTTTCTGGAAGG 335
Bos_taurus_indicus      CTTTTCTCCCCCTCCTGAATCTGGACAGG--AGGTCGACTCCCCTGCTT-GTCTGGAAGG 336
                        *  **** *** ****** *** ******   *****************  *********

Bison_bison             GGTTCCCGACCTTCCGGTCGCACCTCAGGATGAGGCCGGGTCTCA-CGACGAC-ATTTCA 390
Bos_taurus_taurus       GGTTCCCGACCTTCCGGTCGCACCTCAGGATGAGGCCGGG-CTCA-CGACGAC-ATTCCA 395
Bos_grunniens           GGTTCCCGACCTTCCGGTCGCACCTCAGGATGAGGCCGGT-CTCA-CGACGAC-ATTCCA 392
Bos_taurus_indicus      GGTTCC-GACCTTCCGTCCACCTCCAGGAATGAGGCCGGT-CTCAACGAAGACCATTCCA 394
                        ****** *********  * *  *   * **********  **** *** *** *** **
```

```
Bison_bison             G-ACGTGGCCT--CGTGGGTGGTTCC-ACATTCCGAAGGAC-CCCGATTTCCCGGTCCCC 445
Bos_taurus_taurus       G-ACGTGGCCT--CGTGGGTGCTTCC-ACATTGCGAAGCAC-CCCGATTTCCCGGTCCCC 450
Bos_grunniens           GGACGTGGCCT--CGTGGGTGGTTCC-ACATTGCGTAAGACACCCGATTTCCCGGTCCCC 449
Bos_taurus_indicus      --ACGTGCCCCTCCGTGGGTGGTTCCCACATT-CGTAGGAC-CCCGATTCCC--GTCCCC 448
                          ***** **   ******** **** ***** ** *  ** ******* **  ******

Bison_bison             TCTTG-ATAAGAACCCGATGCCCGGACACCTCTT-CGAACCTCCACCCTGTGAATGAAGT 503
Bos_taurus_taurus       TCTTG-GTAAGAACCCGATGCCCGGACACCTCT--CGAAC-TCCACCCTGTGAATGAGGT 506
Bos_grunniens           TCTTG-ATAAGAACCCGATGCCCGGACACCTCTTCCGAAC-TCCACCCTGTGAATGAAGT 507
Bos_taurus_indicus      TCCTGGATAAGAACCCGATGCCGGACCACCTCTC-CGAACTCCAACCCTG-GAATGAAGT 506
                        ** **  *************** *  *******  *****  * ****** ****** **

Bison_bison             CAACACGAAAGGGCAGTGACTCGCCCGTGCATCGTCGGGAAAAAACCCCCAGGTTCCAAA 563
Bos_taurus_taurus       CAACACGAAGGGGCAGTG-CCCGCCCGTGCATCGTCCGGAAAGAACCCCCAGGTTCCAAA 565
Bos_grunniens           CAACACGAAGGGGCAGTGACTCGCCCGTGCATCGTTCGGGGAAAACCCCCAGGTTCCAAA 567
Bos_taurus_indicus      CAACACGAAGGGCCAAT---TTTTCCGTGCATCGTTCAGAAAAA---CCCAG-TTCCAAA 559
                        ********* ** ** *       ***********   *  * *   ***** *******

Bison_bison             TACCGCTCGACAAGT-GGCCTGTCT-CCCCGGGAAACACCTCGAGAGGCAAGCGGAGTTC 621
Bos_taurus_taurus       TACAGCTCGACAAGT-GGCCTCTCT-CCCCGGGGA-CACCTCGAGAGGCAAGCGGAGTTC 622
Bos_grunniens           TACAGCTCGACAATG-GGCCTCTCT-CCCCGGGGA-CACCTCGAGAGGCAAGCGGAGTTC 624
Bos_taurus_indicus      TACAGCTCGACAAGCCGGCTCTTCTTCCCCCGGGACCATCTCGAGATGCAAGCGGAGTTC 619
                        *** *********   ***   *** **** ** * ** ******* *************

Bison_bison             CATGCCTCA--CCCAAGACGAGG-CCTGACTCTCCCTGTCCCAAGTCTGCAGGGACCTTG 678
Bos_taurus_taurus       CATGCCTCAA-CCCAAGACGAGGACCTGACTCTCC-TGTCCCCAGTCTGCAGGGACCCTG 680
Bos_grunniens           CATGCCTCAA-CCCAAGACGAGG-CCTGACTCTCC-TGTCCCAAGTCTGCAGGGACCCTG 681
Bos_taurus_indicus      CATGCCTCAAACCAAAGACAG----CCGACTCTCC-TGTCCCAGTCCTGCAGA---CCTG 671
                        *********  ** *****      * ******** ******    ******    * **

Bison_bison             CGAT-CAGAGTCTGAAGTCAGAGGAACCCTGAGGTTCC-TGCCTCAACTGGAGATG-AGG 735
Bos_taurus_taurus       CGATTCGGAGTCTGAAATCAGAGGAACCCTGAGGTTCC-TGCCTCAACTGGAGATGGAGG 739
Bos_grunniens           CGAT-CGG--TCTGAAATCAGAGGAACCCTGAGGTTCC-TGCCTCAACTGGAGATG-AGG 736
Bos_taurus_indicus      CGATCGGAAGTCTGAA-TCA-AGGTACCCTGCGGTTCCGCCCCTCAACTGGAGATG-AGG 728
                        ****      ****** *** *** ****** ******   *************** ***

Bison_bison             CCCTCTTCCAATGCACCAAA-CCCAGTGGGAGTGCCGAGAAGCCC-TCCCA-CCTCCAGT 792
Bos_taurus_taurus       CCCTCTTCCAATGCACCAAAACCCCGTGG-AGTCCCGAGAGGCCCCTCCCA-CCTCCAGT 797
Bos_grunniens           CCCTCTTCCAATGCACCAA--CCCCATGG-AGTCCCGAGAGGCCCCTCCCAACCTCCAGT 793
Bos_taurus_indicus      CC-TCTTCCAATGCACCAAAACCCAGTGG-GGTCCCGAGAGGCCCTTCCCA-CCTCCAGG 785
                        ** ****************  ***  ***  ** ****** **** ***** *******

Bison_bison             TTTCCCTGACTTCTCAGAGCC-ACCATGAGAAGCCCCCTGAGGTCACCTGCA-CAAGTCA 850
Bos_taurus_taurus       TC-CGCTGA-TTCTCAGAGCCCACCATGAGAAGCCCCCTGAGGTCACCTGCA-CAAGTCG 854
Bos_grunniens           TT-CCCTGACTTCTCAGAGCC-ACCATGAGAAGCCCCCTGAGGTCACCTGCA-CAAGTCG 850
Bos_taurus_indicus      TT-CCCTG-CTTCTCAGAGCC-ACCATGGAGAAGCCCCTGAGGTCACCTGCAACAAGTCG 842
                        *  * ***  *********** ******   *  ****************** ******

Bison_bison             AGGGAAGCCA--GGTTTTCTGCCTCAACCCGAGAAAGACC---TCGAGAGACCTTCTTCA 905
Bos_taurus_taurus       AGGGAAGCCAAGGGTTCCCTGCCTCAACCCGAGAAAGACC---TCGAGAGACCTTCTTCA 911
Bos_grunniens           AGGGAA-CCA-GTGTTTCCTGCCTCAACC-GAGAAAGACCCCCTCGAGAGACCTTCTTCA 907
Bos_taurus_indicus      AGG-AACCCA-GGGTTTCCTGCCTCAACC--GAAAAGACC---TCGAGAGACCTTCT-CA 894
                        *** ** ***   ***  ***********    *******   ************** **

Bison_bison             ACA-CGTCTC-GAG-CCAGATTCCCCTAACAGTGACTC-GAGA-GCAATGACGCGCTCCC 960
Bos_taurus_taurus       ACATCGTCTC-GAGGCCAGGTTCCC-TACCAG-GACTC-GAGG-GCAATGACGCGCTCCC 966
Bos_grunniens           ACA-CGTCTC-GAGGCCAGATTCCCCTACCAT-GACTCCGAGGAGCAATGACGCGCTCCC 964
Bos_taurus_indicus      ACA-CGTCTCTGAGGCCAA--TCCC-TAACAT-GGCTCGGGAATCCAGTGACGCGCTCCC 949
                        *** ****** *** ***   **** ** **  * *** *     ** ************

Bison_bison             CCTCGCCATTGCGCA-TGGAGACCCCGACTTCCCTGGCGCCC-ACGAGAG--CTCACTGA 1016
Bos_taurus_taurus       CCTCGCCACT-CGCG-TGGAGACCC-GACTTCCCTGGCGCCCCACGAGAGG-CTCACTGA 1022
Bos_grunniens           CCTCGCCACT-TGCA-TGGAGACCC-GACTTCCCTGG-GCCCCACGAGAGG-CTCACTGA 1019
Bos_taurus_indicus      CCCCACCACTTCGCACTGGAGAACCCGACTTCCCTGGCACCCCACCAGAGGCCTCACTGA 1009
                        ** * *** *  **  ****** ** ***********  *** ** ****  ********
```

```
Bison_bison             CCTCGCCGTCGTACACT--AGGA-AAAACCGCACACTGTGGC-GCCAGCTCGA-GAACAA 1071
Bos_taurus_taurus       CCTCGCCGTCGCACCTC--GTGA-GAAACCGCACCCTGGGGCCGCCGGCTCGA-GAACCA 1078
Bos_grunniens           CCTCGCCGTCGTACCTC--GTGA-GAAACCGCACCCTGGGGCCGCC-GCTCGA-GAACAA 1074
Bos_taurus_indicus      CCTCGCCG-CGTACCTCCAGTGAAGAAAACAC-CACCGGGGCCGCC-GCTCGAAGAACAA 1066
                        ******** ** **       **  *** * * * * * *** *** ****** **** *

Bison_bison             --CCCTGAGCCCTCC-CCATCATCGCG-AGTTGAGGG-CCTTCGTCTCCTGTATGG-CCT 1125
Bos_taurus_taurus       --CCC-GAGACTCCC-CCGTCATCGCGGAGATGAGGG-CCTTCGTCTCCTGCATGGGCCT 1133
Bos_grunniens           --CCCCGAGACTCCC-CCGTCATCGCG-AGATGAGGGGCCTTCGTCTCCTGCATGG-CCT 1129
Bos_taurus_indicus      ACCCCCGAGAATTCCACCCTCATCGAG-AGATGAGGGCCCTCCGCCCCCT---CAGGCCT 1122
                          *** ***    ** ** ****** * ** ****** *** ** * ***     * ***

Bison_bison             AGA--GACCAA-TCT--CCTCGACTCTCTCTCAAACGCCTCAGGAGGCTTGACTCCCTTT 1180
Bos_taurus_taurus       AGA--G-CCAA-CCT--CGCGACCTCTCTC-CAAACGCCTCAGGAGGCT-GACTCCCATT 1185
Bos_grunniens           AGA--GACCAAATCT--CGCGACCTCTCTC-CAAGCGCCTCAGGAGGCTTGACTCCCTTT 1184
Bos_taurus_indicus      AGAAGGCCCAATCCTTCCGCGAACCTCTCCAAAAACGCCTCAGA--GCCTGACTCCCTTC 1180
                        ***  * ****  **  *     *     *  ** ********   **  ******* *

Bison_bison             ---AGTCC-ACCCAGTGAGCTCCAAGAGAT-ACCCGTCGC--GACTCGAGAGC-AGAGCG 1232
Bos_taurus_taurus       ---TGTCC-ACCCAGTGGAGCTCAAGAGAT-ACCCGTCGC--GACTCGGAGGC-AGAAAG 1237
Bos_grunniens           ---AGTCC-ACCCAGTGAGCTCCAAGAGAT-ACCCGTCGC--GATTCGAGAGC-AGGCGG 1236
Bos_taurus_indicus      GCGAGTCCCACCCAGTGGAGCCCAAGAGATGAACCGTCGCCTGATTCGAGAGCCAGAGCG 1240
                            **** ********     ******** * *******  ** ***   ** **   *

Bison_bison             --GGGTTCTTTGCTTCCACT--CGACA-TGAATGC--TGTCTCCCCGGGTGCGTCTGGAA 1285
Bos_taurus_taurus       TTGGGTTCGTTGCTTCCCCT--CGAGA-TGAATGCC-TGTCTCCCCGGGTGCGTCTTGGA 1293
Bos_grunniens           --GGGTTCTTTGCTTCCACT--CGAGAATGAATGCC-TGTCTCCCCGGGTGCGTCT-GGA 1290
Bos_taurus_indicus      --GCCCTCTTTGCTTCCACTTCCGAGGTGAAATGCCTTGTCTCCCCGG-TG-GTCT--GA 1294
                          *   ** ******** **  ***     *****  *********** ** ****   *

Bison_bison             TTGCAACCCTGAGATCCCTTTCGCCCCCTGGAGAGGAA-CACTGG-CTTCTGGACACGAA 1343
Bos_taurus_taurus       ATGCCACCCCGAG-TCCCGGTCGCCCC-TGGAGAGGAA-CCTCGGGCTTCTGGGCAC-AA 1349
Bos_grunniens           ATGCAACCC-GAGATCCCTGTCGGCCC-TGGAGAGGAA-CATTGG-CTTCTGGACAC-AA 1345
Bos_taurus_indicus      ATGC-ACCCCGAGATCC-TGTCGCCCT---GAGAGAAAACAATTG-CTTCTG--CACAAA 1346
                         *** **** *** ***   *** **    ***** ** *    * ******  *** **

Bison_bison             GCCTAGA-TGA-GGTCTATT-GGCCCTGCA-GT-CACTCGA-GAGCAATCCCC-AGCTTT 1396
Bos_taurus_taurus       GCCTAGA-TGAAGGTCTATCAGGGCCTGCAAGT-CACTCTG-GAGCAATCCCC-AGCTTT 1405
Bos_grunniens           GCCTAGA-TGA-TGTCTATT-GGCCCTGCA-GT-CACTCGA-GAGCAATCCCC-AGCTTT 1398
Bos_taurus_indicus      GCCTAGAATGA-GTCCTATT-GGCCCTCCA-GTACACTCGATGAGCAATCCCCCAGCTTT 1403
                        ******* ***    ****  ** *** ** ** *****   *********** ******

Bison_bison             CCTT-CGCAACTCG- 1409
Bos_taurus_taurus       TCTTTCGCAACTCG- 1419
Bos_grunniens           CCTT-CGCAACTCGA 1412
Bos_taurus_indicus      CCCTTCGCAACTCCA 1418
                         * * ********

Candidate centromere repeat monomer:
>Bison_bison
AATGGAAGATTGGACTTCCCTGGGCCAAACACAAGAGGCATCCTGAATTCCCCGTCGTAA
TTCGAGAATCCTGCCGACACTCGAGAAAATCCACGTGTTCCCCCGTCATCGCAAGATGAA
GCCCTTTCCCGCTACAGTGTCTCAGGAGAAGTCCCACGTTAGGTATTGGAGGTCGAAACG
GTACTTGGCACCTTGATGCGACCACAAAGTGCCCCGACATCCCGGTCCTCCCTCGAGAGG
AACACCGAAGTTTTCCGGCACCACTTCCTCTGAGCCCCTTCTACCCTCCTGATCTGGACA
GGAGGGTCGACTCCCCTGCTTTGTCTGGAAGGGGTTCCCGACCTTCCGGTCGCACCTCAG
GATGAGGCCGGGTCTCACGACGACATTTCAGACGTGGCCTCGTGGGTGGTTCCACATTCC
GAAGGACCCCGATTTCCCGGTCCCCTCTTGATAAGAACCCGATGCCCGGACACCTCTTCG
AACCTCCACCCTGTGAATGAAGTCAACACGAAAGGGCAGTGACTCGCCCGTGCATCGTCG
GGAAAAAACCCCCAGGTTCCAAATACCGCTCGACAAGTGGCCTGTCTCCCCGGGAAACAC
CTCGAGAGGCAAGCGGAGTTCCATGCCTCACCCAAGACGAGGCCTGACTCTCCCTGTCCC
AAGTCTGCAGGGACCTTGCGATCAGAGTCTGAAGTCAGAGGAACCCTGAGGTTCCTGCCT
CAACTGGAGATGAGGCCCTCTTCCAATGCACCAAACCCAGTGGGAGTGCCGAGAAGCCCT
CCCACCTCCAGTTTTCCCTGACTTCTCAGAGCCACCATGAGAAGCCCCCTGAGGTCACCT
GCACAAGTCAAGGGAAGCCAGGTTTTCTGCCTCAACCCGAGAAAGACCTCGAGAGACCTT
CTTCAACACGTCTCGAGCCAGATTCCCCTAACAGTGACTCGAGAGCAATGACGCGCTCCC
CCTCGCCATTGCGCATGGAGACCCCGACTTCCCTGGCGCCCACGAGAGCTCACTGACCTC
GCCGTCGTACACTAGGAAAAACCGCACACTGTGGCGCCAGCTCGAGAACAACCCTGAGCC
CTCCCCATCATCGCGAGTTGAGGGCCTTCGTCTCCTGTATGGCCTAGAGACCAATCTCCT
CGACTCTCTCTCAAACGCCTCAGGAGGCTTGACTCCCTTTAGTCCACCCAGTGAGCTCCA
AGAGATACCCGTCGCGACTCGAGAGCAGAGCGGGGTTCTTTGCTTCCACTCGACATGAAT
```

```
GCTGTCTCCCCGGGTGCGTCTGGAATTGCAACCCTGAGATCCCTTTCGCCCCCTGGAGAG
GAACACTGGCTTCTGGACACGAAGCCTAGATGAGGTCTATTGGCCCTGCAGTCACTCGAG
AGCAATCCCCAGCTTTCCTTCGCAACTCG
>Bos_taurus_taurus
AATGGAAGATTGGACTTCCTGGGCAACACAAGAAGGCCATCACTGATTTCCCCGTTCGTA
ACTCGAGATTCCGCCGCCAAACTCGAGACACCAACGTGGATTCCCCCGTCATCGCACAAG
ATTGAAGCCCTTTCCGCTACAGCGTCTCAGGAGAAGTCCCACGTTAGGAATTGAGCGGTG
GAACGGTACTTGGCACCCTTGACGGCGGCCCCACAAAGTTCCCCGGCATCCCGTCTCCCT
GGAGAGGAACACCGAGTTTCCGGCACCACTTCCTCTGAGCCCCTTCTACCCTCCTGATCT
CGACAGGGAGGGTCGACTCCCCTGCTTTGTCTGGAAGGGGTTCCCGACCTTCCGGTCGCA
CCTCAGGATGAGGCCGGGCTCACGACGACATTCCAGACGTGGCCTCGTGGGTGCTTCCAC
ATTGCGAAGCACCCCGATTTCCCGGTCCCCTCTTGGTAAGAACCCGATGCCCGGACACCT
CTCGAACTCCACCCTGTGAATGAGGTCAACACGAAGGGGCAGTGCCCGCCCGTGCATCGT
CCGGAAAGAACCCCCAGGTTCCAAATACAGCTCGACAAGTGGCCTCTCTCCCCGGGGACA
CCTCGAGAGGCAAGCGGAGTTCCATGCCTCAACCCAAGACGAGGACCTGACTCTCCTGTC
CCCAGTCTGCAGGGACCCTGCGATTCGGAGTCTGAAATCAGAGGAACCCTGAGGTTCCTG
CCTCAACTGGAGATGGAGGCCCTCTTCCAATGCACCAAAACCCCGTGGAGTCCCGAGAGG
CCCCTCCCACCTCCAGTTCCGCTGATTCTCAGAGCCCACCATGAGAAGCCCCCTGAGGTC
ACCTGCACAAGTCGAGGGAAGCCAAGGGTTCCCTGCCTCAACCCGAGAAAGACCTCGAGA
GACCTTCTTCAACATCGTCTCGAGGCCAGGTTCCCTACCAGGACTCGAGGGCAATGACGC
GCTCCCCCTCGCCACTCGCGTGGAGACCCGACTTCCCTGGCGCCCCACGAGAGGCTCACT
GACCTCGCCGTCGCACCTCGTGAGAAACCGCACCCTGGGGCCGCCGGCTCGAGAACCACC
CGAGACTCCCCCGTCATCGCGGAGATGAGGGCCTTCGTCTCCTGCATGGGCCTAGAGCCA
ACCTCGCGACCTCTCTCCAAACGCCTCAGGAGGCTGACTCCCATTTGTCCACCCAGTGGA
GCTCAAGAGATACCCGTCGCGACTCGGAGGCAGAAAGTTGGGTTCGTTGCTTCCCCTCGA
GATGAATGCCTGTCTCCCCGGGTGCGTCTTGGAATGCCACCCCGAGTCCCGGTCGCCCCT
GGAGAGGAACCTCGGGCTTCTGGGCACAAGCCTAGATGAAGGTCTATCAGGGCCTGCAAG
TCACTCTGGAGCAATCCCCAGCTTTTCTTTCGCAACTCG
>Bos_taurus_indicus
AATGGAAGATTGAACTTGCCCTGGGCCAACCAAAAGGCATCCTGACTTCCCCGTCGTAAC
TCAGAATCCCCGCCCGTAACTCGAGAAAAACCACGTGGCTCCCCGTCCATCCGCAAGATG
AAGGCCCTTCCCGCAACAGCCGGCCCTCCAGGAGAAGTCCCACGTAGGATTGGAGGTCGA
AAGGGCACTTGGCGCCCTTGATGCGACCCACAAAGTTCCCCGAAATCCCCGGTCTCCCTC
GAGAGGAACACTGAGGCTTTCGCACCCCCTCCTCTGACCCTTTTCTCCCCCTCCTGAATC
TGGACAGGAGGTCGACTCCCCTGCTTGTCTGGAAGGGGTTCCGACCTTCCGTCCACCTCC
AGGAATGAGGCCGGTCTCAACGAAGACCATTCCAACGTGCCCCTCCGTGGGTGGTTCCCA
CATTCGTAGGACCCCGATTCCCGTCCCCTCCTGGATAAGAACCCGATGCCGGACCACCTC
TCCGAACTCCAACCCTGGAATGAAGTCAACACGAAGGGCCAATTTTTCCGTGCATCGTTC
AGAAAAACCCAGTTCCAAATACAGCTCGACAAGCCGGCTCTTCTTCCCCCGGGACCATCT
CGAGATGCAAGCGGAGTTCCATGCCTCAAACCAAAGACAGCCGACTCTCCTGTCCCAGTC
CTGCAGACCTGCGATCGGAAGTCTGAATCAAGGTACCCTGCGGTTCCGCCCCTCAACTGG
AGATGAGGCCTCTTCCAATGCACCAAAACCCAGTGGGGTCCCGAGAGGCCCTTCCCACCT
CCAGGTTCCCTGCTTCTCAGAGCCACCATGGAGAAGCCCCTGAGGTCACCTGCAACAAGT
CGAGGAACCCAGGGTTTCCTGCCTCAACCGAAAAGACCTCGAGAGACCTTCTCAACACGT
CTCTGAGGCCAATCCCTAACATGGCTCGGGAATCCAGTGACGCGCTCCCCCCCACCACTT
CGCACTGGAGAACCCGACTTCCCTGGCACCCCACCAGAGGCCTCACTGACCTCGCCGCGT
ACCTCCAGTGAAGAAAACACCACCGGGGCCGCCGCTCGAAGAACAAACCCCCGAGAATTC
CACCCTCATCGAGAGATGAGGGCCCTCCGCCCCCTCAGGCCTAGAAGGCCCAATCCTTCC
GCGAACCTCTCCAAAAACGCCTCAGAGCCTGACTCCCTTCGCGAGTCCCACCCAGTGGAG
CCCAAGAGATGAACCGTCGCCTGATTCGAGAGCCAGAGCGGCCCTCTTTGCTTCCACTTC
CGAGGTGAAATGCCTTGTCTCCCCGGTGGTCTGAATGCACCCCGAGATCCTGTCGCCCTG
AGAGAAAACAATTGCTTCTGCACAAAGCCTAGAATGAGTCCTATTGGCCCTCCAGTACAC
TCGATGAGCAATCCCCCAGCTTTCCCTTCGCAACTCCA
>Bos_grunniens
ATGGAAGATTGGACTTCCCTGGGCCAACACAAGAGGCATCCTGAATTCCCCGTCGTAACT
CGAGAATCCCGCCGCAACTCGAGAAAAACCACGTGGTTCCCCCGTCATCGCAAGATGAAG
CCCTTTCCCGCTACAGCGTCTCAGGAGAAGTCCCACGTTAGGAATTGGAGGTCGAAACGG
TACTTGGCCACCCTTGATGCGACCCACAAAGTTCCCGACATCCCGGTCTCCCTCGGAGAG
GAACCCCTGAGGTTTTCCGGCCACCACTTCCTCTGAGCCCCTTCTACCCTCCTGATCTGG
ACAGGAGGGTCGACTCCCCTGCTTTTTCTGGAAGGGGTTCCCGACCTTCCGGTCGCACCT
CAGGATGAGGCCGGTCTCACGACGACATTCCAGGACGTGGCCTCGTGGGTGGTTCCACAT
TGCGTAAGACACCCGATTTCCCGGTCCCCTCTTGATAAGAACCCGATGCCCGGACACCTC
TTCCGAACTCCACCCTGTGAATGAAGTCAACACGAAGGGGCAGTGACTCGCCCGTGCATC
GTTCGGGGAAAACCCCCAGGTTCCAAATACAGCTCGACAATGGGCCTCTCTCCCCGGGGA
CACCTCGAGAGGCAAGCGGAGTTCCATGCCTCAACCCAAGACGAGGCCTGACTCTCCTGT
CCCAAGTCTGCAGGGACCCTGCGATCGGTCTGAAATCAGAGGAACCCTGAGGTTCCTGCC
TCAACTGGAGATGAGGCCCTCTTCCAATGCACCAACCCCATGGAGTCCCGAGAGGCCCCT
CCCAACCTCCAGTTTCCCTGACTTCTCAGAGCCACCATGAGAAGCCCCCTGAGGTCACCT
GCACAAGTCGAGGGAACCAGTGTTTCCTGCCTCAACCGAGAAAGACCCCCTCGAGAGACC
TTCTTCAACACGTCTCGAGGCCAGATTCCCCTACCATGACTCCGAGGAGCAATGACGCGC
TCCCCCTCGCCACTTGCATGGAGACCCGACTTCCCTGGGCCCCACGAGAGGCTCACTGAC
```

```
CTCGCCGTCGTACCTCGTGAGAAACCGCACCCTGGGGCCGCCGCTCGAGAACAACCCCGA
GACTCCCCCGTCATCGCGAGATGAGGGGCCTTCGTCTCCTGCATGGCCTAGAGACCAAAT
CTCGCGACCTCTCTCCAAGCGCCTCAGGAGGCTTGACTCCCTTTAGTCCACCCAGTGAGC
TCCAAGAGATACCCGTCGCGATTCGAGAGCAGGCGGGGGTTCTTTGCTTCCACTCGAGAA
TGAATGCCTGTCTCCCCGGGTGCGTCTGGAATGCAACCCGAGATCCCTGTCGGCCCTGGA
GAGGAACATTGGCTTCTGGACACAAGCCTAGATGATGTCTATTGGCCCTGCAGTCACTCG
AGAGCAATCCCCAGCTTTCCTTCGCAACTCGA

##########################################################################################
Caprid

Capra_hircus      --TCCCATCGAGGCTTGCCCACGGGGCCTCTC----GGGATTCCTCTCCCGTCGATGCCG 54
Ovis_aries        TTTTCCCTCGTGTCTTTCCCACGAGGCTTTCCCACGAGGCTTTCCCACAGGGCTGTCCCA 60
                    * ** *** * *** ****** *** *  *     ** ** * * *  * *  * **

Capra_hircus      GGGCCTAAGACCTTGTGTGGAGTCGGTGCCGGAACCTGAGGATTCCTCTCCAGTGCAG-A 113
Ovis_aries        CGTGC--ACACGTGGTG-GGAGTCGAT-CCTCGGCTTGAA-------CGTCAAGGCAGTG 109
                   *  *  * ** * *** ******* * **    * ***        *  **  ****

Capra_hircus      CAGGGATAATGGGGTTCCCTGGAGTCGCCTCAGGGGATCAGGCCTCCTCTCGAATGGTGG 173
Ovis_aries        CAGGGAAAACAGGTTCCTCTGGAATGGACTGACACATCTGGGGGACTCTTGGAATGGTGG 169
                  ****** **  ** * * ***** * * ** *        **   *   * *********

Capra_hircus      CACGAACCTGGAGTTCCTCTCGCCTTTCCTGTGGAGAGCGCCTCCTCTTGCGATGCGACG 233
Ovis_aries        CACGACCCTGGAGTTCCTCTCGCCTTTCCTGTGGAGAGCGCCTCCTCTTGAGATGCGACG 229
                  ***** ******************************************** *********

Capra_hircus      GGAATCCCGGGAATTCTTTCCCTCC-ACGCAGGCACACGAGCCCTCCCCACGAGCTTACA 292
Ovis_aries        GGAACGCCGGGAATTCTTTCCCTACGAAACAGGGAAAGGATCCCTCATCTCGAGCTCG-G 288
                  ****  **************** * *  **** * * ** *****  * ******

Capra_hircus      AGGCGCAAACGGGCCACCTCTGGATGTGAGCGCGACCCTCGTGCTTAAACTCGAGTGCAG 352
Ovis_aries        AGGCGGAAACGGGGCTCCCCTGGATGTGTTCGGGACCCTCGTGCTTCCTCTCGAGTGGAG 348
                  ***** ******* * ** *********  ** *************   ******** **

Capra_hircus      ACAGGGAAAACAGGGAACTCCTGGAATGGCAGCAAACATCTGAAGGACCCTTGGGAAGGT 412
Ovis_aries        AC-GGGTGTGTCGGGAACTTCTTGAGTTGCAGCAAGGGTGTGAAGGACCCTTTGGAAGTT 407
                  ** ***      ******* ** ** * *******   * ************ ***** *

Capra_hircus      CCACACGTAACCTGTGAGTAGCCTCGAGACGCCCCAGCGGAAATGCGCCTCATCTCGACA 472
Ovis_aries        CCAGGGGTTAGATGTGATTAGCCTCGAGAAGCCTCAGCGGAAATGGGCCTCATCTCGCCT 467
                  ***   ** *  ***** *********** *** *********** *********** *

Capra_hircus      GGAGACGAGAACCGCCGGGATTTTCCCGACACGCGGAAGGTCCTCTCGACCTACGACGCG 532
Ovis_aries        GGAGGGCAAAACCTCCTGGATTTTCTCGAGTTGCGGCAGGTGCTCTCGACTTACGACGGG 527
                  ****   * **** ** ******** ***   **** **** ******** ******* *

Capra_hircus      GACAACAGGGACCCGCTCTGGTGGCCGCAGGAAACGCCAGTCCCCATGCGAGTTGCACAC 592
Ovis_aries        GCCCTCAGGGACCCGCTCTGGTGGCCTCAGGAAAGGCCAGTCCCCATGCGAGTTCCTCGG 587
                  * *  ******************** ******* ****************** * *

Capra_hircus      GGGCATCTCGGGAAACCTCTCCAGTCGAAGCAAGGGCCTAAGACCCTGTGTGAAGTCCCA 652
Ovis_aries        GGGCCTTTCGGAATTCCTCTCCCGCTGATGCCGGGGCCTAAGACCTTGTGTGAACTCAGG 647
                  **** * **** *  ******* *  ** **  ************ ******** **

Capra_hircus      GACGGAACCTGTG-ATTACCCTCCAGCGCTCACACGGATAATGGGCCACATCTG-AGTCT 710
Ovis_aries        GCCGGAACCTGAGGATTCCTCTCCAGTGCTGACATGGATCTTGGGGTACTTCTGGAGTCT 707
                  * ********* * *** * ****** *** *** ****  ****  ** **** *****

Capra_hircus      CCCCAGGGGAGAAACTCCTCGTCTCGAGTGGCGGCATGCG--TGCGCTCGACTCACGAGC 768
Ovis_aries        CCCCAGGGGAGTCAGTCCTCGTCTCGAATGCGGGCATGCACTTGCGCTTTCCTCCAGAGC 767
                  ***********  * ************ **  *******   ******   ***  ****

Capra_hircus      GGGAACAGCAGGGACACGCTTCCCGTCGCCTGCAGCAAAGGACCAGTGC 817
Ovis_aries        GGTAGCAGCAGTGTCACGCAGTCCGCCCCGTGGATCAAAGCATCTATGG 816
                  ** * ****** * *****   *** * * ** * ***** * *  **

Centromere repeat monomer:
>Capra_hircus
TCCCATCGAGGCTTGCCCACGGGGCCTCTCGGGATTCCTCTCCCGTCGATGCCGGGGCCT
AAGACCTTGTGTGGAGTCGGTGCCGGAACCTGAGGATTCCTCTCCAGTGCAGACAGGGAT
```

```
AATGGGGTTCCCTGGAGTCGCCTCAGGGGATCAGGCCTCCTCTCGAATGGTGGCACGAAC
CTGGAGTTCCTCTCGCCTTTCCTGTGGAGAGCGCCTCCTCTTGCGATGCGACGGGAATCC
CGGGAATTCTTTCCCTCCACGCAGGCACACGAGCCCTCCCCACGAGCTTACAAGGCGCAA
ACGGGCCACCTCTGGATGTGAGCGCGACCCTCGTGCTTAAACTCGAGTGCAGACAGGGAA
AACAGGGAACTCCTGGAATGGCAGCAAACATCTGAAGGACCCTTGGGAAGGTCCACACGT
AACCTGTGAGTAGCCTCGAGACGCCCCAGCGGAAATGCGCCTCATCTCGACAGGAGACGA
GAACCGCCGGGATTTTCCCGACACGCGGAAGGTCCTCTCGACCTACGACGCGGACAACAG
GGACCCGCTCTGGTGGCCGCAGGAAACGCCAGTCCCCATGCGAGTTGCACACGGGCATCT
CGGGAAACCTCTCCAGTCGAAGCAAGGGCCTAAGACCCTGTGTGAAGTCCCAGACGGAAC
CTGTGATTACCCTCCAGCGCTCACACGGATAATGGGCCACATCTGAGTCTCCCCAGGGGA
GAAACTCCTCGTCTCGAGTGGCGGCATGCGTGCGCTCGACTCACGAGCGGGAACAGCAGG
GACACGCTTCCCGTCGCCTGCAGCAAAGGACCAGTGC
>Ovis_aries
TTTTCCCTCGTGTCTTTCCCACGAGGCTTTCCCACGAGGCTTTCCCACAGGGCTGTCCCA
CGTGCACACGTGGTGGGAGTCGATCCTCGGCTTGAACGTCAAGGCAGTGCAGGGAAAACA
GGTTCCTCTGGAATGGACTGACACATCTGGGGGACTCTTGGAATGGTGGCACGACCCTGG
AGTTCCTCTCGCCTTTCCTGTGGAGAGCGCCTCCTCTTGAGATGCGACGGGAACGCCGGG
AATTCTTTCCCTACGAAACAGGGAAAGGATCCCTCATCTCGAGCTCGGAGGCGGAAACGG
GGCTCCCCTGGATGTGTTCGGGACCCTCGTGCTTCCTCTCGAGTGGAGACGGGTGTGTCG
GGAACTTCTTGAGTTGCAGCAAGGGTGTGAAGGACCCTTTGGAAGTTCCAGGGGTTAGAT
GTGATTAGCCTCGAGAAGCCTCAGCGGAAATGGGCCTCATCTCGCCTGGAGGGCAAAACC
TCCTGGATTTTCTCGAGTTGCGGCAGGTGCTCTCGACTTACGACGGGGCCCTCAGGGACC
CGCTCTGGTGGCCTCAGGAAAGGCCAGTCCCCATGCGAGTTCCTCGGGGGCCTTTCGGAA
TTCCTCTCCCGCTGATGCCGGGGCCTAAGACCTTGTGTGAACTCAGGGCCGGAACCTGAG
GATTCCTCTCCAGTGCTGACATGGATCTTGGGGTACTTCTGGAGTCTCCCCAGGGGAGTC
AGTCCTCGTCTCGAATGCGGGCATGCACTTGCGCTTTCCTCCAGAGCGGTAGCAGCAGTG
TCACGCAGTCCGCCCCGTGGATCAAAGCATCTATGG

#################################################################################################
Primates

Gorilla_gorilla_graueri            CAAAAAGAGTGTTTCAAA-CTGCTGTATCAAAAGAAAGGTTCAACTCTGT 49
Gorilla_gorilla_gorilla            CAAAAAGAGGGTTTCAAAACTGCTCTGTCAAAAGAAAGGTTAAACTCTGT 50
homo_sapiens                       CAAAAAGAGTGTTTCAAAACTGCTCAATCAAAAGAAAGGTTCAACTCTGT 50
pongo_abelii                       CAAAAAGAGTGTTTCAAAACTGCTCAATCAAAAGAAAGGTTCAACTCTGT 50
nomascus_leucogenys                CAAAAAGAGTGTTTCAAAACTGCTCAGTCAAAAGAAAGGTTCAACTCTGT 50
pongo_pygmaeus                     CAAAAAGACTGTTTCCAAACTGCTCAATCAAAAGAAAGGTTCAACTCTGT 50
hylobates_concolor                 CAAAAAGAGTGTTTATAAACTGCTCAATCAAAAGAAAGGTTCAACTCTGT 50
pan_paniscus                       CAAAAAGAGTGTTTCAAAACTGCTCTATGAAAAGAAAGGTTCAACTCTGT 50
pan_troglodytes_schweinfurtii      CAAAAAGAGTGTTTCAAAACTGCTCTATCAAAAGAAAGGTTCAACTCTGT 50
pan_troglodytes_troglodytes        CAAAAAGAGTGTTTCAAAACTGCTCTATCAAAAGAAAGGTTCAACTCTGT 50
macaca_mulatta                     CAAGAACTGGCT-AGCGAAAGGCTCCTTGAAAG-AAAGATGTAACTCTGT 48
Macaca_fascicularis                CAAGAACTGTCTTAGC-AAAGGCTTCTTGAGGGGAAAGCTGTAACTCTGT 49
papio_hamadryas                    CAAGAAATAGGCTAGCGAAAGGATCCATGAGAAGAAAGATGTAACTCTGT 50
                                   *** **           *   * *   * *    **** *  ********

Gorilla_gorilla_graueri            TAGTTGAGGACACACATCACAAAGAAGTTTCTGAGAATGCTTCTGTCTAG 99
Gorilla_gorilla_gorilla            GAGTGGAACACACACAACACAAAGAAGTTACTGAGAATGATTCTCTCTAG 100
homo_sapiens                       GAGTTGAATGCACACATCACAAAGAAGTTTCTCAGAATGCTTCTGTCTAG 100
pongo_abelii                       GAGATGAATGCACACATCACAAAGAAGTTTCTCAGAATGCTTCTGTCTAA 100
nomascus_leucogenys                TAGATGAATGCACAGATTAGAAAGAAGTTTCACAGAATGCTTCTGTGTAG 100
pongo_pygmaeus                     GAGATGAATGCACACATCACAAAGAAGTTTCTCAGAATGCTTCTGTCTAG 100
hylobates_concolor                 GAGATGAATGCACACATCACAAAGAAGTTTCTCAGAATGCTTCTGTCTAG 100
pan_paniscus                       GAGTTGAAAGCACACATCACAAAGAAGTTTCTGAGAATGCTTCTGTGTAG 100
pan_troglodytes_schweinfurtii      GAGTTGAATGCACACATCACAAAGAAGTTTCTGAGAATGCTTCTGTCTAG 100
pan_troglodytes_troglodytes        GAGTTGAATGCACACATCACAAAGAAGTTTCTGAGAATGCTTCTGTCTAG 100
macaca_mulatta                     GAGATGAATTCACAGAACACAAAGAAGTTTCTCAGAAAGCTTCTTTCTCT 98
Macaca_fascicularis                GAGATGATTTCACAGAACACAAAGCAGTTTCTCAGAAAGCTTCTTTCTCT 99
papio_hamadryas                    GAGATGAATTCACAGAACACAAAGCAGTTTCTCAGAAAGCTTCTTTCCAG 100
                                    **  **   **** *  * **** **** *  **** * **** *

Gorilla_gorilla_graueri            ATTTTATATGAAGATATTCCCGTTTCCAACGAAATCTTCAGAGTA-TCC- 147
Gorilla_gorilla_gorilla            TCATTAGACGAAGATAATCCCGTTTCCAACGAAAGCCCCAAAGAGCTCC- 149
homo_sapiens                       TTTTTATGTGAAGATATTTCCTTTTCCACCATAGGCCTCAAAGCGCTCC- 149
pongo_abelii                       TTTTTATGTGAAGATATTTCCTTTTCCACCATAGGCCTCAAAGCGCTCC- 149
nomascus_leucogenys                TTTTTATTTGAAGATATTTCCTTTTTCACCATAGGCCTCAAAGCGCTCC- 149
pongo_pygmaeus                     TTTTTATGTGAAGATATTTCCTTTTTCACCATAGGCCTCAAAGCGCTCC- 149
hylobates_concolor                 TTTTTATGTGAAGATATTTCCTTTTCCACTATAGGCCCTAAAGTGCTCC- 149
pan_paniscus                       TTTTTATGTGAAGATATTTCCTTTTTCAAAGTAGGCCTCAAAGCGCTCC- 149
pan_troglodytes_schweinfurtii      TTTTTATGTGAAGATATTTCCTTTTCCAACATAGGCCTCAAAGCGCTCC- 149
pan_troglodytes_troglodytes        TTTTTATGTGAAGATATTTCCTTTTCCACCATAGGCCTCAAAGGGCTCC- 149
```

```
macaca_mulatta                   TTTTTATCGGAGGATATTTCCTTTGGCACCATAGCCCTCAAAGGGATCCC 148
Macaca_fascicularis              TTTTTATCTGAGGATATTTCCTTTTTCCCTATAGTCTTCTATGGGCTTCG 149
papio_hamadryas                  TTTTCATCTGAGGATATTTCCTTTTTCACCATAGCCCTCAATGGGCTTCC 150
                                    * *   ** **** * ** **  *     *  *      *   * *

Gorilla_gorilla_graueri          AAATATCCACTTGCAGATTCTA 169
Gorilla_gorilla_gorilla          AAATATCCACTTGCAAACTCCA 171
homo_sapiens                     AAATATCCACATGCAGATTCTA 171
pongo_abelii                     AAATATCCACTTGCAGATTCTA 171
nomascus_leucogenys              AAATGTCCACTTGCAGATTCTA 171
pongo_pygmaeus                   AAATATCCCTTTGCAGATTCTA 171
hylobates_concolor               AATTATCCACTTGCAGATCCTA 171
pan_paniscus                     AAATGTCCACTTGCAGATTCTA 171
pan_troglodytes_schweinfurtii    AAATATCCACTTGCAGATTCTA 171
pan_troglodytes_troglodytes      AAATATCCACTTGCAGATTCTA 171
macaca_mulatta                   AAATATCACTTCGCCGATTCCA 170
Macaca_fascicularis              AAATATCTCTTTTCCAATTCCA 171
papio_hamadryas                  AAATATCACTTTTCAAATTCCA 172
                                 ** * **      *  *  * *


callithrix_jacchus       GAAGGAACGTGCTCC--ACTCTCTGAATCTAAACGCGGATTCAAC--TCCCTTAGTTAAG 56
Saimiri_boliviensis      GAAGAAACGTGTTTCTACCTCGCTGAGACT-------GTTTCTACATTCTCCTAAGAAAG 53
ateles_geoffroyi         CACAGAAAGGGTTTCTAAACTGCTGCAAACACACACGAGTTTAAC--TCTGTAAGTTGAA 58
                          *   ** * * * *       ***              **  **  **    *    *

callithrix_jacchus       CGAACGCACAGGAGAGCAGTTTGAAGGATACGTTCGTTTTGTGTTTTAGACGGAGATTTA 116
Saimiri_boliviensis      -GGTTTCTTAACTGCGTGCTT--ACACACATCTTTTTTCCGTGTTTGAAACGCATTCTTA 110
ateles_geoffroyi         TGCACACATTGACAAGCATTCAGTTAGATTGCTCCGTTTTG-GTTTTAGAGCGAGATATT 117
                          *    *        *   *       *    *   **  * **** * *   *    *

callithrix_jacchus       CAGCTATTTCTGAAATGCTTGGATTTGGCTGGAAAAATGCGTTTTCCGC-TGCTTCACAG 175
Saimiri_boliviensis      CTGCTAACTCTTATATGCGTCTCTAGGGCTGGAAATGACTGTTT-CTGCATTCTCCAAAG 169
ateles_geoffroyi         CCTCTGTACAGCAAATGCTCCCTTTCGCTCTGGAAAATCCAAACGCAG--TTCCTAGAAG 175
                         *  **       * ****     *  *    * **          * *  * *     **

callithrix_jacchus       AAAGGGTTTACAAACTC-CTGCAAACGCATATCAGTTTAACTCTGTAAGTTGAA-TGCAC 233
Saimiri_boliviensis      AAAGGGTTTCTGAACTG-CTGCAAACACACATCAGTTTAACTCTGGAAGTTGAA-TACAC 227
ateles_geoffroyi         AAACGTGTTGCTAACTCGCTG-AATGAAACTCCGCTTCAGCTCCCTTAGTTGAAACGAAC 234
                         *** *  **   ****  *** **    *   *  ** * ***    *******    **

callithrix_jacchus       ACATTGAAGACCGTTCTGTTAGGTAGCTGCGTTTTG-GTTCTAGAAGCA--GATATTTCT 290
Saimiri_boliviensis      ACATTGACAACCATTCTGCTAGATAGCTCCGTTTTG-GTTTTAGAGCAA--GGTATTTCT 284
ateles_geoffroyi         ACACAGCAGAGCAGTTTGAATGATGCGTTTCTTCTGTGTTTTAGACGCACTATTACTGCT 294
                         ***  *   * *  * **   * *    *   ** ** *** ****   *    ** * **

callithrix_jacchus       CAATACAGCGTTTACTCCCATTCACGCTGGAA-TAGCAAAAC-GCAGCTCCTA 341
Saimiri_boliviensis      CTTTACAGCCTGTTCTCCCATCCACTCTGGAA-TGTCCAAAC-GCAGCTTTTA 335
ateles_geoffroyi         TTTTCTGACGTG--CCTGGATTTG-GCTGGAAATGACAGTTTTGCATCTCTG- 343
                            *    * *   *    **     ****** *  *      *** **


>homo_sapiens
CAAAAAGAGTGTTTCAAAACTGCTCAATCAAAAGAAAGGTTCAACTCTGTGAGTTGAATGCACACATCACAAAGAAGTTTCTCAGAATGCTTCTGTC
TAGTTTTTATGTGAAGATATTTCCTTTTCCACCATAGGCCTCAAAGCGCTCCAAATATCCACATGCAGATTCTA
>Gorilla_gorilla_graueri
CAAAAAGAGTGTTTCAAACTGCTGTATCAAAAGAAAGGTTCAACTCTGTTAGTTGAGGACACACATCACAAAGAAGTTTCTGAGAATGCTTCTGTCT
AGATTTTATATGAAGATATTCCCGTTTCCAACGAAATCTTCAGAGTATCCAAATATCCACTTGCAGATTCTA
>Gorilla_gorilla_gorilla
CAAAAAGAGGGTTTCAAAACTGCTCTGTCAAAAGAAAGGTTAAACTCTGTGAGTGGAACACACACAACACAAAGAAGTTACTGAGAATGATTCTCTC
TAGTCATTAGACGAAGATAATCCCGTTTCCAACGAAAGCCCCAAAGAGCTCCAAATATCCACTTGCAAACTCCA
>hylobates_concolor
CAAAAAGAGTGTTTATAAACTGCTCAATCAAAAGAAAGGTTCAACTCTGTGAGATGAATGCACACATCACAAAGAAGTTTCTCAGAATGCTTCTGTC
TAGTTTTTATGTGAAGATATTTCCTTTTCCACTATAGGCCCTAAAGTGCTCCAATTATCCACTTGCAGATCCTA
>macaca_mulatta
CAAGAACTGGCTAGCGAAAGGCTCCTTGAAAGAAAGATGTAACTCTGTGAGATGAATTCACAGAACACAAAGAAGTTTCTCAGAAAGCTTCTTTCTC
TTTTTATCGGAGGATATTTCCTTTGGCACCATAGCCCTCAAAGGGATCCCAAATATCACTTCGCCGATTCCA
>nomascus_leucogenys
CAAAAAGAGTGTTTCAAAACTGCTCAGTCAAAAGAAAGGTTCAACTCTGTTAGATGAATGCACAGATTAGAAAGAAGTTTCACAGAATGCTTCTGTG
TAGTTTTTATTTGAAGATATTTCCTTTTTCACCATAGGCCTCAAAGCGCTCCAAATGTCCACTTGCAGATTCTA
>pan_paniscus
```

```
CAAAAAGAGTGTTTCAAAACTGCTCTATGAAAAGAAAGGTTCAACTCTGTGAGTTGAAAGCACACATCACAAAGAAGTTTCTGAGAATGCTTCTGTG
TAGTTTTTATGTGAAGATATTTCCTTTTTCAAAGTAGGCCTCAAAGCGCTCCAAATGTCCACTTGCAGATTCTA
>pan_troglodytes_troglodytes
CAAAAAGAGTGTTTCAAAACTGCTCTATCAAAAGAAAGGTTCAACTCTGTGAGTTGAATGCACACATCACAAAGAAGTTTCTGAGAATGCTTCTGTC
TAGTTTTTATGTGAAGATATTTCCTTTTCCACCATAGGCCTCAAAGGGCTCCAAATATCCACTTGCAGATTCTA
>pan_troglodytes_schweinfurtii
CAAAAAGAGTGTTTCAAAACTGCTCTATCAAAAGAAAGGTTCAACTCTGTGAGTTGAATGCACACATCACAAAGAAGTTTCTGAGAATGCTTCTGTC
TAGTTTTTATGTGAAGATATTTCCTTTTCCAACATAGGCCTCAAAGCGCTCCAAATATCCACTTGCAGATTCTA
>papio_hamadryas
CAAGAAATAGGCTAGCGAAAGGATCCATGAGAAGAAAGATGTAACTCTGTGAGATGAATTCACAGAACACAAAGCAGTTTCTCAGAAAGCTTCTTTC
CAGTTTTCATCTGAGGATATTTCCTTTTTCACCATAGCCCTCAATGGGCTTCCAAATATCACTTTTCAAATTCCA
>pongo_abelii
CAAAAAGAGTGTTTCAAAACTGCTCAATCAAAAGAAAGGTTCAACTCTGTGAGATGAATGCACACATCACAAAGAAGTTTCTCAGAATGCTTCTGTC
TAATTTTTATGTGAAGATATTTCCTTTTCCACCATAGGCCTCAAAGCGCTCCAAATATCCACTTGCAGATTCTA
>pongo_pygmaeus
CAAAAAGACTGTTTCCAAACTGCTCAATCAAAAGAAAGGTTCAACTCTGTGAGATGAATGCACACATCACAAAGAAGTTTCTCAGAATGCTTCTGTC
TAGTTTTTATGTGAAGATATTTCCTTTTTCACCATAGGCCTCAAAGCGCTCCAAATATCCCTTTGCAGATTCTA
>Macaca_fascicularis
CAAGAACTGTCTTAGCAAAGGCTTCTTGAGGGGAAAGCTGTAACTCTGTGAGATGATTTCACAGAACACAAAGCAGTTTCTCAGAAAGCTTCTTTCT
CTTTTTTATCTGAGGATATTTCCTTTTTCCCTATAGTCTTCTATGGGCTTCGAAATATCTCTTTTCCAATTCCA
>ateles_geoffroyi
CACAGAAAGGGTTTCTAAACTGCTGCAAACACACACGAGTTTAACTCTGTAAGTTGAATGCACACATTGACAAGCATTCAGTTAGATTGCTCCGTTT
TGGTTTTAGAGCGAGATATTCCTCTGTACAGCAAATGCTCCCTTTCGCTCTGGAAAATCCAAACGCAGTTCCTAGAAGAAACGTGTTGCTAACTCGC
TGAATGAAACTCCGCTTCAGCTCCCTTAGTTGAAACGAACACACAGCAGAGCAGTTTGAATGATGCGTTTCTTCTGTGTTTTAGACGCACTATTACT
GCTTTTTCTGACGTGCCTGGATTTGGCTGGAAATGACAGTTTTGCATCTCTG
>callithrix_jacchus
GAAGGAACGTGCTCCACTCTCTGAATCTAAACGCGGATTCAACTCCCTTAGTTAAGCGAACGCACAGGAGAGCAGTTTGAAGGATACGTTCGTTTTG
TGTTTTAGACGGAGATTTACAGCTATTTCTGAAATGCTTGGATTTGGCTGGAAAAATGCGTTTTCCGCTGCTTCACAGAAAGGGTTTACAAACTCCT
GCAAACGCATATCAGTTTAACTCTGTAAGTTGAATGCACACATTGAAGACCGTTCTGTTAGGTAGCTGCGTTTTGGTTCTAGAAGCAGATATTTCTC
AATACAGCGTTTACTCCCATTCACGCTGGAATAGCAAAACGCAGCTCCTA
>Saimiri_boliviensis
GAAGAAACGTGTTTCTACCTCGCTGAGACTGTTTCTACATTCTCCTAAGAAAGGGTTTCTTAACTGCGTGCTTACACACATCTTTTTTCCGTGTTTG
AAACGCATTCTTACTGCTAACTCTTATATGCGTCTCTAGGGCTGGAAATGACTGTTTCTGCATTCTCCAAAGAAAGGGTTTCTGAACTGCTGCAAAC
ACACATCAGTTTAACTCTGGAAGTTGAATACACACATTGACAACCATTCTGCTAGATAGCTCCGTTTTGGTTTTAGAGCAAGGTATTTCTCTTTACA
GCCTGTTCTCCCATCCACTCTGGAATGTCCAAACGCAGCTTTTA
```

#################################################################################################

Mice

```
Mus_musculus                 TAGGACGTGAAATATGGCGAGAAAACTGAAAATCATGGAAAATGAGAAAT 50
Mus_famulus_domesticus       TAGGACCTGGAATATGGCAAAAAACCTGAAAAACGTGGAAAATGAGAAAA 50
                             ****** ** ******** * *** ******* * ** ***********

Mus_musculus                 GCCCACTT 58
Mus_famulus_domesticus       TCACACTG 58
                              * ****
```

Candidate centromere repeat monomer:

```
>Mus_musculus
TAGGACGTGAAATATGGCGAGAAAACTGAAAATCATGGAAAATGAGAAATGCCCACTT
>Mus_famulus_domesticus
TAGGACCTGGAATATGGCAAAAAACCTGAAAAACGTGGAAAATGAGAAAATCACACTG
```

#################################################################################################

Flying lemurs

```
cynocephalus_variegatus      CTCCCTGGAGGTTCAGCTTCTGCAAGCCACTTTAACTTTAAGAAATGAGA 50
cynocephalus_volans          CTCCCCGGAGGTTGAGCTTTCAGAAGCTAGTTTAACTCTAAGGGAGGAAG 50
                             ***** ******* *****    **** * ******* ****  * **

cynocephalus_variegatus      GAGCAGAATATGCTTCTCACATCTAGCATGCAGGAATTCAGGTTTGCAAG 100
cynocephalus_volans          CAGCAGAATGTGCCTCTCACATCTAGCCTGTAGGAATTCAGGTTGGCAAG 100
                              ******** *** ************* ** ************* *****

cynocephalus_variegatus      TTCTATGTGAAAGCCCATTGAAGCCTATGGGGGAAACGGCTTCTGAGACT 150
cynocephalus_volans          TTCTATGGGGAAGCCCATTGCTGCCTATGGTGGAAACGGCATGGGAGATT 150
                             ******* * **********  ******** ********* *   **** *

cynocephalus_variegatus      TCTGTTTCAAAATCTCTGTCAAAAAGCATCAGAGAAGCTTCCCCGCTTAG 200
cynocephalus_volans          TCAGTTTCAAAATCTCTGTGAAAAAGCATCAGAGAAGCTTCTCCGTTTAG 200
                             ** *************** ******************** *** ****
```

```
cynocephalus_variegatus      AATCCAGCATAACTCACTGAACCGAAGATGTGGCAGTTACAAGAGAAGTT 250
cynocephalus_volans          AATCCAGCATATCTCACAGAACCGAAGAAGTGGTAGTTAGAAGGGAGTTA 250
                             *********** ***** ********** **** ***** *** **  *

cynocephalus_variegatus      GAGCTAAGAGAAGTTTGTTTTCTGAAATTAGAAGCAAAATCACACTGCAA 300
cynocephalus_volans          GAGCTGCAAGCAGTTTGCTTTGGGAAATTAGACGCGAAATGAAACTGCAA 300
                             *****   ** ****** ***  ********* ** **** * *******

cynocephalus_variegatus      ACGGCATTCAAGCAGGCTTG-AAAAGTGTGGTGCATATCTCTGCCAAAAA 349
cynocephalus_volans          ATG-CCTTCCAACAGGCTTGGAAAAGTGTGTTCTATATCTCTGCCAAACA 349
                             * * * *** * ******** ********* *  ************** *

cynocephalus_variegatus      CA 351
cynocephalus_volans          AA 351
                              *
```

Candidate centromere repeat monomer:

```
>cynocephalus_variegatus
CTCCCTGGAGGTTCAGCTTCTGCAAGCCACTTTAACTTTAAGAAATGAGAGAGCAGAATA
TGCTTCTCACATCTAGCATGCAGGAATTCAGGTTTGCAAGTTCTATGTGAAAGCCCATTG
AAGCCTATGGGGGAAACGGCTTCTGAGACTTCTGTTTCAAAATCTCTGTCAAAAAGCATC
AGAGAAGCTTCCCCGCTTAGAATCCAGCATAACTCACTGAACCGAAGATGTGGCAGTTAC
AAGAGAAGTTGAGCTAAGAGAAGTTTGTTTTCTGAAATTAGAAGCAAAATCACACTGCAA
ACGGCATTCAAGCAGGCTTGAAAAGTGTGGTGCATATCTCTGCCAAAAACA
>cynocephalus_volans
CTCCCCGGAGGTTGAGCTTTCAGAAGCTAGTTTAACTCTAAGGGAGGAAGCAGCAGAATG
TGCCTCTCACATCTAGCCTGTAGGAATTCAGGTTGGCAAGTTCTATGGGGAAGCCCATTG
CTGCCTATGGTGGAAACGGCATGGGAGATTTCAGTTTCAAAATCTCTGTGAAAAAGCATC
AGAGAAGCTTCTCCGTTTAGAATCCAGCATATCTCACAGAACCGAAGAAGTGGTAGTTAG
AAGGGAGTTAGAGCTGCAAGCAGTTTGCTTTGGGAAATTAGACGCGAAATGAAACTGCAA
ATGCCTTCCAACAGGCTTGGAAAAGTGTGTTCTATATCTCTGCCAAACAAA
```

Supplementary Figure S2: Centromere repeats in primates only conserved among apes and monkeys
The centromere repeat sequences of the more basal primates (tarsiers and prosimians) do not show sequence similarity. Closer inspection of basal primate genomes did not reveal any sequences with sequence similarity to the ape andmonkey centromere repeat.

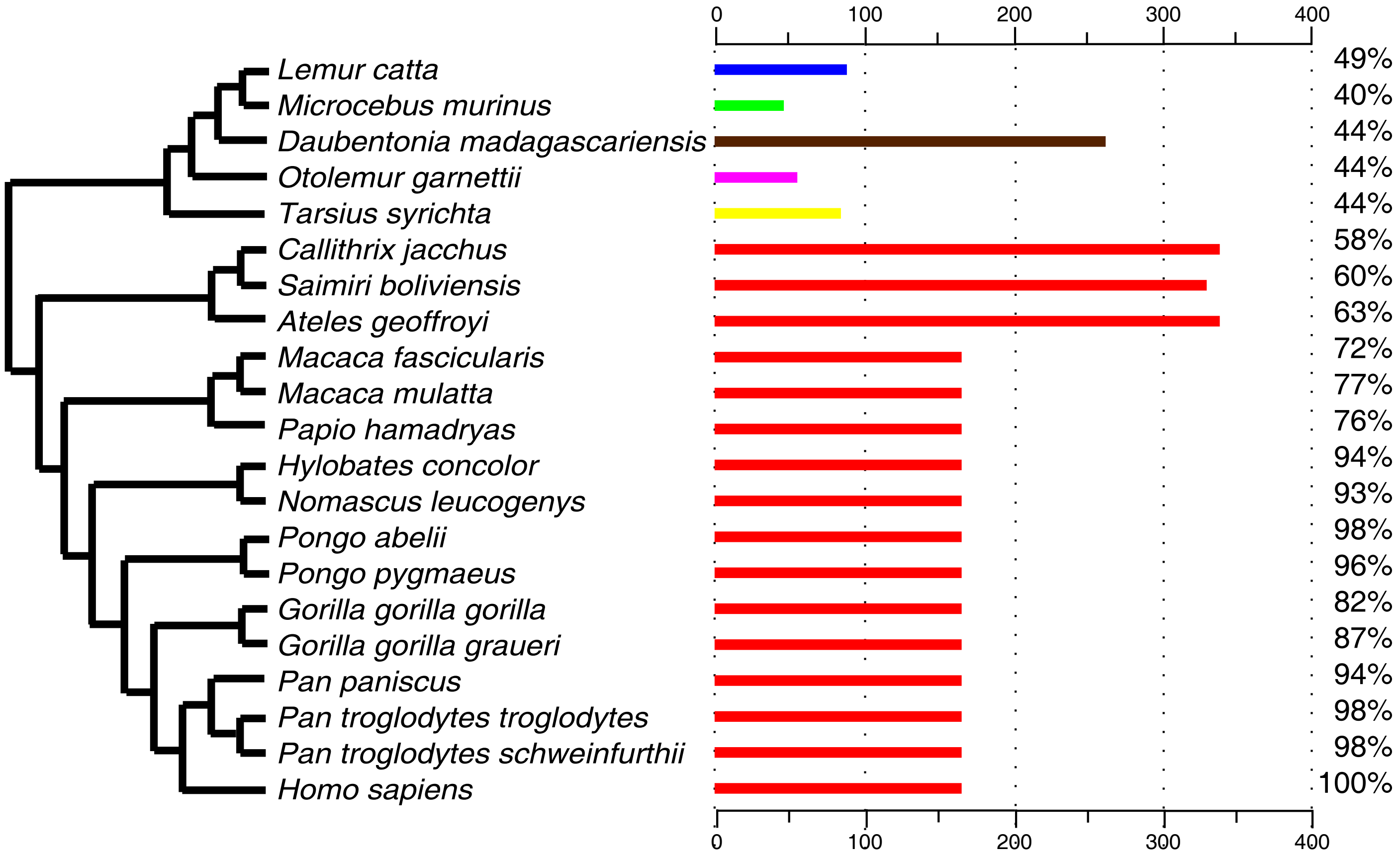

Supplementary Figure 3A: No correlation was observed between repeat length, GC content, kingdom or genomic fraction.

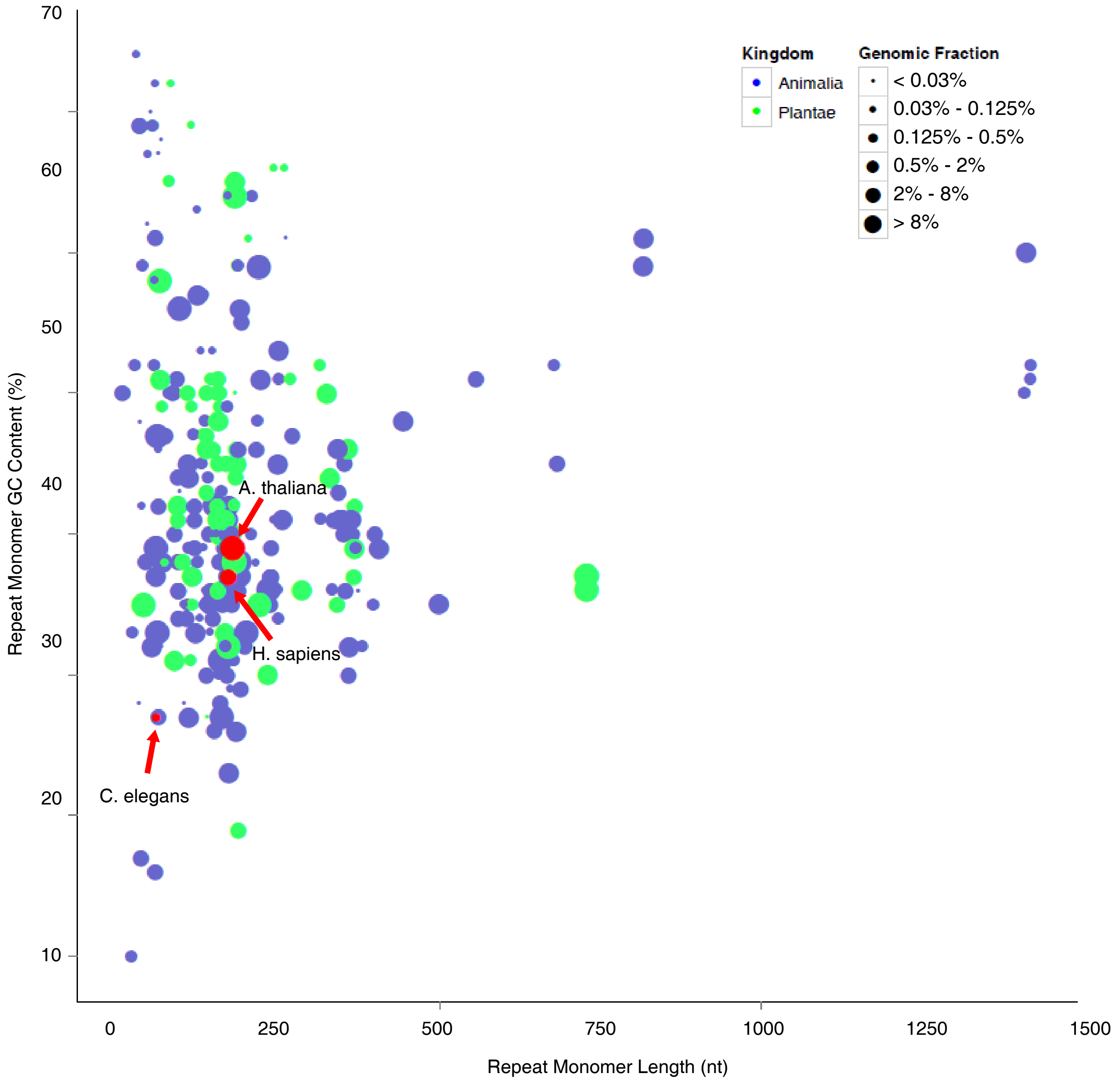

Supplementary Figure S3B: We did not observe a correlation between genome size and chromosome number versus either repeat length, GC content or genomic fraction.

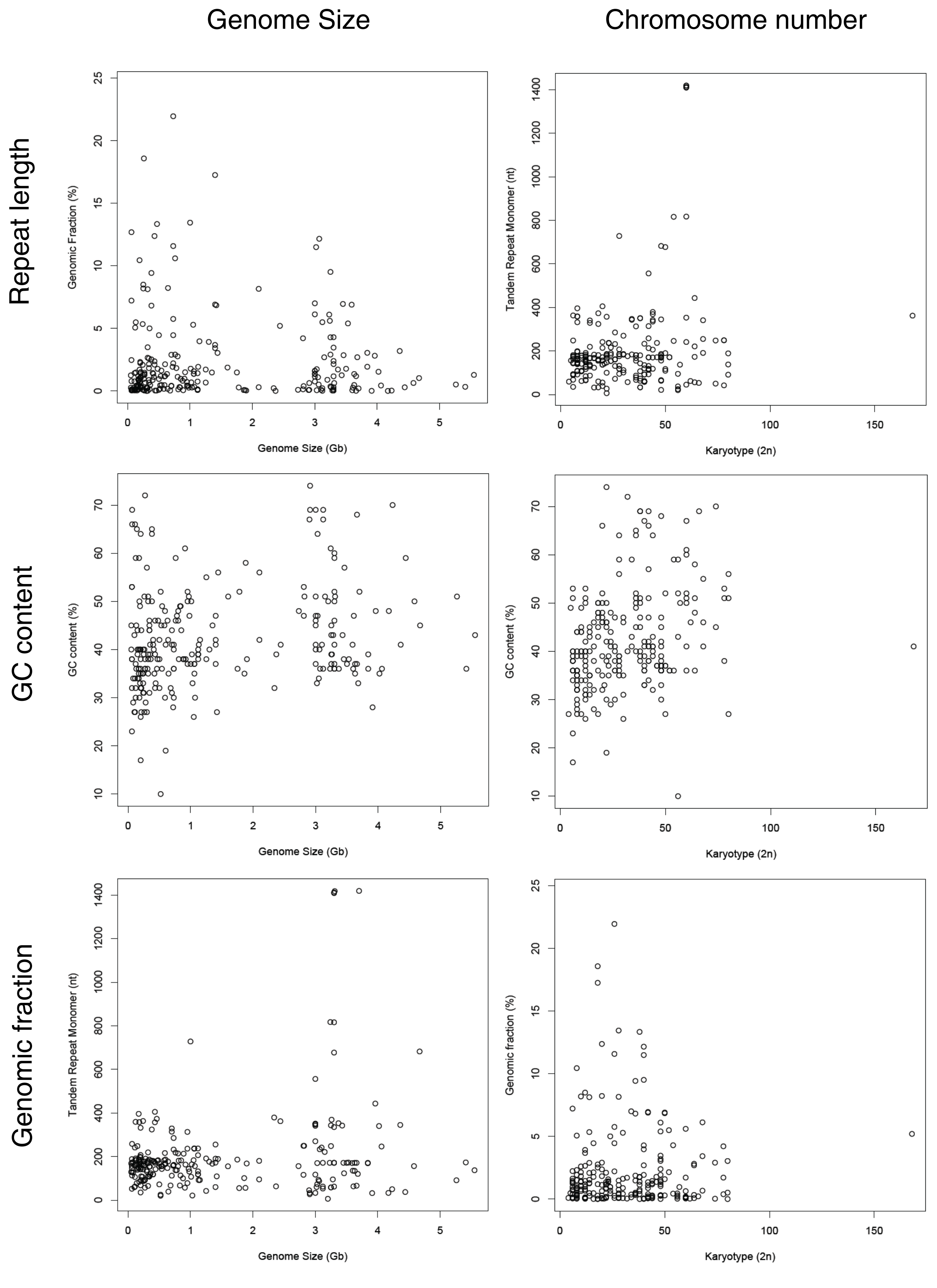

**Supplementary Figure S4** – Nile tilapia contains related tandem repeats that differ by a 29 bp indel.

```
tandem-71       TGTGTTTCAGGCGAGAACTCCATTCAAATGCATGTAATAGCGAGAAACGCACTGTCTCGC 60
tandem-519      TGTGTTTCAGGCGAGAA-----------------------------ACGCACTGTCTCGC 31
                *****************                             **************

tandem-71       CGAAATAAAGCGATTTTCACTAAGTTCATAAAGACAGCTGTAACTTTTGATAGAAGACTC 120
tandem-519      CGAAATAAGGCGATTTTCACCAAGTCCATAAAGACAGCTGTAACTTTTGATAGGAGACTC 91
                ******** *********** **** *************************** ******

tandem-71       GGACACACATATTTCAGGCTTGGCCTTATAGAATTCAGCATTTCTATGCTGGGGAAATAG 180
tandem-519      GGACACACATATTTCAGGCTTGGCCTTATAGAATTCAGCATTTCCATGCTGGGGAAATAG 151
                ******************************************** ***************

tandem-71       GTTTTGCAGCTGTTTGAGCTAAGATTTTCAAGTTATTCACAAAATGAAAACCCATAA 237
tandem-519      GTTTTGCCACTGTTTGAGCTAAGATTTTCA-GTTATTGACTAAATGAAAACC-ATAA 206
                *******  ********************* ****** ** *********** ****
```

```
>tandem-71
TGTGTTTCAGGCGAGAACTCCATTCAAATGCATGTAATAGCGAGAAACGCACTGTCTCGC
CGAAATAAAGCGATTTTCACTAAGTTCATAAAGACAGCTGTAACTTTTGATAGAAGACTC
GGACACACATATTTCAGGCTTGGCCTTATAGAATTCAGCATTTCTATGCTGGGGAAATAG
GTTTTGCAGCTGTTTGAGCTAAGATTTTCAAGTTATTCACAAAATGAAAACCCATAA
>tandem-519
TGTGTTTCAGGCGAGAAACGCACTGTCTCGCCGAAATAAGGCGATTTTCACCAAGTCCAT
AAAGACAGCTGTAACTTTTGATAGGAGACTCGGACACACATATTTCAGGCTTGGCCTTAT
AGAATTCAGCATTTCCATGCTGGGGAAATAGGTTTTGCCACTGTTTGAGCTAAGATTTTC
AGTTATTGACTAAATGAAAACCATAA
```

**Figure S5 - Relative ratios of short repeat (~680 bp) and long repeat (~1410 bp) in the five *Bovideae* species.**

| Species | Short repeat | Long repeat |
|---|---|---|
| ***Bos taurus taurus*** | **29%** | **71%** |
| ***Bos taurus indicus*** | **32%** | **68%** |
| ***Bos grunniens*** | **40%** | **60%** |
| ***Bison bison*** | **41%** | **59%** |
| ***Bubalus bubalis*** | **57%** | **43%** |

**Figure S6A**. Tabular comparison to figure 6A of sequence comparison. Empty cells show sequence comparison with less than 40% sequence similarity. The three letter abbreviation corresponds to the species in top-to-bottom order as shown in Figure 6A.

| | Gac | Oni | Mze | Mau | Lfu | Res | Pny | Nbr |
|---|---|---|---|---|---|---|---|---|
| **Gac** | 100 | | | | | | | |
| **Oni** | | 100 | | | | | | |
| **Mze** | | 66 | 100 | | | | | |
| **Mau** | | 62 | 94 | 100 | | | | |
| **Lfu** | | 67 | 95 | 94 | 100 | | | |
| **Res** | | 60 | 93 | 92 | 93 | 100 | | |
| **Pny** | | 74 | 94 | 95 | 97 | 93 | 100 | |
| **Nbr** | | | | | | | | 100 |

**Figure S6B**. Tabular comparison to figure 6C of sequence comparison. Empty cells represent sequence comparison with less than 40% sequence similarity. The three letter abbreviation corresponds to the species in top-to-bottom order as shown in Figure 6C.

| | Hvu | Ata | Bdi | Obr | Osa | Svi | Sit | Pha | Pca | Pvi | Zma | Zlu | Sbi | Spr | Mgi |
|---|---|---|---|---|---|---|---|---|---|---|---|---|---|---|---|
| **Hvu** | 100 | | | | | | | | | | | | | | |
| **Ata** | | 100 | | | | | | | | | | | | | |
| **Bdi** | | | 100 | | | | | | | | | | | | |
| **Obr** | | | | 100 | | | | | | | | | | | |
| **Osa** | | | | | 100 | | | | | | | | | | |
| **Svi** | | | | | 57 | 100 | | | | | | | | | |
| **Sit** | | | | | 59 | 98 | 100 | | | | | | | | |
| **Pha** | | | | | 62 | 68 | 67 | 100 | | | | | | | |
| **Pca** | | | | | 65 | 63 | 63 | 94 | 100 | | | | | | |
| **Pvi** | | | | | 41 | 44 | 45 | 56 | 55 | 100 | | | | | |
| **Zma** | | | | | 50 | 56 | 55 | 67 | 67 | 42 | 100 | | | | |
| **Zlu** | | | | | 49 | 54 | 53 | 65 | 66 | 40 | 98 | 100 | | | |
| **Sbi** | | | | | | | | | | | | | 100 | | |
| **Spr** | | | | | | | | | | | | | 91 | 100 | |
| **Mgi** | | | | | | | | | | | | | 87 | 84 | 100 |
