# Supplementary material for: Comparative analysis of tandem repeats from hundreds of species reveals unique insights into centromere evolution
Source: Genome Biol. 2013 Jan 30;14(1):R10. doi: 10.1186/gb-2013-14-1-r10 (PMC4053949; doi:10.1186/gb-2013-14-1-r10)
Supplement: Additional file 1 — Supplemental methods. [file gb-2013-14-1-r10-S1.PDF]

Supplemental Methods

Patterns of Centromere Tandem Repeat Evolution in 282 Animal and Plant Genomes

Daniël P. Melters*[1,2], Keith R. Bradnam*[1], Hugh A. Young[3], Natalie Telis[1,2], Michael R. May[4], J. Graham Ruby[5], Robert Sebra[6], Paul Peluso[6], John Eid[6], David Rank[6], José Fernando Garcia[7], Joseph L. DeRisi[5,8], Timothy Smith[10], Christian Tobias[3], Jeffrey Ross-Ibarra[9], Ian F. Korf#[1] and Simon W.-L. Chan#[2,8]

Notes:
1. All (candidate) centromere repeat monomer sequences can be found at the end of this document.
2. All Perl scripts can be downloaded from the Korf Lab website: http://korflab.ucdavis.edu/Datasets/Centromere_data
3. Accession numbers for sequences used in this paper can be found in Supplementary Table S1.

Bioinformatics pipeline for tandem repeat clustering

In this Methods section we will use results for gorilla (*Gorilla gorilla*) as an example of how each step works.

Trace Archive Data files

For Sanger reads, we downloaded up to five randomly chosen Trace Archive sequence files along with their supporting ancillary files for each species from the NCBI Trace Archive (http://www.ncbi.nlm.nih.gov/Traces). E.g.

fasta.gorilla_gorilla.002
fasta.gorilla_gorilla.004
fasta.gorilla_gorilla.007
fasta.gorilla_gorilla.011
fasta.gorilla_gorilla.017
anc.gorilla_gorilla.002
anc.gorilla_gorilla.004
anc.gorilla_gorilla.007
anc.gorilla_gorilla.011
anc.gorilla_gorilla.017

If for any species, fewer than five sequence files were available, we used all available sequences. Each file typically contains 500,000 sequences.

***Gorilla gorilla* input: 2.5 million sequences**

We then filtered these sequences as follows (using information in the ancillary files for steps 1–3):

1) Ignored any sequence which wasn't flagged as either Whole Genome Shotgun (WGS) or Whole Chromosome Shotgun (WCS)
2) Clipped sequences for quality and/or vector contamination
3) Ignored sequences with clipping information that referred to coordinates longer than the actual sequence
4) After clipping, removed any sequence which contains >5% Ns
5) After clipping, removed any sequence which was <100 nt
6) Ran DUST filter on resulting sequences
7) Removed sequences that contained >5% Ns after DUSTing

Resulting number of sequences in *Gorilla gorilla* after filtering: ~2.3 million

Tandem Repeats Finder (TRF) parameters

A command-line version of TRF (version 4.04) was run using the following parameters:

Match = 1
Mismatch = 1
Indel = 2
Probability of match = 80
Probability of indel = 5
Min score = 200
Max period = 750

From the output of TRF, we we only kept tandem repeats that contained a minimum of 2 repeats (TRF will identity tandems from less than 2 complete repeats), and which had a minimum length of 50 bp. The processed TRF output consists of a FASTA file containing the *consensus* repeat sequences that TRF produces.

Number of tandem repeats in *Gorilla gorilla* after running TRF: 69,041

Clustering of tandem repeats

Clustering similar tandem repeats was a CPU intensive step and not practical for those species in which we had identified tens of thousands of different tandem repeats. At this point we chose to select a random sample of sequence reads for each species, and then find all of the corresponding tandem repeats that we had already identified in those reads. For this step we chose up to 200,000 reads from each species (fewer if tandem repeats were more common in that species).

Number of reads randomly selected in *Gorilla gorilla*: 100,000
Number of tandem repeats present in those reads: 3,489

We chose to sample reads first, in order to record their lengths for a calculation that occurred in a later step. At this stage we also tracked how many repeat units were present in each tandem repeat that occurred in the selected reads, along with the total length of the tandem repeat array.

We then took the monomer units from the sampled tandem repeats and made tandem versions of them. Next, we used WU-BLASTN to search each monomer repeat sequence against a database of all the tandem-ized version of those repeats (WU-BLAST settings: M=1, N=-1, R=3, Q=3, W=10). We only retained matches that had at least 75% identity.

Number of BLAST HSPs in *Gorilla gorilla*: 12,509,094

At this point we processed the BLAST output in two ways. In 'global' mode, we only retained BLAST matches between repeats if the match spanned the majority of the monomer repeat length. In 'local' mode, we did not require this retriction and so partial matches of a repeat monomer to a tandem repeat could occur.

For both global and local mode, we then found the tandem repeat that had the highest 'tandem repeat mass'. This was calculated as follows:

1) Count how many matches each repeat has to all other repeats (excluding self-matches)
2) For each match, multiply the length of the matching repeat by its copy number in the read where it was originally detected
3) Sum these multipled lengths to produce 'tandem repeat mass'

All repeats that matched the individual repeat with the highest mass were then removed from the analysis (along with the high mass repeat) and the process was repeated. Each iteration of this process defines a cluster of related repeats. In local mode, clusters grow bigger in size and 'swallow up' more of the repeats that match only partially.

For each cluster we can record:

1) Number of sequences
2) GC% of sequences
3) Length of representative cluster member
4) Mean pairwise identity between sequences in cluster
5) Cluster 'depth' – total number of matches to top hit in cluster, including matches to the matches
6) Cluster 'mass' – the summed tandem repeat mass of all sequences in cluster
7) Genomic fraction – the tandem repeat mass as a fraction of the total length of the sample sequences

We typically focus our analysis on just the top global and top local cluster, and these typically account for the majority of the total amount of tandem repeat mass. Exceptions

occur when there is higher order repeat structure. E.g. the results below for *Gorilla gorilla* show that — in global mode — the top cluster finds the known centromere repeat (length 171 bp) and this accounts for ~13% of the sample reads (inferred to be representative of the genome as a whole). However, the second cluster finds a similar number of sequences which are double the length of the top cluster, and which are presumed to represent the same sequence as the centromere repeat, but in a tandemized version. In contrast, the top local cluster contains a mix of both 171 bp and 340 bp sequences and therefore accounts for a higher genomic fraction (25%).

Example cluster output for *Gorilla gorilla*

```
Summary for Gorilla gorilla tandem repeat analysis
100000 reads, 3489 tandem repeats, 71072791 bp, 0.4232 GC

Repeats (global mode) - using mass threshold %0.1
Cluster n       id#     length  gc      mean    stdev   depth   mass    frac
0       1230    50980   171     38      87.10   6.00    5399    920589  0.012953
1       1057    61930   340     39      90.06   5.17    2437    827458  0.011642
2       745     72354   70      54      99.16   1.25    7716    540129  0.007600
3       22      33954   171     39      87.29   5.44    100     17016   0.000239
4       20      47128   171     38      85.17   4.26    87      14891   0.000210
5       16      14196   342     34      79.77   4.19    37      12680   0.000178
6       14      6017    76      50      93.72   3.08    123     9322    0.000131
7       12      27715   349     53      85.31   3.27    25      9073    0.000128
8       16      33244   96      37      94.10   3.76    91      8813    0.000124
9       12      58454   64      37      96.93   2.44    130     8316    0.000117
10      11      36485   279     53      88.40   4.60    29      8226    0.000116
11      8       728     341     36      79.59   3.54    17      6125    0.000086
12      9       56545   209     52      93.46   2.28    28      5996    0.000084
13      9       35159   98      69      97.96   1.32    54      5299    0.000075
14      7       32585   168     58      89.05   5.39    25      4352    0.000061
15      5       70673   84      38      89.00   3.08    44      3779    0.000053
16      5       14582   170     37      88.28   7.16    21      3663    0.000052
17      8       62437   161     40      84.42   4.82    22      3569    0.000050
18      5       45011   298     51      80.19   3.19    11      3532    0.000050
19      6       28593   140     54      95.37   2.31    24      3443    0.000048
20      8       33970   125     35      85.75   3.69    21      2763    0.000039
21      5       49512   175     79      85.47   5.73    13      2421    0.000034

WARNING: Top clusters are similar in size. Cluster #1 has a tandem repeat mass within 50%
of cluster #0
NOTE: 193 clusters are not reported due to having a tandem repeat mass less than %0.1 of
cluster #0,
or the cluster contained less than 5 sequences


Repeats (local mode) - using mass threshold %0.1
Cluster n       id#     length  gc      depth   mass    frac
0       2339    17934   171     36      8026    1787776 0.025154
1       787     15282   348     50      7849    569187  0.008009
2       54      33244   96      37      298     31018   0.000436
3       24      62336   151     46      157     16369   0.000230
4       22      17637   200     58      118     14271   0.000201
5       18      47128   171     38      68      13270   0.000187
6       23      32502   127     78      79      11472   0.000161
7       17      45011   298     51      48      11028   0.000155
8       14      19227   339     39      48      10418   0.000147
9       15      35159   98      69      76      8880    0.000125
10      7       36688   63      42      47      4469    0.000063
11      5       70673   84      38      44      3779    0.000053
12      5       31667   84      67      21      3451    0.000049
13      6       21535   161     69      37      3283    0.000046
14      5       72797   71      60      30      2335    0.000033
```

```
NOTE: 107 clusters are not reported due to having a tandem repeat mass less than %0.1 of
cluster #0,
or the cluster contained less than 5 sequences
```

Graphing of results

We produced XYZ graphs to further study the sequences within the top clusters (X-axis = repeat length, Y-axis = repeat GC content, Z-axis = genomic fraction). Multiple peaks with different length, but the same GC percentage would be separated by steps less than 50 bp. These peaks would represent various multimers of shorter repeat than the cutoff of 50 bp. In these instances, TRF and the subsequent steps were re-run but with a minimum repeat length of 20 bp. This was done for ~40 species.

Sequence Read Archive files

For Illumina and 454 reads, we downloaded either *.lite.sra or *.fastq.bz2 files for species from the DDBJ Sequence Read Archive (http://trace.ddbj.nig.ac.jp/DRASearch/), e.g.

Species	: *Gorilla gorilla gorilla*
Submission	: ERA013840
Study	: ERP000004
Experiment	: ERX007929
Run	: ERR019516
File	: ERR019516.lite.sra (2.1 Gb)
Unpacked files	: ERR019516_1.fastq and ERR019516_2.fastq

For each FASTQ file two were made: one with 1.000.000 randomly selected reads and one with 100.000 randomly selected reads. This means that per assembly run either 2.000.000 or 200.000 reads were used.
The read length for Illumina data ranged from 36 to 152 bp, whereas for 454 data the read length ranged around 200 bp.

Because Ilumina and 454 reads are often too short to contain at least two copies of a tandem repeat, an assembly step was necessary to find tandem repeats.

PRICE parameters

The command-line used for PRICE (version 0.6) was:

-fpp Gorilla_gorilla_100k_1.fastq Gorilla_gorilla_100k_2.fastq 475 90 (input files)
-nc 25 (number of cycles)
-mpi 85
-MPI 95
-tpi 85
-TPI 95
-logf Gorilla_gorilla_run1 (log file)
-o Gorilla_gorilla_run1.fasta (output file)
-picf 20000 Gorilla_gorilla_1M_1.fastq 500 2 25 (seed file)

For a detailed manual go the PRICE website (http://derisilab.ucsf.edu/software/price/index.html) or on the command-line type "PriceTI -h"

Per cycle a FASTA file was created containing the assembled contigs. These FASTA files were fed into the TRF pipeline as described earlier for Sanger reads.

Tandem Repeats Finder (TRF) parameters

A command-line version of TRF (version 4.04) was run using the following parameters:

Match = 1
Mismatch = 1
Indel = 2
Probability of match = 80
Probability of indel = 5
Min score = 200
Max period = 2000

The only difference between the TRF parameters for Sanger data vs Illumina or 454 data is that the maximum tandem repeat length is not 700 bp but 2000 bp, the upperlimit of TRF (Benson, 1999).

Genomic fraction determination of PRICE derived tandem repeats

Tandem repeats derived from PRICE contigs were doubled in length. 2.000.000 reads (both 1.000.000 files were used) were aligned to the doubled tandem repeat monomers with WU-BLASTn. Only the top hits (hspmax = 1) was used for determining the genomic fraction. The minimum BLAST score was dependent on the length of the reads. The number of reads that significantly aligned to the tandem repeat were counted and the fraction of these reads over all the reads determined the genomic fraction. The average of each file was used as the genomic fraction for that specific tandem repeat. The most abundant tandem repeat was predicted to be the candidate centromere tandem repeat.

Pacific Biosciences data

PacBio reads were processed the same way as Sanger reads, as described earlier, with the exception that the maximum tandem repeat length was set to 2000 (Max period = 2000). Please note, that only the insert sequence was used for analysis, not the complete PacBio read. Because two loops are ligated to a double stranded DNA fragment, the polymerase can read multiple times over the same DNA molecule. For determining tandem repeat structures, only a single pass over the dsDNA molecule will suffice.
For the grasses (*Panicum virgatum, Panicum capillare, Zea mays, and Zea luxurians*) the sequences longer than 1,000 bp were used. For the bovine species (*Bos taurus taurus,*

Bos taurus indicus, Bos grunniens, Bison bison, Bubalus bubalis) sequences longer than 3,000 bp were used. This was to guarantee that the genomic fraction would not be diluted with sequences which would be too short to contain at least two copies of the described centromere repeat sequences (see Supplementary Table S2).

Supplementary Table S5 - Number of PacBio reads per species.

Species	>1 kbp	>2 kbp	>5 kbp	>10 kbp
Panicum virgatum	27702	15699	2124	3
Panicum capillare	14624	6596	216	0
Zea mays	137989	43145	6829	175
Zea luxurians	87448	25063	2031	51
Bos taurus taurus (Hereford)	nd	59294	16319	593
Bos taurus indicus (Nellore)	120588	54463	7408	35
Bos grunniens	nd	74043	9014	263
Bison bison	nd	107023	29945	1093
Bubalus bubalis	nd	158768	16941	501

Plant material and growth conditions

Seeds from the inbred line B73 of *Zea mays* were provided by the Hake lab (PGEC, Albany, CA). *Panicum capillare* seeds were obtained from Tom Juenger (UT Austin, TX). Foxtail millet (*Setaria italica*) seeds were obtained from the National Genetic Resources Program (NGRP, Beltsville, MD), whereas dihaploid switchgrass plants (*Panicum virgatum*) were already available (Young et al., 2010; Young et al., 2011). Seeds from all species were germinated in appropriate soil and plants were maintained in the greenhouse at 21-28°C, under supplemental light (16:8 photoperiod), watered as needed, and fertilized weekly with general purpose 20-20-20 fertilizer.

Switchgrass (*Panicum virgatum*) DNA isolation

Switchgrass (*Panicum virgatum* AP13 (tetraploid)) genomic DNA was isolated using a modified protocol from Chen and Ronald (Chen and Ronald, 1999). In summary, fresh leaf tissue was ground thoroughly in liquid nitrogen and the powdered tissue was added to 4.0 mL of extraction buffer (1.42 M NaCl, 100 nM Tris-Cl (pH 8.0), 2% (w/v)

polyvinylpyrrolidone (PVP-40), 20 nM EDTA and 2% (w/v) CTAB), 5 mM ascorbic acid, 4 nM diethyldithiocarbamicacid (DIECA), and 0.2 mg/mL RNase (DNase free). Next, the solution was incubated at 65°C for 15 minutes, after which 3.0 mL of chloroform/isoamyl alcohol (24:1 ratio) was added. This was centrifuged at 3,500 rpm for 10 minutes at room temperature. The aqueous phase was transferred to a new 15 mL tube and 0.7 volumes of isopropanol was added, followed by 30 minutes of centrifugation at 3,500 rpm at room temperature. The supernatant was removed and the pellet was washed with 5 mL of cold 70% ethanol for 30 minutes on ice. All ethanol was removed and the pellet was airdried overnight. The next day the pellet was resuspended in 100 μL TE buffer and stored at 4°C overnight. The sample was centrifuged for 10 minutes at 3,500 rpm at room temperature and the supernatant containing DNA was transferred to an eppendorf tube.

Fluorescence in situ hybridization (FISH) experiments

Mitotic chromosome spreads were generated following a protocol by Zhang and Friebe (Zhang and Friebe, 2009; Zhang et al., 2010) with a few modifications. Actively growing root tips were excised from greenhouse grown plants, pretreated in ice cold water for 18-24 h, and then fixed in 3:1 ratio of 95% ethanol and glacial acetic acid at 4°C, overnight. Root material was either used immediately for slide preparation or stored in fixative at -20°C for up to several months. For slide preparation, the root tips (0.5-1.0 cm) were washed twice for 5 min each in 0.01M citrate buffer and digested in an enzyme mixture of 50 mg/ml Onozuka R-10 cellulase and 30 mg/ml Macerozyme (Phytotechnology Labs, Shawnee Mission, KS) at 37°C. Digestion times varied from 30 min to 2.5 h, depending on the thickness and degree of lignification in the root tip. Softened root tips were then washed for 5 min in 0.01M citrate buffer and transferred to a slide. Forceps and a scalpel were used to carefully excise the white tissue just behind the root cap containing actively dividing mitotic cells. All other root tissues were removed and the remaining cells were macerated in a few drops of 1% acetocarmine stain. A coverslip was placed over the stained tissue and even pressure was applied to generate mitotic chromosome spreads. Slides were viewed under phase-contrast microscopy to identify spreads optimal for use in FISH analysis.

Plasmid vectors containing a single copy of each repeat sequence were synthesized by Bio Basic Inc. (Ontario, Canada) and used as probes for FISH analyses. Plasmid vectors were labeled with either digoxigenin-11-dUTP or biotin-16-dUTP using a nick translation protocol, in accordance with manufacturer instructions (Nick Translation Kit; Roche Applied Sciences, Indianapolis, IN). Hybridization and post-hybridization wash procedures were performed as previously described (Jenkins and Hasterok, 2007). Probe hybridization signals were detected using anti-digoxigenin (dig) conjugated FITC (green), anti-dig conjugated Rhodamine (red), or Streptavidin conjugated Rhodamine (red) antibodies (Roche Applied Sciences). Dual probe labeling of chromosomes was

conducted using non-competing dig and biotin antibodies to allow for simultaneous red and green imaging. Chromosomes were counter-stained with 4',6-diamidino-2-phenylindole (DAPI).

Digital images were recorded using an Olympus BX51 epifluorescence microscope (Olympus Corporation, Center Valley, PA) with a DP70 CCD (charge coupled device) camera and suitable monochrome filter sets (Chroma Technology, Rockingham, VT). Images were processed using GIMP 2.6 (GNU Image Manipulation Program) for Linux. Analysis of FISH data was conducted by overlaying a probe signal image on top of the corresponding DAPI stain image. Adjustments were made to the transparency of the top (FISH signal) layer to demonstrate signal and chromosome alignment.

Repeat variant sequences:

Variant A (*Zea mays*):
5'-
CTTTAGGTCCAAAACTCATGTTTGGGGTGATTTCGCGCAATTTCGTTGCCGC
ACGTCACCCATTCCGAAAACGGGTATCGGGGTGCATACAAAGCACGAGTTTT
TGCCACCGGAACAATTTCTTCGTTTTTCGCAACGAACATGCCCAATCCACTA-
3'
Variant B1 (*Panicum virgatum*):
5'-
TTCGTTGCGAAAAATTCCGATGCGACTTCATGGCACGAACTTTTGCATTAAT
TGCACCAGTTCAGCCCATTTTGCACCGAGTTTCCTGAAGTAACGAAACGATGC
CAAATGCACCCAAACACTACGAAACGCACCAAAACATGAGTTTAGGGTCCAA
TGGGGTGGATCGGGTGCG-3'
Variant B2 (*Panicum virgatum*):
5'-TTCGTTGCGAAAAATTCCGACGCGACTTCGTGGCACGAACTTTTGCACTAG
TTCGGCCCGTTTTGCACCGAGTTTCGTAAGGCAACCAAACGGTCCCGAATGC
ACCCAAATAGTACAAAACGCACCAAAACGTCAGTTTAGGGTCCAATGGGGTG
GATCGGGTGCG-3'
Variant C (*Panicum capillare*):
5'-
TTCGTTGCGAAAAATTCCGACGCAACTTCGTTTAGCGAACTTGTGCGTTAAT
GGCACCAGTTCGGCCCGTTTTGCACCGACTTTCGTGCAGTAACGAAACGGTC
CGAAACGCCCAAAAACATGAGTTTTGGGTCCAATGGAGTGGATCGGGTGCG-
3'
Variant D (*Setara italica*):
5'-TTCGTTGCGAAAAAATCCACCCGAGTTTCGCTACCCGGAAATAGTGCATTC

GGGTGCCGAAATGCACCCGTTTTGCATCATTTTTCGTGCCGGAACCGAATGCC
CAAAAACACTCCCAAACATGTTCTAGTGTATATTTAGGAAGATTGCATGCG-3'

All (candidate) centromere repeat monomers for the 282 species in this study:

```
>Cynocephalus_volans
CATTCTGCTGCTTCCTCCCTTAGAGTTAAACTAGCTTCTGAAAGCTCAACCTCCGGGGAG
TCTGTTTGGCAGAGATATAGAACACACTATTCCAAGACTGTTGGAAGGCCTTTGCAGTGT
CATTTCGCATCTAATTTCCCAAACCAAACTGCTTGCAGCTCTAACTCCCTTCTAACTACC
ACTTCTTCGGTTCAGTGAGATATGCTGGATTCTAAACGGAGAAGCTTCTCTGACGCTTTT
TCACAGAGATTTTGAAACTGAAATCTCCCATGCCGTTTCCACCATAGGCAGCAATGGGCT
TCCCCATAGAACTTGCCAACCTGAATTCCTACAGGCTACATGTGAGAGGCA
>acropora_millepora
TACTTTTTGCAACATTTTTCTAAAAATTGGGTCAAAACCCTAGTGCACGGTACTTTGCAC
AAAAAGTTGCTTATCTCGAGGAGATCGACAAGTTTTGGTGGTTTTTCAGCAAATGCCC
>acropora_palmata
TGGTGAAAAACCACCAAAACGTGTCGATCTCCTCGAAATAAGCAACTTTTTGTGCAAAGT
ACCGTGCACTAGGGTTTTGACCCAATTTTGAGCAAAATGTTGCAAAAAGTATGGCATT
>acyrthosiphon_pisum
AAACGTACACCAAAACTTAGAGTAGATTAAACCCTATCTTTTGGTAAGAAAATTATGAAA
AAATATTAAGTATTGGAGAAACGGGACGGGATTCCACCGCCACAGGTGCTGAAATATAGT
CCAAATTGTAGACGGGACGAGCTGTAGTTAATTCAAAAATGAACTAAGACTTATG
>aedes_aegypti
TTTTGGCAGAGTGAATTGACAAATCCAGCACACGGAAAGTTAGCTGGGATCCAGACGAAG
CGATTGATGCTAAGAAATCGAAAATCCGTTGATATTTACTCAACTTTTTAGGAAATCTAC
GGATTTTTATAATTTCATTGGATTTTCAAGCATGGGTCCCACTAAAAGTTGGAGGCTAAG
G
>ancylostoma_caninum
TATCGATTTATCGATTTTATTGATTTTTCGCTATTTTTGCTCAGGGGTCGGTGGGGACAT
GTCTACTGGGGTTTCTCAATACGCTGAACACGAATATGGCAACCGCTTCGCACGAAAA
>anolis_carolinensis
GACCCGCCGGGCTTGGAAAATGATGAAATTTTCCAGAAAAAGTGACCTCTGCCAAATCTG
AAAAACTTGCCAAATTTTCATTTTTCT
>anopheles_gambiae
CTATTTGAATGGCCTTGAAATTTGATGTTGAATTTTGTTGCCATCTATTATCGTTGTTAC
GGCCATGTTTCATCCAAATGAAGCCATCTTGCA
>anopheles_gambiae_M
AAATTCAACATCAAATTTCAAGGCCATTCAAATAGTGCAAGATGGCTTCATTTGGATGAA
ACATGGCCGTAACAACGATAATAGATGGCAACA
>anopheles_gambiae_S
ATGGCCGTAACAACGATAATAGATGGCAACAAAATTCAACATCAAATTTCAAGGCCATTC
AAATAGTGCAAGATGGCTTCATTTGGATGAAAC
>apis_mellifera
CGCTATGGTCGTCCAAAGAAGTGGAAATTTTAGAATCTTTAAAATAGTGCATTATTAAAA
CGGCTGTATCTCCGAAGATAAGAGAAAAAAAATACGATTTCCGCAGCTCGTTGCAAGCGGG
AAAGATTGCTCTTTGACCTCATGTTTGGTTGGGTTCTGGACGACAAGCGGTTCATA
>aplysia_californica
ACAAGCATTCACGCGAGACAGAATGCAAACTAGCATTCAAGCAAGAAAGAATGCAAACGA
GCATTCAAACAAGACAGAATGAAAGCATGCATTCAAGCAAGACAGAATCCAAGCAAGTAC
TGAAGACGGAGAGAATGCAAATATGCATTTAAAATAATCCGAATGCAA
>aquilegia_coerulea
CTCGGTTTTCGGTCCGAACTTGGAAATGGAGAAACTCACTTGTTTTGGACTCTAAAACCT
CAAGATCGACAAGACTTAGCCGGGACGTTTTAAGGCCAATACAGAACATTTGCACCATTG
GAACGGGATTTTGTCCGGACATTCGGCTTGGTATGTACTCGATTTTTGTTCCGAACTTAT
```

GAGTGGACGAAACACACATGTTTTCGCATGGAAAACGATACACCTAAAAGACTAGCCACT
CCATTACTTAACACGGGGCGTTCTAAGGGGAAAAACAACGTTCCGTCCGTAAAAACGTGA
TTTATGAACGTCCGTTCGGTCGGCATTTA
>arabidopsis_lyrata
CGGGATCCGGTTGCGGCTCTAGTTCTTATACCCAATCATAAACACGAGATCTAGTCATAT
TTGACTCCAAAAACACTAACCAAGCTTCTTATTGCTTCTCAAATCTTTGTGGGTGTGGCC
GAAGTCCTATGAGTTTTCGGTTTTGGAGCTTCTAAACGGAAAAACACTACTTTAGCTTT
>ascaris_lumbricoides
ATTGAATGGTCACCAGATCATTGGTGAGTTACGACTCATGATGATTATTCTATATCACTA
AGTTAAT
>ateles_geoffroyi
ACCCTTTCTGTGCAGAGATGCAAAACTGTCATTTCCAGCCAAATCCAGGCACGTCAGAAA
AAGCAGTAATAGTGCGTCTAAAACACAGAAGAAACGCATCATTCAAACTGCTCTGCTGTG
TGTTCGTTTCAACTAAGGGAGCTGAAGCGGAGTTTCATTCAGCGAGTTAGCAACACGTTT
CTTCTAGGAACTGCGTTTGGATTTTCCAGAGCGAAAGGGAGCATTTGCTGTACAGAGGAA
TATCTCGCTCTAAAACCAAAACGGAGCAATCTAACTGAATGCTTGTCAATGTGTGCATTC
AACTTACAGAGTTAAACTCGTGTGTGTTTGCAGCAGTTTAGAA
>biomphalaria_glabrata
CAGCTACTTCCTTGACAATATCCCAGAAGTTATCTTGTTGCTCGGCACTTCTGCCAACGA
GAAGGGGAAGGGCTACTC
>brachypdium_distachyon
GGATTCGCTAGGCATGGTCCCTCACTAGGACGGACAATTCGGGCAAATCGGAGTCGGTGC
TAAACTTTGATCCGGAACGTGTCCATTTGAGGTCCTAGGATGAGCACACCAAACAAAGCT
ACTCATCGAAGCGAAACAAGACTAGACCACTTGTAC
>branchiostoma_floridae
ACCAAGCTTGCCAGGCCGGAACTTGAAGCCTAAGCTACAATCGCGTTATTAAGTCACCCT
CCTACCCGGTGACGTGACAGGCGGTGTTTCAGAAAAAGCAGCTAGCGGTTGCGCCTAAAC
AGGGTCGGTATACTCAAGAATATCTCGAGAAGGAAGTATCCCAACATTTTGCGGTTTTCA
TCTTTCCATTCCTTGCTAAGGAACCTTTCCAACCAT
>brassica_oleracea
TCTCCACTACTTTATGTATCCAAATACAGCTTCTTACATCGCGATTCATCCTGGTTTGAT
CAGAATGACGAGGAAGTTGTCATATTCCCAAACAGGAAAACTGGGATCACCTGATTTGAA
AGTGGGATAACTTCTTCATCCTAACTCCTATGAGATTTATTCAACTTCCTGGTGAT
>brugia_malayi
TTCATTAACTCATACCATTTCTCTACAGATATAACAATATCACTAGAAGACATTTTGATT
AA
>caenorhabditis_briggsae
AATTTATCACATACGAGTTTTGTGAAAAGGCAGAACAAGCAAAGTTTAAAACAATGATAT
TATTATGGCTAATATTTTACAAAAGAATTTGTGTGAATTGGCTCATTCAGAAAAAAGTTG
GAAGCCAAAATCTCGATTTTAGGGCTCAAAAACCGATTTTTCG
>caenorhabditis_japonica
CGTTCAATTTTCAAAACACTAAGGCTCGGCCAATTTTTGATGAAAATTGCTCGTTTTTGG
ATCAAAATGAAGCTTTCAACCTGATTAATCTGATTATGGTTCGTATTGCGCATTCCAATC
GTGTTGTATCATCAAAACACAAGTCTGTATTCACCGAACTGTCTGAGTCTCTTCAAATAG
AACTGTGTTGG
>caenorhabditis_remanei
CCTTGAAAGCACAGTCTTGGAAGTTCTCATATAGACTTTTGTGAAACTTCAAGCGTGTGC
GGCCTTGTTCAGTTTGAACGTTATTGGCTGAAAATTGGCACAGTAGTAGACAATCCATTG
CCAATTCGAATGCACTTTACTGTTTTCAGATATCTCATCCAGAAAAAAAGTTAGAGACAA
TACA
>callithrix_jacchus
AGAGTGGAGCACGTTCCTTCTAGGAGCTGCGTTTTGCTATTCCAGCGTGAATGGGAGTAA
ACGCTGTATTGAGAAATATCTGCTTCTAGAACCAAAACGCAGCTACCTAACAGAACGGTC
TTCAATGTGTGCATTCAACTTACAGAGTTAAACTGATATGCGTTTGCAGGAGTTTGTAAA
CCCTTTCTGTGAAGCAGCGGAAAACGCATTTTTCCAGCCAAATCCAAGCATTTCAGAAAT
AGCTGTAAATCTCCGTCTAAAACACAAAACGAACGTATCCTTCAAACTGCTCTCCTGTGC
GTTCGCTTAACTAAGGGAGTTGAATCCGCGTTTAGATTCAG

>callorhinchus_milii
CCGGTGGCTCAATACCGTCACTGCAGCCGCTATAATGAAGCGCAGCAACAGCGCCGCCCG
GTGGCTCAATACCGTCACTGCAGCCGCTATAGTGAAGCGCAGCAACAGCGCCGCCCGGTG
GCTCAATACCGTCACTGCAGCCGCTATAACGTAGCGCTGAGCAACAGCGCCGCCCGGTGG
CTCAATACCGTCAGTGCAGCCGCTATGTTGAAGCGCAGCAACAGCGCCGCGCCGTGGCTC
AATACAGTCACGGCAGACGCGATAGTGAAGCGCAGCAACAGCGCCGT
>canis_latrans
TGGATACTTGGTCCACATCTGGGCCTCACTGGAACTACGTGACTCTGGTCCATCTCTGGG
CCTCACCCTGACTGGTGACTCTGGTCCCCACCTGGGCATCACTTGGACCTGGTGACTCTG
GTCCTACCTGGGCCTCACCTGGACCTGGTGACTCTGGTCACCAACTGGGCCTCACCTGGA
CCTGGTGAATCTGCTCCCACCTGGGCCTCACCTGGAACTTGTGACTGGTTCCCACCCGGA
CCTCACTGGACCTGGTGAATCTGGTCCCACATGGGCTCATAGGACC
>capitella_sp
TATTGAATACCTAGAGAATCGATTCTGTGAGCTTATTTCATTCGAACAGAACCATCTGAA
GCAATTCGTCTTGACTTCAATGCATTACAGAATCAATTCTGTTGAACTATAAGATCCACT
ACAGAATCAATTCTGTGAGCT
>capsella_rubella
ACAATGAATTTGATTGATTACAAGTGCTAGAGATGCATGAAGAATGTTTAAAAGTGAGAA
GAAAGACTTGTTGATATTTGGTCCCAAATGGGATAAGAACCCAAAACCATTGCTTTGAGG
CAGTGAATGGCTTGTATAAGTTATTTTGGGTTAGAATATGTTATAAT
>cavia_porcellus
TTTCACCACACATGCACATTGTAAAATACATGAAAGAATTCACACTGGAGAGAAACCCTA
TGTATGTAAGCAATGTGGGAAAGC
>chlamydomonas_reinhardtii
AATGCAGACTCGAGCAGGGAGCCATGTTGCCAGCCCTCACAGTGCCTTCAGTGCCCCTGC
ACGCCTGGACAAGGCGGGTGGGGTCCCTGCCGCCCAGCCATCACCAAACACCCCACCTGG
CACAACCACCCTTGTGCACTGTTGTTTCACATTTTCATATGTGCATGTTGCCTGACCTAT
TTGC
>choloepus_hoffmanni
CTGGGCTGAAAAACAACTTTTTTGAAAGCAGTCACTTGGAACTCTGTGAAAGCAGTATGT
TACACAGTTTTGAAAACTAAGTTGAATACAGCTTTCATTCACTTCTATGTGTTTTCC
>ciona_intestinalis
GTGTAAAACGACCTAGGATGGTCGGGTCGGCAATCGGTCGACCTCCGACCGAGCGACTTC
GATCACGACCTCGGGGATCTGGCTCCGCGGCAAGGGGCACCTCGGCACCCCGCGGAGAGC
TCGGGAAGCCCGGCGCAATTGTACAACGGCCGGAGGCGGACAGCTTCCACCACAACGAAG
CATACGTCGGGCCGACGTTTTCGCTCA
>ciona_savignyi
CTTTAAAACGCTAAAGGAACGTGGTACTAGGTCGGTTCTACGACCTTAGCGTGGCACGAC
TCGCCCAACTCGTCGCCGGCACGAGAAAACGAAACTCGCGCGCGCCGGGAGAAAATTCGA
ACGCGAAAATTAGGGGGGTAGATCCGCGCGCGCGCCGAAACGGCCCCTTTGCCGACGTCG
CATCG
>citrus_clementina
TCCGTGTGCCAAAAATAGGCCGCGGGCAAAGCCGCGCCCAAAAATAACCGCCCGAAGGCC
GGGGCGCCAAGTTCGTCCAGCGGAAAAAGGCCACAAAAACGGGTGGGCTATAGCCTTGGG
GGGTGGGGCTGGCCAACTTCGTCCGCCGGACTCGGAATGCCGCGAGACTTTGCGAGGGGC
C
>citrus_sinensis
GGGCGCGGCTGTGCCCGTGGCCTATTTTTGGCACACGGAGGCCGCCCGCAAAGTCTGGGG
CCATTCCGAGTCCGGCGGACGAACTTCGCCCACGCCCCCCACCAAGGCTATAGCCCACCC
GATTTTTGGCCATTTTTCCGCTGGCGAGTCTTGGCGCCCCGACCTTCGGGCGCTCATTTT
T
>culux_pipiens
TACATTTAATTGAGATCCGGCTTCAAAAAAGTACATAAATATCACTTAAGTGGTCATAAC
TCGAGACAGGGTTGCCAGATCTTCAATGTTGTGGACTTGTTGGAAAGGTCTTTTGATTAC
CTAACTAACGATGGGTCGGATGATGGATCCGGACATCATTTACA
>cynocephalus_variegatus
CTCCCTGGAGGTTCAGCTTCTGCAAGCCACTTTAACTTTAAGAAATGAGAGAGCAGAATA

TGCTTCTCACATCTAGCATGCAGGAATTCAGGTTTGCAAGTTCTATGTGAAAGCCCATTG
AAGCCTATGGGGGAAACGGCTTCTGAGACTTCTGTTTCAAAATCTCTGTCAAAAAGCATC
AGAGAAGCTTCCCCGCTTAGAATCCAGCATAACTCACTGAACCGAAGATGTGGCAGTTAC
AAGAGAAGTTGAGCTAAGAGAAGTTTGTTTTCTGAAATTAGAAGCAAAATCACACTGCAA
ACGGCATTCAAGCAGGCTTGAAAAGTGTGGTGCATATCTCTGCCAAAAACA
>danio_rerio
TAAAACGATCCAGCCATAAAATGCATCATTCTTTTTTGTTTTAGACAACAATTCATGCAC
TGTTAAACATGTTAAAGCAAGTTGCAAGTGAAAATCTATGTCTCTGACTGAGTTTGCATT
ACTGTGATTTGACCTCTCTGCTGGCTGAGATAAGCTCATTTTCAACGTCCAATTCAGAAA
GTGA
>daphnia_pulex
TTCAAATCCATTGAAGTTGTGTTCACTTCCTGGAAGATCTCTATCAACCCTTATTTCTAT
TTCTTGTCCATCATGGATGTCTTAAGAGATTCATTGATTTTAAAGACTTTAAATAGTGTA
TTGGACATCGAATTAACATATCGTGGAACAAGAAATCTGTTTAAACTTGTAAAAGATTTC
ATATCGTCAT
>dasypus_novemcinctus
AGGAAAGGAGATAGCTGCAAAATCTCTGCACACAATGCTTAGTTCTCAGAAGTAAATCAT
ATGTGCCATGTTGTTTCATTTAGGTTTAAAGAGTTAGGTGCTATACCCTCGGTTATGCTT
TCCTGTATAGGAAATGCTAGTTCTGGAGATACGAATGTTAGCACAAAAACTGA
>dictyocaulus_viviparus
TTGGCATCGAGAAATGTTCGCATAATTCAACATTACTAGATACCAAAATTTCAAATTTTG
CGCTTCGACCACTCTTGATCGTATTACTCATGGATGAATTTAGCAGATTTCTACAGCGGT
TTTCGGCATTTGTCAGTAGGAATATGGAGATCCACCA
>dipodomys_ordii
CCAGCCTCACAGAGCCCTGTCCAAGCCAGCACCACCTGGACCGGCCTGGG
>drosophila_ananassae
ATCGCTAATTTAGCGATAAAAACGGCTAGAAAATCATCACTGTTAGCGCGTAAAACCGCT
AGAAAATC
>drosophila_erecta
ATTTAGTTAATAAATGTGTTCATGTTTGTGTTTGCGCACGAAAAGTGGTTTCATGTGGTG
CGCAGATAAACAATCTACATCCAGAAAGAAGAAAATATAAACTCTAAACTCTAGACCAAG
TCATCGGTAATTGTAATTAAAAACTGGTGCACATAGTGTTCAAAAATAATTCCCAACTTG
TTATGGCTTATATTTCATTATACGTTCCCTCTAACAGCCTATAAAGTAGTGGACAGGAAG
TTCCGTGA
>drosophila_grimshawi
AGAAATTCTAACTTCTGACAATTATTGGCGAAGATACATCCGTTTGAATTTGGAAACAGA
AACTTAAAACGTCAATTTCTCGAAATGACTTCGAGACTACATATTTTCAGCTTTTGATGC
AGAGTCAAAATATATAAAAAGGGCTTCATAATGTTGAA
>drosophila_mojavensis
AATAGTCCTGTTATTGCCTGTTATACATAGAAAAAAGTCAATTTTCTGATCTTTTACCGC
ATTTCAGGGGATAATAATGTAAATATTTCAAAAATGTTGGCCCATTGAAAATCTGAATAC
GTCAATGGATGGGACTCTCAAAGACGCTTCCAACGAAGTAAGTCGTAATGAAATCGGCCC
AGA
>drosophila_persimilis
ATGTATACTGAACCACCGCACCTATATAGCACCCACGCTCCAGGAAATGTGGCAGGATCG
CACATCTTGGGCATAAGCACCAATATGGCACTATAGCCCCAAAAAAGTGGAACTCCGCAA
CTATATGGCACCTGCACTTATATAGAACCCTTCCCTATCCAGCACTGGC
>drosophila_pseudoobscura
AATACGTCTCTCCGCTGGCGGAAAACTTATCTCCGGTGGCGGAAAATTTATATCCCCTGC
CGGAAAACTTATCTCCGCTGACGA
>drosophila_sechellia
TTTGTGCAAAATTTTTGGATTTTTCGATTTTAGATACCAGGCGATGATAATCAGTAGCGG
GTGTCTACTGAAAACCAACTAATCGTTGGTCACCTTCTGGAATTCTTGTTCGCCTGGTAA
TTTAAACCGAAAAATCTCTCAATTTGCAACAAAATGCGTATT
>drosophila_simulans
CCTACAAAATGCGCATTTTTGTGCAAAATTTTTGGATCTTTCGATTTTAGATACCAGGCG
ATGATAATCAGTAGCGGGTGTCTACAGAAAACCACTTATCGTTGGTCACCTTCTGGAATT

CTTGTTCGCCTGGTAGTTTAAACCGAAAAATCTCTCAATTTGG
>drosophila_virilis
ATATCTTGACCAAACTCGGCATTTATTAGTTTTACTATACTCCTCATATATATGCAAAAT
CCTATTAAGATCGGACCACTATATCATATAGCTGCCATAGGAACGATCGGTCGAAAATTA
AGTTTTTGTATGAAAAACATTTTGTTTTTCAAG
>drosophila_willistoni
CAGCCGCCATTTTGAGTCTTCTACCCGCCATTTTGAGTCTTTTAGCCGCCATCTTTAATA
TAGAAGCCATACGAGCTGCTAGAACCATTCTA
>drosophila_yakuba
AGTATATACATGATGCTTGTAAATAATTCCCAACTTGTTCTGGTTATTTTTCCTTATATG
TACTCTCTACCTGCCCATAAACTAGAGAATGTAATGGCCTCTATAAACGTTGATGCAGGC
GATGGGGATTCAGGAGCTGCGCGGGTGTAAAATCTGCATTCAGGACGCCGTGAACCAGGA
CCAAACCAAGCCAGGTTAGTAGTGGAATGAACTATATTTTTTAAAACTATATGTGTTCAT
TGAATAATACACAATTAAAGTCCATCGACATTTAATATTAAAAAAACTGAATTACTGTCA
GCTTACACCAAATATGACATGTCAACTGATTCCCGAAAATATACACAGTCAAAACAAGTT
AAGTTTGGCCACGTAATTGTTAATGATTGTTTGAAA
>dunaliella_salina
CAGTGCACACCCTCCAGGAAATGTGCAGGTGCACCTTGGTGAAGGAAAAGGCTCTAGCAA
CACAACTACTCAGTTCCAAAATGCCACCAGCAGCATTCTGAAGTTCTGAGTACTATAGTA
TTCAGCTACAAAAGACAGCATGAGAAACTGCTGTGTCTCTGGAAAGATATTCACTGCTCT
GTT
>echinops_telfairi
CCTGCAAGCCAGGCAAGGGACTTCCGGGCAGGCTGGGGAAGCGAGGCTTTGCCTGGCGTC
TC
>equus_caballus
TTGCTTCCAGCTCTTTGGGAAGCTAAGAAACAACTCGCTCTGCACAGTGCTCTTACAGCC
TACCGGGAACATCTCTTACAAAGGCCTGTGAAACGCAGTTTCTTTGCAGGCCTGATCTGG
CTGAAGTAGAACTTCTGCTCTGCGTGTGCTACATTGCCCTAGAGCTGAAAGGCACAACGT
GCAGGCTTCTTTCCAAAGGGCGAGATGGGCCCCAAAGCATC
>erinanceus_europaeus
CTCTCTCAGCACCAGGCACAGCACCCAGGGAGCCCATGGAGGGTGGGCAGGGCTGTGGGG
TCTCTC
>eucalyptus_grandis
CGGATTTAATATTTTGGAGATCCCGATACCTTGAAAGTCGGCTGAAATCAAGAAACGGAG
ACTCGGCACAAAAAATTCCCTTGTGTTCCATTAAAAATTCCTTTTCGGTATTTTCACTAT
AGACCCCGGCAGGCGGAAACGGCATGGATAAGTGGGCTCCAAAAGCTTTATTTCGAT
>felis_catus
GCGGGCACTGGGTTCACTGGAGGCTGCAGTGCC
>felis_silvestris
GCACTGGGTTCACTGGAGGCTGCAGTGCCGCGG
>gasterosteus_aculeatus
TGACACGGTACAGTGGTGTAGTGGTTAGCACTCTCGCCTCCCAGACAGATGCCTCGGGTT
CAACTCCACCCAGTGGTGCCTCTGTGTTAGGTGGTGTAGGCTAAGCCTGCATGGGATTCA
CTCCATACTTGTAAAAGTAATGGAACAATTTTTTAAAACTTCGCATCTGAAAAGAATCCC
CAAATATTTTGCAAAAAGTCATGAAATTCTATCTTTAGTCATATGAAGGGGGACGCCGGT
GAGAAAACGTACTTTTGAATAAAAATCTGAATATTTTCTGAATTGGAGTCATAAGACATT
CTAATGGCAGCAGT
>globodera_pallida
ATGCGTGAGACCAATATGAAGAAAGTTATGGCGCTCGGCTTGCATGGATTTGGTAGGGTG
CACCTTAATTTGTTGGGGCACTTTAAATTCATAATATTCCATCATATTATATACCGTTCG
ATTCCTGCCAACAAGCTGAGCACGTATATCTAAACGCTGG
>glossina_morsitans
AAATTTCAGAAAATTGGTTTTTACGGCTCTCAGAACCCACACGTACAGACATGATAAATT
CGCACAATGTGCTGCAAATAGCGTTTTTGCAACCACAGCAAGCTATTAGAATGCTAACTA
TTGCTTTTTATGAAGAAAAGCAA
>glycine_max
TCACTCGGATGTCCGATTCAGGCGCATAATATATCGAGACGCTCGAAATTGAACAACGGA

AGCTCTCGAGAAATTCAAATGATCATAACTTT
>gossypium_raimondii
TTAGGGAGATAAGATCTACAATCTTCAACCTACTCCACTGCTGCTCAGGGAGATAGGACT
GGTGGCTTAAATCTGCTTCCTACTATCTCGGGAAGATAAGATTCGCCGTCTTCGATCTGC
TCCACTACTGC
>haemonchus_contortus
TGGACCTAGAGCGTTCAAATTTGGTAAGAGTACAGGAGACAGGATGGAAACACTCATGGT
TGGTGGCAGGACCGGACTCCGCCCACAAGGGGCGGGGTTTGTGCAGAAAATCATAGCTGC
AGTTCCCG
>heliconius_melpomene
TTGAAACTGGTGTCGATCGTTCGGGCTCCGAGGGGCCTTCAAGGTGGTATACAACCCGCG
GGAATGCGCGGGAAGGTGCGGGCGGTAAGTGGCGAAAACCGATTTTTTCAGTTTTCCCCG
CTCGGATTCAG
>helobdella_robusta
GTCATACTAAACAGCTACAGAAACGACAGCTCAAACTACTGAAACCACTGGTCCAATGAC
GACGCAAGAAACGACTGTCGAGGCTCCAAGTAAAAATTATTTTAATCGAAACGTGTCTGC
CAACAAATTGATTTGATTTTCACACCTGACAACATTGTTTCGTTTTAATCGTATTTCAGC
TACTGCTTCTCCACCTACTGAAGCTCCGACAACTGTTACAACCAAGGAAATCGAAACATC
AAGTCCAGTGTCAACGACAGAAACAGAGACAACAGTTGTTCCTCCAAGTCAGCTTAATTA
GAACTATTAAAATAAATCTTAATTGATTAAATATAGCTTCAACTCAGTAAAACATATTTA
AATTTATTTGACC
>heterorhabditis_bacteriophora
CCTATGTCGGTAATGGATGCACTGTAGGTGCTGCAGTGAGACTCCCTATGCGAGATAAGA
TCATATCGATAGAGAATTTGATGACGATCATTCTGACATATCTAGAATCTCTGTACGATG
AGTAGATCAAAAGTTATGCCCGAAAAACACGTTTTAACGCATTTTGCA
>homo_sapiens
AATATCTTCACATAAAAACTAGACAGAAGCATTCTGAGAAACTTCTTTGTGATGTGTGCA
TTCAACTCACAGAGTTGAACCTTTCTTTTGATTGAGCAGTTTTGAAACACTCTTTTTGTA
GAATCTGCATGTGGATATTTGGAGCGCTTTGAGGCCTATGGTGGAAAAGGA
>hydra_magnipapillata
CCTGAGTGAATCAAAGGAACCTAAGTTTTTTACGACAAAACACTTTTTCTATATAATAAC
TTATCTAAAATTAAAAAATAAGACAATTGCGTCAAAAAAACAGGTTTCCACTGCTTTCTT
TTGGAATCTTTTGATATCTTTTTTATGATTATGTTGGTTTGTTAAAGAATAAGGTCAAAT
AAA
>hylobates_concolor
ACAGAAGCATTCTGAGAAACTTCTTTGTGATGTGTGCATTCATCTCACAGAGTTGAACCT
TTCTTTTGATTGAGCAGTTTATAAACACTCTTTTTGTAGGATCTGCAAGTGGATAATTGG
AGCACTTTAGGGCCTATAGTGGAAAAGGAAATATCTTCACATAAAAACTAG
>ixodes_scapularis
ACGGTGAACGAATAAAACGGCGCCGAACGTCTCTCGTCAGGCCGTGCTCGCTCCAATAAT
TCGCTTTCACCGAGGCAATAGCCGAGATCGCGGTC
>labeotropheus_fuelleborni
CATAATGAAAACCTATACTTTGTTTCAGGCGAGTTTCCCATTCAAATGCATGTAACAGTG
AGAAACGCATTGTCTTGGCGAAATAAAGCGTTTTTGAACAACTTCATATAAATCGCTGTA
ACTTTTGATAGAAGACTCAGAAACACATGTTTATGGCTTTATCTTATAGAACTCAATATC
CCGTGCTGGGCAAACAGGTTTTGCAGCCGTTTGAGCTAAGATTTTAAAATTATTCA
>lama_glama
CGTTCATCCAGCACAAAGGAAAGGGTTGCAGGAGAAACACTGACTCTGGTTTCTCAGACT
GTTTCCTAGTGCCGGTTTGAAGTGCCTGCCGTTTTCACTGTAAAACCGAGTTGGAGCTTG
TACCTCCCCATATATATGAATGGAGTGGAGTTTGCAACTGAAAAGAAACTTTGTGTTCCC
TTCAAGCTATGTGAAGGACGGCTTTCACACACACTTATCTCTCAGAACTGAGCATGTGGA
GAGACGTT
>latimeria_chalumnae
CAGCAATTTTCAGCACCCCCTTTTTGGGGGGCATCCGGATCAGAAATTTAAAAAGCGCTG
ACT
>lemus_catta
CTTTCTCTAAGTGAATGTAAGCGTTCCTATATATGACTCTCGCACTTCATCCAAACTAGA

GTTTAGCAGGCCTACAAACCACCACCCCATAGTTG
>lottia_gigantea
GCAGACTGCCGACCCCACCCTCTAACTAAAATGGCCACAACTACTCCAATTTTGACGCTA
GGGGAATAATTTTTGTTGCAAACGAGAGTTTAACATGTTCCCTATCTATTTCAGCCGAAA
GCAGAAATTCGGAATTTTTGAAACGTTCGAGAAAATCGACTTTGAAGTGTAACCCAATTT
TCAAGAGGAAAATCGCGTATTTTTGGTTAGGGAAAAATTGAAACCAAAAGTGCTGTAACA
AGCAGAGTTTTCAAGATAGGAACCTGAAATTCGAAACACAACAACTTCCAATAGGGGTGT
ACCTTCAGTGCAAATTTCAACTTTACAGCTCTGATACCCTTTGAACGACAGCCTGTCAAA
GTTTTGGTCTTCACCCTTAACATACAATGCAAAAAGACGCTATAG
>loxodonta_africana
TCGGAAGGAAGGCCTGGTGACTGTACCCAGAGGTACC
>macaca_mulatta
TCCTTTGGCACCATAGCCCTCAAAGGGATCCCAAATATCACTTCGCCGATTCCACAAGAA
CTGGCTAGCGAAAGGCTCCTTGAAAGAAAGATGTAACTCTGTGAGATGAATTCACAGAAC
ACAAAGAAGTTTCTCAGAAAGCTTCTTTCTCTTTTTATCGGAGGATATT
>macropus_eugenii
TAAAAGCCTTTCCACACTGACTACATTCATAAGGTTTCTCTCCAGTGTGGATTCTCTGAT
GTTCACTAAGACTGGACCTGTCTC
>manis_pentadactyla
GCTTGAAATTAGTATCCCTCACCTTGCATTGGTGGCTCCATCAGAGATTACTGATGTAAA
TTCATTTGCAGCATTTCTGAAAAATTCAAAACATATATGATCAGACTCCAGAGACTTCAG
AGAGTTACACTTTTT
>marcantia_polumorpha
TCACGCATTTTGGAGTTGGGACGCCGAAGTTCACGGATTTCTCAAGTTTCGAGTTGGAGT
TTTCGAGAGTTGGGGTCGAGATCGTTTCTTGCAGAGATTGTTTCTTCGCGTCTGAGTGTC
CACGGAAGCTGAAAATTTGCAGGGGCGTGGACTTGGGTAGCGCGCTCCAGCCTGCAAAAT
T
>melanochromis_auratus
AGTTGTACAAAAATGCTTTATTTCGCCAAGACAGTGCGTTTCTCACTGTTACATGCATTT
GAATGGGAAACTCGCCCGAGACAAAGTATAGGTTTTCATTATGTGAATAATTTTAAAATC
TTAGCTCAAACGGCTGCAAAACCTGTTCGGCCAGCACGGGGATATTGAGTTCTATAAGAT
AAAGCCATGAACATGTCTGTCTGAGTCTACTATCAAAAGTTACAGCGATTTATATGA
>meloidogyne_incognita
GCCTGGGGGAAACCTATTGCCTGAACCCAACCACACTTGGTCAATTGCACTTTGGTCTAC
TTTGCCTGAGGCAATTTTTAATCGGATCGTCACGCACTCTAAACCATGAATGCCAGGTGA
AATTTGATT
>metriaclima_zebra
CACATAATGAAAACCTATACTTTGTCTCGGGCGAGTTCCCCATTCAAATGCATGTAACAG
TGAGAAACGCATTGTCTTGGCGAAAGAAAGCGTTTTTGTACAACTTCATATAAATAGCTG
TAACTTTTGATAGAAGACTCAGATACACATGTTTATGGCTTTATCTTATAAAACTCAAGA
TCTCCGTGCTGGGAAAACAGGTTTTGCAGCCGTTTGAGCTAAGATTTTAAAATTATT
>microcebus_murinus
CGGGCAGGCAGGGCGCAGTGCGGATCTGGCTGTGTCCACTCACCCACGGCAGA
>micromonas_pusilla
GGTGGCCGCCACCCGCGGCATCGTCGCAAGTCCACTCGTCCCACGGAGCGTCGTTCGCGC
GCGCCCACTGCAGCACCTCCA
>micromonas_sp
CACGGGAACCACAGCGGCCGTTTCAATCTGCGCCCCGTCGACGATGACGATCACCGGCTC
GACTCGACTCTGCTCGACCCGTGCGAGGTGTCGATGCGTCGGGCGTGCCGTCGCGGTCGC
GATGAGCGTACGAAGTGCGATTTCCAACGGTTGAATCGACACCAAGCTAGCACGGACCAC
ATGAACCCTCAACATAACCCTC
>monodelphis_domestica
AGCTTACTGTACACCAGAGAATTCATACTGGAGAGAAACCTTATGAATGTAATGAATGTG
GGAAGGCCTTTCACCTGAGGTCAC
>myotis_lucifugus
CAGCTTTCTACATCTAATCTCGGTGATTGAACAAGGCCATAAAGATAGCACGGCTCTATT
TACATCTATTTCAAGTGTTTCTTACACGTTTCTAATCATTTCCCTGACTTATTGCGTATT

TTAAGAGTTTTACCAAAATTAGACAGTTTTCTTAGAAGTATATACATGAATCTTCAGGAT
TTAAACAGTTTCATTCAAAAAAAGCCAGCCAAGTCAGCCTCACTTGTAAATATAAACAGT
ATTCTCCTATTTAAAGGCTTTATGCACAAATTCTGAGCTCTTTAAACTGCAATTCTTAAT
GTTTGAAAGCACATTCTTCGGAAAAAGACCAAGTAAAGTAGCCAGAATTATAAACATTAT
CAGTATTCTCATCACTTCA
>nasonia_giraulti
CGCTTTGGTTTTAGATTTTATACTCGCTTCGCTCGCCTTCCTTCGATGTGCCGAGGTGTT
TTTTAAATAAATTATTGAGCTCGGGGAGCGTGGTGTAGTTGGGTTTAAG
>nasonia_longicornis
TGGGTTTAAGCGCTTTGGTTTTAGATTTTATACTCGCTTCGCTCGCCTTCCTTCGATGTG
CCGAGGTGTTGATTAAATAAATTATTGAGCTCGGGGAGCGTGGTGTAGT
>nasonia_vitripennis
CACATCGGAGGAAGGCGAGCAAAGCGAGTATAAATCAAAAACCAAAGCGCTTAAACCCAA
CTACACCACGCTCCCCGAGCTCAATAATTTATTTAAACAACACCTCGG
>nematostella_vectensis
GGGCTGTAGGCATTTCCTTTTTGTTATCGAAGTACTCACAGGACTGTTATATAATAGATA
GATCTTAAACAGTTCTATCGTCTACGGCCATACCACTTAGAAAGCACCGGTTCTCGTCCG
ATCACCGAAGTTAAGCTCAGTAGGGCGCGGTAAGTACTTGGATGGGTGACCGCCTGGGAA
TACCGT
>nomascus_leucogenys
ATCTGCAAGTGGACATTTGGAGCGCTTTGAGGCCTATGGTGAAAAAGGAAATATCTTCAA
ATAAAAACTACACAGAAGCATTCTGTGAAACTTCTTTCTAATCTGTGCATTCATCTAACA
GAGTTGAACCTTTCTTTTGACTGAGCAGTTTTGAAACACTCTTTTTGTAGA
>ochotona_princeps
AGTGTGAAAACTGAGTTTCCTTGAGAGCTGCTTGGAAGCCTGATGGTGTGTGAAGTTAGT
GAGCCAAATGGAGTTGTTCTCCCACAAGAACCACTCGACTGATTCTTCTTCAATATGCCC
TTCACCAACATGCAGTGTTTCCTTGACAGAAATAGCTCTCACACCAAAATTTGCGTCTAG
AGCCTTTGCATTGAGGGCTATCTGAAAACGGCTTTGGCGCTGAAGTTGTATGTAAATGCG
GAGTCAGAGGCATGTGAAACCCGCATGTGGAGCATTGGTGTGTGTGCGATGCTCACCAAG
CTTCATTCCAGTACAAGGCACTCAAAGGGCATTAAGCATAG
>oesophagostomum_dentatum
TTTTACTCGAAAAACGCAGTTAAATGCATGCATACACTTGTAAAAACTCATG
>onchocerca_volvulus
AATTTTTCCTTGCACCGTCGGCCTTAAACTCTTTATCATCATCATCACTACTAGAAGTGC
TGTCAGATGAACTTTCAGATACTCTGCTTGCAGAAACCGCATTTTTTAAATTAGCATTTG
GCGTAGCTGGAACCGTTTTTTCGGG
>oreochromis_niloticus
AATGTGTTTCAGGCGAGAAACGCACTGTCTCGCCGAAATAAGGCGATTTTCACCAAGTCC
ATAAAGACAGCTGTAACTTTTGATAGGAGACTCGGACACACATATTTCAGGCTTGGCCTT
ATAGAATTCAGCATTTCCATGCTGGGGAAATAGGTTTTGCCACTGTTTGAGCTAAGATTT
TCAGTTATTGACTAAATGAAAACCAT
>ornithorhynchus_anatinus
CAGTTTGGGCCCCCCAGCCTTTGCCTTCCAGGTTTGGCCCCGAACCCTTTTCATTC
>oryctolagus_cuniculus
GAATATCCAGAAAACACAAACACACACAGAGGCTGTAGCAAGATCTGGGATTGAGATAGC
TACAAATCACCAGATAGTTTTGTTTTCATCCAGGAGTATCTGGACAAATTGTTGTGGATT
TCAGGTGAAATTTAACCAAAAATCACCGTGAAAGTGGTTTGGAGAAAAGCACAATCTTCC
TGTTCGGCAAATGTATCCAATGTCATGTTCACAGAAAGAAATATCATGTTTTCTCAGGTT
TCTGAAGCTCTCTTCCTAGAAAACTCGAATGTGTGGAGTAGTTGACTCCAGGTGGAAACA
ATTAGCGTTTGCCAGCAGAAAACAAAAAGGGTCCTGCTAAAATGCATTCAAAACCTTAAA
GTCCATAGA
>oryzias_latipes
AACTGCAAATGAGAACTTTAACTTTTGGGTGCATTTTTTGCTAAAAAATCATTTTGTCAG
TCAAAAGTGCCAAAAGTGTCAAAAAAGCGTTTTGGCTCTCAGTATGACTGTTTTGAATTT
TCAACTTACAATGTGACAAAAAATAACACTTTTTTG
>ostreococcus_lucimarinus
CTGCGAACGCAGCGCGTCGCGCTCAGACTCGAGATCGGACACGGCAGCGGACTGCGACGC

GTCAACCTCGGGCACCTGAGAAAGCACGCGCTCGAGCTCCGACTGCAGCGCGTCGCGCTC
AGACTCAAGTGCCGCCTTGGACTCGTCGAGCTCAGAACGCACGCGCTCGATGTCCTCCTC
GCGCTCGCGAAGCTGGGACTCAAGATCCGCGCGCACAGACGCCAACTCAGAGTCGCGAGC
GGACGAAAGGTCGGCGAG
>ostreococcus_sp
ACGAACGACGCCGGCGACGACGCGTCTGGCGCCGACACGCAGTGCGATCCCATCATGTGC
GCGCGAACAAGCACGTGCAGAGCCACGCGTGCGTGGCGTGTCCTGCCGGGACG
>otolemus_garnettii
CAGGCACCCCTGAGGGCAATTAGGAATCCAATTAGAAACACCTGTGGCCAATTAAGAGAA
AG
>pan_paniscus
CAAGTGGACATTTGGAGCGCTTTGAGGCCTACTTTGAAAAAGGAAATATCTTCACATAAA
AACTACACAGAAGCATTCTCAGAAACTTCTTTGTGATGTGTGCTTTCAACTCACAGAGTT
GAACCTTTCTTTTCATAGAGCAGTTTTGAAACACTCTTTTTGTAGAATCTG
>pan_troglodytes
CTAGACAGAAGCATTCTCAGAAACTTCTTTGTGATGTGTGCATTCAACTCACAGAGTTGA
ACCTTTCTTTTGATAGAGCAGTTTTGAAACACTCTTTTTGTAGAATCTGCAAGTGGATAT
TTGGAGCCCTTTGAGGCCTATGGTGGAAAAGGAAATATCTTCACATAAAAA
>pan_troglodytes_schweinfurtii
CCAACATAGGCCTCAAAGCGCTCCAAATATCCACTTGCAGATTCTACAAAAAGAGTGTTT
CAAAACTGCTCTATCAAAAGAAAGGTTCAACTCTGTGAGTTGAATGCACACATCACAAAG
AAGTTTCTGAGAATGCTTCTGTCTAGTTTTTATGTGAAGATATTTCCTTTT
>panicum_capillare
GTTTTGGGTCCATGGAGTGGATCGGGTGCGTTCCTTGCGAAAATTCCGACGCAACTTCGT
TTAGCGAACTTGTGCGTTAATGGCACCAGTTGGCCCGTTTTGCACCGACTTTCGTGCAGT
AACGAAACGGTCCGAAACGCCCAAAACATGA
>panicum_virgatum
GCACGAACTTTTGCATTAATTGCACCAGTTCACCCCGTTTTGCACCGAGTTTCATGCAGT
TACGAAATGATCCCAAATGCACCCAAACACTATGAAACGTACCAAAACATGAGTTTAGGG
TCCAATGGGGTGGATTGGGTGCGTTCGTTGCGAAAAATCCCGACGTGACTTCGTG
>papilio_dardanus
TTTCTAGATTCAAAATATGAACCCCCTTTTTGACCCCCCAAGGTTGAAATTTGTAAAATC
CTTAAATGGCAAAGTTATTTATTCATGCTGAAGAGCTCTCATTCCAATTTTCATGCAATT
CTGACCTAAGGTGTGAAAAATA
>papio_hamadryas
GAGATGAATTCACAGAACACAAAGCAGTTTCTCAGAAAGCTTCTTTCCAGTTTTCATCTG
AGGATATTTCCTTTTTCACCATAGCCCTCAATGGGCTTCCAAATATCACTTTTCAAATTC
CACAAGAAATAGGCTAGCGAAAGGATCCATGAGAAGAAAGATGTAACTCTGT
>pediculus_humanus
ATTAGCGCTATAAACGGCTAATTGGTCTGCTTAAATGTTTAAATTATCCGGTTAATCGGA
TAAATTAGAATCCGATAACGGATTAATTTGTCATTTATGCGCCCAG
>peromyscus_maniculatus
TTCAACTCCGTTTAAGAAGTTGAATTGAACCCTAGTTGGGCCTTACAAGAAAAACACTCT
GCTTTTGAGAAACAGGCACTCTTGCACTCTACTGTGTTTCCTATAGGGCCAGTACAGTGA
GCTAGCTCAGAACAAAAGAACTGTGCTTCCTAAACAAACGGGGCATATCTAGTTCAACTC
AGTATCAGAAACTAAGTAAAACAAGAGCTTCTCTACAGTCCAATACACTGCACTCGCTAG
GAACAGACAAGAATGCTATTTGCACTGTGTATGGACAGAATAGCACAAGAGTCGCCTTCT
TACCCTGAATGAACACTTAATGCTAGAACTTAAAACTGAATGTGA
>petromyzon_marinus
GTGAATAGCAAAAAGCATTCAACCCCATAATACATGTTTTTAATTGGTCGAGCGTCCATT
TCATATCCCATAGCTTAACCACTCATATCTTTGCATTCCGCTCGATGAGGCGAGCAGTAC
GAGTACCACCTTGATGGGTCTCCGACGTTTCCTTCCAGAGTTATTACAAAAAACCACAAA
CATCCCAGAATCCTCTCGATTTGGGGTCAAAATGTTGTCGCTCAATAAACCCCATAAGAA
ACGTCGCATCGAATAAATGTGATTGAATTCTCCCCGTCTGGACGAGCGGGATTGAATGGT
GCAACTGATTTTGCTGTGCTGTGTTAAATAAGATATTTGCATGACTGCTTTTGTTTTATT
TT
>phlebotomus_papatasi

GCTCTAAATGGATGTAAAGTTTGAGGCCTCTATCTCTCATAGTTTCCGAGATAATCAACT
TTAAAGATTTTCAGACACTTTTATGCATTTCTCGTCCAATTTTCTTAATTTTCCAGTGAA
ATTTTGCACATATCAAGCCTGAAAGAG
>physcomitrella_patens
AGATGTAAGGTTGCCCAATTCATTTGGCAATGATGTCAAGCTTGAGCACCATTCTATATC
AAAGGTAGTCAA
>polychrus_marmoratus
AATACGTAGGAAAGAGAATGACCCTTTCTAAATGTATTAGGAAGGATATTGTACCTGTCT
CCCTTTAATATGTAGGAAGGAGAAGGTACCATTCTCATTT
>pongo_abelii
CTCCAAATATCCACTTGCAGATTCTACAAAAAGAGTGTTTCAAAACTGCTCAATCAAAAG
AAAGGTTCAACTCTGTGAGATGAATGCACACATCACAAAGAAGTTTCTCAGAATGCTTCT
GTCTAATTTTTATGTGAAGATATTTCCTTTTCCACCATAGGCCTCAAAGCG
>pongo_pygmaeus
TATGTGAAGATATTTCCTTTTTCACCATAGGCCTCAAAGCGCTCCAAATATCCCTTTGCA
GATTCTACAAAAAGACTGTTTCCAAACTGCTCAATCAAAAGAAAGGTTCAACTCTGTGAG
ATGAATGCACACATCACAAAGAAGTTTCTCAGAATGCTTCTGTCTAGTTTT
>populus_balsamifera
GGTTGGCCGACCAGACCTCCTCGTCACCGAGTAGCGATTCCATAGCTACCCCAAGGAAAA
CGGCATCCGGAAACTCGTGTAAAAATTTGAGCGCGATCCAACGGTCGGATCAAAAGTTAT
GGCCCTTTTGACCCGTTACTCAA
>porites_lobata
GCCTCTAATTCATCGTTTTCAGCTAGTCTTATGGTGCTTATCATCACAGCGACATTTTGG
AGCTCTTACAGTGAAATTCTCACTTAACCCATTACGACCAAAAGATGGTCAATTTGCAAA
ATACTGTCACAC
>pristionchus_pacificus
AATCGAGTTATGATTTATGGAAATGATAGAAGCACCACTTGTTCACGTAGAACAGGCCTG
ACACCTCTTTTGTATCAAAAGTGATACAAAAGATGAGTTCGCATGGAAGAAGGCCTTTGG
CTATGTGACCACCTAGCGCAAATGTCTTCTAA
>procavia_capensis
ACACAGAGAATTCCAAGTATAACTACAAGAAAATAAGAGATACTAAGAAAACTGGGAAAA
CCTACATTGCTGGTAGAGTGGGGAGTACTTAGCATTGTGAAAACAGTACTTTCGCAGAAA
GAAATCTACTGCTCTAAGAAAGCACAACAGTGAACCGAGAATGTATGAGTTTCTTAAGAT
TGGTAACAGATAGAAACAAGAGAAAGTACACAGTGATTCTTCTACCCAGAGAAGTTACAG
AGGTTT
>prunus_persica
GACCCCTCCCACAATATTATGATGTTCCCCAAGTTTTGGACTCAATCCGATATCGATTGG
TCCATGAAATCGGACAATTCAGGTTCGTACGAAGTTCGTTCTGAAATGGAGTGGTTGGTG
TATTGTATAGTTCTAGACATTGGATTTCACTCAAAACCCACCAAAT
>pteropus_vampyrus
CTATGAATGTAATGAATGTGGGAAAACTTTTTGCTAAAATTCACACCTTAGTGAACATCA
GAGAACTCACACAGGGGAGAAACC
>rattus_norvegicus
AAATTTTAACGAACGGAATCTAAGTATCTTGGTGAATTCAGTTAGTTCCCAATAGGACGC
GCTTGTAATAAGTGTACTTTTCAAGATATAGCA
>rhamphochromis_esox
CTGTAACTTTTGATAGAAGACTCAGACAAACATGTTTATGGCTTTATCTTATAGAACTCA
ATGTCCCCGTGCTGGGAAAACAGGATTTGCAGCTGTTTGAGCTAAGATTTTCAAGTTATA
CACATAATGAAAACCTATACTTTGTTTCGGTCGAGTTCCCCATTCAAATGCATGTAACAG
TGAGAAACGCACTGTCTTGGCGAAAGAAAGCGTTTTTGTACAACTTCATAAAAAATCG
>rhodnius_prolixus
TTTCATGACCCAAAAAATTTTCAACCCTTTATATCTTCTCAATTTATGGATCTGTACACA
AATAAACTATAGCTTATAACTGAGGAAAACAAAGTGTATAAGAGAACAAAATTTCATACG
AATATGTTGTTTACAGAGGAAGATATAGAGTGGCCGTAATTTTACAGGA
>ricinus_communis
TATTTGTGAATATGAGAAAACAACCCCGTAAAAGGGGTA
>saccoglossus_kowalevskii

ATTGCAGAATACACCAGTTCTCGTCCGATCACTGAAGTTAAGCTGCATCGGGCGTGGTTA
ATACTTGCATGGGAGACCGGCTGGGAATACCACCTGCAGTAGCTTATCAACATTTTTATT
CCACCGTACATACCAACTTTTTCCCGGGATTTTTTGCCTAGCCGAAGCGTTAAACGACAT
CAATTCCGACGAATATTCAGCCTACTGCCAAACC
>salmo_salar
GAAATTGCACTATGTTTTGGTGGGGACGTGTCCGTAGCATTTGGGTTACCAGGGGTGAAA
TTTTACAACTGTTTTAAATACTTTTAAAGGCCATAACCACCCCCAGGACACCCCTGACAG
CACCCCACTACGGGCCCAAGTCAATGGGAGCAAGAATGACTGATTTATGGAAGTTTGAAA
AAATCCTCAGAAAATATTCTATGTTTTTCTTCTCAGAAAATCGGCTATGTTTTTCTTCTC
A
>schistosoma_mansoni
CTATGAAAATCGTTGTATCTCCGAAACCACTGGACGGATTTTTATGGTGTTTGTTTTAGA
TTATTTGCGAGAGTGTGGGCGTTAATATAAAACAAGAATCATCTCAAATCCGACACAGCC
GTT
>schmidtea_mediterranea
GACGAGCCCGTTATGATTTTTGCAATTTTTAAAATGCTATCATGTAGAGAATCAAAAGTT
ATCGAGAAATATATCAAAGATTATGAAAACAAATAAATATTGACAGAGATACAAGCCTAT
AAAGATTAAAAAATTTCCGTCCAGAATGACTTGTGTTT
>selaginella_meollendorfii
ACATGCACTGGAAGTCTAGCTGGGGGTGTGGAGCCGGGCACCCGAGGTCTGACCTCGGCT
GCCCAACACTCGCATCCCCCACTATCCTTCCAGTGCTCACGGCCTACCACGCCCCAGGGA
GACAAGCGCTTTGGGCTTTCACCCCCACGTGTCCATGTCCGAGCCGGAACCCGAGTCCGA
GATGGAGCCCGAGTCCGACCCCGACATGGTGCACGCGGGGAGGAGCCCAGCAGCAGCCCG
C
>setaria_italica
AAATGCACCTGTTTTGCATCGTTTTTCGTGCCGGAACCGAATGCTCAAAAACACTCCCAA
ACATGTTCTAGGGTATAATTAGGAAGATTGCATGCGTTCGTTGCGAAAAAATCCACCCGA
GTTTCGCTACCCGAAAATAGTGCATTCGGGTGCCG
>solanum_phureja
AGATCACCAAAAAGTCCATGGACTATAGCACACGAAAATCGGCAAAATTGGGGGTTTACC
TGCTCTGGGGCACATTTGACCTTCAAAATGGGATGTTTTGGCCGTGAGGGCTAACTGGCT
CCATAGCTAAGGTCTTAACGGACGTCCATGAAAAATTTTGGCAAAAATTATGTCGGAATT
CC
>solanum_tuberosum
CGTTAAGACCTTAGCTATGGAGCCAGTTAGCCCTCACGGCCAAAACGTCCCATTTTAAAG
GTCAAATGTGCCCCAAATCAGGAAAACCCCAATTTTGTCGATTTTCGTATGCTATAGTCC
ATGGACTTTTTGGTGATCTGGAATTCCGACAAAATTTTGCCAAAATTTTTCGTGGACGTC
>sorex_araneus
CACGCATGCGCAGTGCGCTCTGGCCGC
>sorghum_bicolor
GAGATAGTGTTAATCTTGATGCAAGATAGGTGCACGGTTTGCACGGAACGCACCATAGGC
TAAGAAACCATTTTGGACGCACCCGATGGAACTCCTAGATGAAGTGTGTCAAATGGAAGC
TCGGTTCGGTCTGTTTG
>spermophilus_tridecemlineatus
CTAGCTCTTTGAAACTTGACACAATAAAGCAGTTTCAAGTTCTATCTCTATGGTTTGTTT
GCTGTTGATGTAGCCCTAAGCAGAGACTGGCGAGAATCACTTTCATGGAAAGGGCCTGCC
AAGCTCCAAAAAACTACGAGTTGCCTGAAATCTCAGCAAACACTTTTTCACCCAATCTTG
ATGATACTTCAGGTAATGATCAGTACACCAATCCCACTTGGCTCGCCAAATTTCGTTCTT
CTAGGTTAAACCGTTCGTTGCAGTAACCGGTTTCATTTTGAGGTTTCACTCTGTTTTCTC
CTATAGGGATACATGTATTTGGAACTCTAAATCAAACACGGTTTGTG
>spirodela_polyrhiza
CACCAATTGAATCTGGAAGATATTGTAATTTTGTACTTGACAAATTAAGATATTCTAACT
TTATAAGTGATCCAATAGTACTTGGAAGACTTTGTAATTTTGTTTATGACACATCTAAAT
GTCTTAATTTCACAAGAG
>strongylocentrotus_purpuratus
TTATTTGAGCGCATATCAACCAATGTGCCAGAACAAACCTTAACGTTTAGCGCATATAAC
GTCCAGAGTATATAGAAGGGGGTTGGGGTTAATACATTCGTTGCTGATTTCCCATTTGCT

TTAAATGTGCTGAAACTCACATGAAATGTAGATTATTATATACCTCACCCAATACGTGGT
GCAAAAATTCTAGCAGCTGACAATAAAAAGGTC
>strongyloides_ratti
AGAAGCCTTTTTAGGGTAAAATATGTGTTGCTGAAACCATAGGAAAATGAAGATTTAGAA
GTCTAACAATGAAACCATTGTAATCTACATGAAAAATTAATCTAGAATCAACTTTTTTAT
TTTAATATATTTAAAGTCATTGATACATAACTGAATCTTTAATATAAGTTAC
>taeniopygia_guttata
ATGTTTGCGCTCCGAGTCCTGTGGCCCTGAGCCGGCAGCTGACTGCAAAAAATCCGAACA
TCGAAGGATAAGTCCCAGACAGGAGCTGCCCCCACCTCATACTTGTGCTGCCCTGACAGT
CCCAGGGGAGCCCTACAAACTGACATCAGGAACCTGGACTGGTGAGTTTTCTTTCCCAGG
AAAAAGAGCCG
>takifugu_rubripes
TGCATTCACCTCTGTTTTGACAAAAATGTGTCTCCTGACCAAAAGTGATGGTTTCCCCAC
GAGAAAACGTCAAAAACGTCATAATGTGACCGCAGCATGAGTTTTCAGATGATCATGT
>tarsius_syrichta
TGACTTAACTAGCTAGCCAGCTGGCTAGCCAGCTGACTTAACTAGCTAGCTACCTAGCTA
GCCAGCTGGCTAGCCAGCTGACTTAACTAGC
>teladorsagia_circumcincta
CACCTTCGGTCCCCCACCAAATATGTTCCCGTCCCACCCCCTACATCACTTGTTGGAACC
AACGCCTCACCGCTGCTGGGACGCGTGCTATGAATTTCGTGAAATTTGCATTCCAGCGCC
TCTTATCGGCTCTTATCAGCCGTTAT
>tetranychus_urticae
CCAGCATTTACTTTACTCAACTATTTGTCACACATGCAAAAATTTGATATTTGATGATTC
ACCTTGATAAGCTTTAAAGAGTGATGTCCAGAAAAATAGTCTCACGCGAAAATAATTTTA
ACCTTTACTTTAACAGTCAAAAGTTTGAAACAAAATCCAAAATTTACACTTTGA
>tetraodon_nigroviridis
AACACTGGAAATCTGTGTGCTGCATTACATTTTGTCAAAAGTGAGCTTTTCTCGCCGAGA
AAACGCTGTTTTTGACAAAATCCATCATTCTGCACCAAACGCAGGTGCCAGAACTGAA
>tribolium_castaneum
TTGTCAAAATTTATCGAAACCAAATTTTCTTCATTATTTTTCAACTATTCGGAGTTATGT
CCTTAAAATTAAGTGTTTCTAACGTAAAATGACCGGCGAAATCCAAATTTGCAAGAATCA
AGTCGCTACGACTAACCGTTCTGAAAATATCGGCAAAAATTTAGCC
>trichinella_spiralis
AATATAGTTAAGCATTGTTAATCATTGCATTTCAGACCGCACCAAGGAATATGAGCGGTT
TATCGGTATGCAAACTCTGGTAAGGAGTTACTCTTACCAATATTCCGGATTTCTACCTGT
GTTTCTCCCCACAGAAGTCAGGTAATGGTTCCTCTCTATGGCCTGTAATACTATCT
>trichoplax_adhaerens
GGATGATTGTCACCAAGTGTTGCTAGTTCAATCTTGAGTGATTTGTTATACATTGATAGA
GCATCATCATACTTGCCTTGATTACAATAGACAAGTCCAATGTTGTTATATGACATAGCA
ACATCA
>trichuris_muris
GGTATCGGCGAAAACGACAGCCATCATCAGTCGACCAGCACGAAAGCAGAGCGCACAGGA
AAGATCGGTTGTGTTTAGGTCTAGGCTTA
>tupaia_belangeri
TTTTGGCTGAAAATCTACATGCTTATCTCACGAAAGTAAGCTTCATTTCAAAACGTCCAA
TGAAATACACTGTAAAACTCCTTCCAAAACTGAAAATTGAAGTAGAGCAACACTGAACAT
CACAAGTTGTTCAAGTCCTCAGTTAAGTAAGTGGAACAACTAGTTGCTGTAGTTTTTTTA
ACATTTCTTGGGAAGAGGGTTTCTTAGTTCTAGTTTGCTTTGAAAGTGTAGTTCATTCTT
AGGCCCAAAACTAAACTTCCAGCTACTACAAAAATCTTTAGGTGTGAAAATAAGCTCCAT
GAGTGGAGTCATGCTGCCAAAGATCTTTCCGACATTCCAAGGAAAAGTACAGA
>tursiops_truncatus
AACCAGGCAGGATTGACCTCACACCAGAGGGCCCACATCACAAAGGGGATGGG
>vitis_vinifera
AGTACCGAAAAAGGGTCGAATCAGTGTGAGTACCGAAAAATGGTAGAATCCGGGCGAGTA
CCGGGAAAAGGTAGAATCCGTGCGAGTATCGAAAAACTGTCCGGGCG
>volvox_carteri
CACCACCCGCGGCGTTCTACTAGTGCAGTAGCCAGGCGCGCTGTGTCCACTGTCCTAGTA

GCCGCGGCGCTGACGTGT
>xenopus_tropicalis
GAGATATGTAACTGTCCCTGGGCTGCAATGGTATCTTCCAGCCTTTGCCCAGGCT
>zea_mays
CTTTAGGTCCAAAACTCATGTTTGGGGTGATTTCGCGCAATTTCGTTGCCGCACGTCACC
CATTCCGAAAACGGGTATCGGGGTGCATACAAAGCACGAGTTTTTGCCACCGGAACAATT
TCTTCGTTTTTCGCAACGAACATGCCCAATCCACTA
>Jatropha_carcus
CCCAAAATCGTAGGTATACCCCTTTGGTCTTAGGAGCCCTGTGTGAAGCCCTGTTCCGGA
GCCGATATCTTCTGTACGCCTGATGGGAAGCTCCTATAGGCTCATCTAGGGGATATTTTA
GGGTCTTATGGTCCAATCGGTATGCCTATCTCGGCTGTTCAATGCTTCATTAAGGATTGC
AAACTCAATATAAATTAATGCGCAAAATCGGTGCCACTCCGGAAGCATTTGGCAGTTTTA
GCCCAATATCTCTCTGTCTATCGCTTGTCAAGCCATTCAAGGCCATAATATTTCCTCTAA
GACATATAGAAAGCATTGTAATCATTTTCGGGCAATTCCGAGCCGGTGCTTCCGGGC
>Mus_musculus_castaneus
GAAAAATGAGAAATACACACTGTAGGACGTGAAATATGGCGAGAAAACTGAAAATCAT
>Bos_taurus_taurus
AATGGAAGATTGGACTTCCTGGGCAACACAAGAAGGCCATCACTGATTTCCCCGTTCGTA
ACTCGAGATTCCGCCGCCAAACTCGAGACACCAACGTGGATTCCCCCGTCATCGCACAAG
ATTGAAGCCCTTTCCGCTACAGCGTCTCAGGAGAAGTCCCACGTTAGGAATTGAGCGGTG
GAACGGTACTTGGCACCCTTGACGGCGGCCCCACAAAGTTCCCCGGCATCCCGTCTCCCT
GGAGAGGAACACCGAGTTTCCGGCACCACTTCCTCTGAGCCCCTTCTACCCTCCTGATCT
CGACAGGGAGGGTCGACTCCCCTGCTTTGTCTGGAAGGGGTTCCCGACCTTCCGGTCGCA
CCTCAGGATGAGGCCGGGCTCACGACGACATTCCAGACGTGGCCTCGTGGGTGCTTCCAC
ATTGCGAAGCACCCCGATTTCCCGGTCCCCTCTTGGTAAGAACCCGATGCCCGGACACCT
CTCGAACTCCACCCTGTGAATGAGGTCAACACGAAGGGGCAGTGCCCGCCCGTGCATCGT
CCGGAAAGAACCCCCAGGTTCCAAATACAGCTCGACAAGTGGCCTCTCTCCCCGGGGACA
CCTCGAGAGGCAAGCGGAGTTCCATGCCTCAACCCAAGACGAGGACCTGACTCTCCTGTC
CCCAGTCTGCAGGGACCCTGCGATTCGGAGTCTGAAATCAGAGGAACCCTGAGGTTCCTG
CCTCAACTGGAGATGGAGGCCCTCTTCCAATGCACCAAAACCCCGTGGAGTCCCGAGAGG
CCCCTCCCACCTCCAGTTCCGCTGATTCTCAGAGCCCACCATGAGAAGCCCCCTGAGGTC
ACCTGCACAAGTCGAGGGAAGCCAAGGGTTCCCTGCCTCAACCCGAGAAAGACCTCGAGA
GACCTTCTTCAACATCGTCTCGAGGCCAGGTTCCCTACCAGGACTCGAGGGCAATGACGC
GCTCCCCCTCGCCACTCGCGTGGAGACCCGACTTCCCTGGCGCCCCACGAGAGGCTCACT
GACCTCGCCGTCGCACCTCGTGAGAAACCGCACCCTGGGGCCGCCGGCTCGAGAACCACC
CGAGACTCCCCCGTCATCGCGGAGATGAGGGCCTTCGTCTCCTGCATGGGCCTAGAGCCA
ACCTCGCGACCTCTCTCCAAACGCCTCAGGAGGCTGACTCCCATTTGTCCACCCAGTGGA
GCTCAAGAGATACCCGTCGCGACTCGGAGGCAGAAAGTTGGGTTCGTTGCTTCCCCTCGA
GATGAATGCCTGTCTCCCCGGGTGCGTCTTGGAATGCCACCCCGAGTCCCGGTCGCCCCT
GGAGAGGAACCTCGGGCTTCTGGGCACAAGCCTAGATGAAGGTCTATCAGGGCCTGCAAG
TCACTCTGGAGCAATCCCCAGCTTTTCTTTCGCAACTCG
>Mus_musculus
AAAACTGAAAATCATGGAAAATGAGAAATATACACTTTAGGACGTGAAATATGGCGAG
>Mus_spretus
GTGTATATCAATGAGTTACAATGAGAAACCTGGAAAATGATAAAAACGACACTGTAGAAC
ATGTTAGATCAATGAGTTACACTGAAAAACAAATTCGTTGGAAACGGGATTTGTAGAACA
>Oryza_sativa
TGCCAATATTGGCATTAATTGACAAAAGTTCGCCGCGCGAATCACGAAGTGAGTTTTTGC
CACGAACGCACCCAATACACTCCAATATGTCCAAAAATCATGTTTTGGCCTTTTTGAACT
TTTTCATTCCGGTAAAAAACATCGCACCCACGTG
>Sus_scrofa
CGCTGCATGGAATCCACTGCATTCAATCACGCTGCATGGAATCCACTGCATTCAATCGGG
ATGCATTTTACCATGCTGCATGGAATCCACTGCATTCAATCACAATGCATGGAATCCACT
GCATTCAATCAGGATGCATTTAAGCCACTGCATTCAATCGCAATGCATTCACCACGCTGC
ATGGAATACACTGCATTCAATCACGCTGCATGGAATCCACTGCATTCAATCGGGATCCAT
TGCGCCA
>Canis_lupus_familiaris

GATCGGAAGAGCGTCGAGAATGATACGGCATACGAGATCGGTCTCGGCATTCCTGCTGAA
CCGCTCTTCCGATCTAATGATACGGCATACGAGATCGGTCTCGGCATTCCTGCTGAACCG
CTCTTCCGATCTTCAATGATACGGCATACGAGATCGGTCTCGGCATTCCTGCTGAACCGC
TCTTCCGATCAGATCGGAAGAGCGTCGTGTAGGGAAAGAGTGTAGATCTCGGTGGTCGCC
GTATCATTA
>Sacrophilus_harrishii
CTTCAATAGAAATCAAACCTTTTCCACGCATTTCTGTCCGCATTGGGTTCTAGATTATTT
TTACGATCTCCAATCCAGAGAACATTTGTTCTAGATTTTTTAGTCTCATTCATTTCCGG
AATCTTTTCTGGAA
>Gorilla_gorilla_graueri
AACCTTTCTTTTGATACAGCAGTTTGAAACACTCTTTTTGTAGAATCTGCAAGTGGATAT
TTGGATACTCTGAAGATTTCGTTGGAAACGGGAATATCTTCATATAAAATCTAGACAGAA
GCATTCTCAGAAACTTCTTTGTGATGTGTGTCCTCAACTAACAGAGTTG
>Mus_famulus
CACACTGTAGGACCTGGAATATGGCAAGAAAACCTGAAAATCGTGGAAAATGAGAAAAT
>Gorilla_gorilla_gorilla
GAAAGGTTAAACTCTGTGAGTGGAACACACACAACACAAAGAAGTTACTGAGAATGATTC
TCTCTAGTCATTAGACGAAGATAATCCCGTTTCCAACGAAAGCCCCAAAGAGCTCCAAAT
ATCCACTTGCAAACTCCACAAAAAGAGGGTTTCAAAACTGCTCTGTCAAAA
>Echinococcus_multilocularis
ACCAAAAAAACCACCAAATCTACTCGAACTACTCCCAAAGTGACTAAGCCTACCGTCACC
>Mus_caroli
TATGAGTGAGTTGCACTGAAAAACTTAGAATATAAGAAACGCACAGTGTAGTACATAGTA
>Hymenolepis_microstoma
ATCCCACACAACCACCTCAACCAAACACCACTTTTAAACCTTATTACACCCCAATACACC
TATTCTTGCCATTTGCACCACCAATTTCTCTTCCATTTAGCCAAAATCTTCGATTCTTCC
AAGTCAACCAATTTTTTCCACCTTCGCCAATTCCATCCCCATTTTCTCCCAC
>Nippostrongylus_brasiliensis
ATCACTTTGGCACTGTTCGCTGAGAAACAAAAATTTTCACTTGAACCGGTGTGTCTGTAG
ATGATCTTCATGGGTCTGCCCATGGAGAACATCTTCTGTAGGAGACTGTCCACGAAGTTC
AGCTCGTTGAGCAC
>Bos_taurus_inducus
AATGGAAGATTGAACTTGCCCTGGGCCAACCAAAAGGCATCCTGACTTCCCCGTCGTAAC
TCAGAATCCCCGCCCGTAACTCGAGAAAAACCACGTGGCTCCCCGTCCATCCGCAAGATG
AAGGCCCTTCCCGCAACAGCCGGCCCTCCAGGAGAAGTCCCACGTAGGATTGGAGGTCGA
AAGGGCACTTGGCGCCCTTGATGCGACCCACAAAGTTCCCCGAAATCCCCGGTCTCCCTC
GAGAGGAACACTGAGGCTTTCGCACCCCCTCCTCTGACCCTTTTCTCCCCCTCCTGAATC
TGGACAGGAGGTCGACTCCCCTGCTTGTCTGGAAGGGGTTCCGACCTTCCGTCCACCTCC
AGGAATGAGGCCGGTCTCAACGAAGACCATTCCAACGTGCCCCTCCGTGGGTGGTTCCCA
CATTCGTAGGACCCCGATTCCCGTCCCCTCCTGGATAAGAACCCGATGCCGGACCACCTC
TCCGAACTCCAACCCTGGAATGAAGTCAACACGAAGGGCCAATTTTTCCGTGCATCGTTC
AGAAAAACCCAGTTCCAAATACAGCTCGACAAGCCGGCTCTTCTTCCCCCGGGACCATCT
CGAGATGCAAGCGGAGTTCCATGCCTCAAACCAAAGACAGCCGACTCTCCTGTCCCAGTC
CTGCAGACCTGCGATCGGAAGTCTGAATCAAGGTACCCTGCGGTTCCGCCCCTCAACTGG
AGATGAGGCCTCTTCCAATGCACCAAAACCCAGTGGGGTCCCGAGAGGCCCTTCCCACCT
CCAGGTTCCCTGCTTCTCAGAGCCACCATGGAGAAGCCCCTGAGGTCACCTGCAACAAGT
CGAGGAACCCAGGGTTTCCTGCCTCAACCGAAAAGACCTCGAGAGACCTTCTCAACACGT
CTCTGAGGCCAATCCCTAACATGGCTCGGGAATCCAGTGACGCGCTCCCCCCCACCACTT
CGCACTGGAGAACCCGACTTCCCTGGCACCCCACCAGAGGCCTCACTGACCTCGCCGCGT
ACCTCCAGTGAAGAAAACACCACCGGGGCCGCCGCTCGAAGAACAAACCCCCGAGAATTC
CACCCTCATCGAGAGATGAGGGCCCTCCGCCCCCTCAGGCCTAGAAGGCCCAATCCTTCC
GCGAACCTCTCCAAAAACGCCTCAGAGCCTGACTCCCTTCGCGAGTCCCACCCAGTGGAG
CCCAAGAGATGAACCGTCGCCTGATTCGAGAGCCAGAGCGGCCCTCTTTGCTTCCACTTC
CGAGGTGAAATGCCTTGTCTCCCCGGTGGTCTGAATGCACCCCGAGATCCTGTCGCCCTG
AGAGAAAACAATTGCTTCTGCACAAAGCCTAGAATGAGTCCTATTGGCCCTCCAGTACAC
TCGATGAGCAATCCCCCAGCTTTCCCTTCGCAACTCCA
>Danio_albolineatus

TTTAAGCAAATTCTGTCACTTGCTCTTTTTGCACTTACCAAGCTCTGAAAAGCAAGTTGC
AGAATTCTAGGAATTGACAATTATGAAACATTATCCTTTTGCTTAATTTTCACTTGCAAG
TTGTTAAAACATGCTTAATACAGCATGCACTGTTGTTTTTAAACTTAAAGAATGATGATT
TTCACTGT
>Danio_nigrofasciatus
GTTTAGAGGGCCCAGAACAGAGAGTGATCAAAACAAAAAGAAATGATGAAAACCATCATT
CTTTTTGTTTTGATCACTAACAGTACAGGCAACTCTAAACACGCTTTTCAGAGCAAGAGA
AAGCTGAAAATAAACAGTATGGCAAACTCAGCCAGATCTGCTGATTGTCACTTGCAACTT
ATTAAAACAC
>Echinococcus_granulosus
CACCACTCAAAAATAACAAAAACAACCAACAACTAAAAATAAGTAAACCTAAAACAATCC
AAACTAATCATAGCACAAAAAAACCCACATTCATAAGAACTA
>Caenorhabditis_elegans
AAACTGAGAATTATGGAGAATATGGAAATTCAAATGTGCACTGGTTTTCAAAATTTTTT
>Ailuropoda_melanoleuca
TGAGTAAGGGAGCATGAATGAAAAAGTTCAAGATTTTCGAGGACTCAGCGCCCTTCTCAT
TGGTAATAAAGCAGAGCGTCCAGGAAAACACCTCCAGCGAACAGCAGCCTTTTCACTGGG
GACCCCGATTCCACATTGGGTGTAGGGGCCTGGGGAGGATCCAGCCCTTAGAACCAAGAA
ACAAAGTGTGAGGGGAAAGCCCTTGAAGTCACGACGGCCTTATGGAAGGTGGAACAGGAA
TCAAGGTGCTTTGGTACCGTGAGTGAAACACGACGAACACAGCACTGAGAAAGACAATTC
GGCCAAAACCCGCTTCCAACGAAATGTTTGCTCCCTGGGGAAATCCTGTCAAAACTGCTG
GCGGCAATGGAACGAAAAGGCCCAAAACACGTCCGTCAGAAACAACACCGTACAGGGACC
GTCTTAGCACTATCATCACCCTTCGTGAATAGGGAAACATGAGGGAAAGTGGTCGCGGAA
GCCTGACGAGAGTGCCCCATAGTGGTCTCTGAGACACCACACAGTCCAGGGAAATATCAA
CGATCATCATCTCTCG
>Bombyx_mori
AGTGACCCCCGATTCCCTACA
>Glycine_soja
TTTCTCGAGAGCTTCCGTTGTTCAATTTCGAGCGTCTCGATATATTATGCGCCTGAATCG
GACATCCGAGTGAAAAGTTATGACCATTTGAA
>Drosophila_melanogaster
TATTC
>Drosophila_albomicans
AAAATTTTGGAAAATAAAAAAATTAAAAAATTGGG
>Bombyx_mandarina
CCACAGTGTACGTCCACCACATAAGTA
>Bubalus_bubalis
CCCAGCAAGCGCCTGCCAGACTGGGCTTCCCTGGAGCTCTCCCCCAGCGTAGAGTCGTGT
GGATGTGGTTTGGGGCCTTCCCCGGTGCGTTTGGGCTAGGGAGAGCACAGCCCCAGCGCA
GGGGAACCAGTCCCTGAGCAGGGAGCTCCGGGCAAGTGCTCTGTGCCCAGGCCTGGAAGC
CCGGTTCCCAGCACGCCGGAGAGAAAGAGAGGGGTTGCGGGCCTGTTACTGGGGAGGACC
GAGCGAGGCTGCCAGAGCGGCGGAGCTCCAGCTCTGCGCGCCCAGGCGCGATAGCGCAGA
GCCCCGGCCGAGAGGCTGCTCTGGCCTGGGCGAGCCTCGCCCAGCGCTCCCCAGCTTCTA
GCGTTCTCGCACCGGGAAGGAGCAGGCTGCGGGAGTGGGGGAGACGTGCCTGGCGCCTGG
CTGGCCTCAGCTCCGCTCTGGCCTTCCCTTTGGACCTAGGAGCCAGGCCTCTTTGCAGAA
GGCTCAGGGGCCGGGGGTGTGCAAACTGCCAAAGCCTCCCGCAAGCAGAGCTCTTTGTCA
GGCAAAGAGAGCGCCCAGGGCTGCATTCCCAAGCGGTTGCTTGGCCGCCCAGACCTAGCG
CTGGGCGGAGGGAATCAGGGTCCAGGGCCCCTGGCTGGGGGAGGCAGCACCACAGCCAGA
GGGAAACTCCGTTTGTG
>Onchocerca_flexuosa
AGGGGAGAAGAATACGAGGAGCAAGAAAAAAAAGCAAAATACAAAACGAGGAGAACACAA
GGGGCGAAAATCAGAGGAGGATCAAAAGTATGCCACTACGTGGAGTAAGAGAAGTGTGAA
AAACCCGAGGCGAAGAAAC
>Ficus_fistulosa
AAAACAAGCTTTGAGAATGATCAAAACACAAAAAAACCAGACCCAATCTGGTTTGGGTCA
AAAAAGTGGCAAAATTAAGCCAGAATCAAGCATAAAGGGACCAAAATGAGCAAATCTCAG
TTCAAGGCAAAGTGGATCATCTAGGCAGCCCCTAAATGACC

>Capra_hircus
TCCCATCGAGGCTTGCCCACGGGGCCTCTCGGGATTCCTCTCCCGTCGATGCCGGGGCCT
AAGACCTTGTGTGGAGTCGGTGCCGGAACCTGAGGATTCCTCTCCAGTGCAGACAGGGAT
AATGGGGTTCCCTGGAGTCGCCTCAGGGGATCAGGCCTCCTCTCGAATGGTGGCACGAAC
CTGGAGTTCCTCTCGCCTTTCCTGTGGAGAGCGCCTCCTCTTGCGATGCGACGGGAATCC
CGGGAATTCTTTCCCTCCACGCAGGCACACGAGCCCTCCCCACGAGCTTACAAGGCGCAA
ACGGGCCACCTCTGGATGTGAGCGCGACCCTCGTGCTTAAACTCGAGTGCAGACAGGGAA
AACAGGGAACTCCTGGAATGGCAGCAAACATCTGAAGGACCCTTGGGAAGGTCCACACGT
AACCTGTGAGTAGCCTCGAGACGCCCCAGCGGAAATGCGCCTCATCTCGACAGGAGACGA
GAACCGCCGGGATTTTCCCGACACGCGGAAGGTCCTCTCGACCTACGACGCGGACAACAG
GGACCCGCTCTGGTGGCCGCAGGAAACGCCAGTCCCCATGCGAGTTGCACACGGGCATCT
CGGGAAACCTCTCCAGTCGAAGCAAGGGCCTAAGACCCTGTGTGAAGTCCCAGACGGAAC
CTGTGATTACCCTCCAGCGCTCACACGGATAATGGGCCACATCTGAGTCTCCCCAGGGGA
GAAACTCCTCGTCTCGAGTGGCGGCATGCGTGCGCTCGACTCACGAGCGGGAACAGCAGG
GACACGCTTCCCGTCGCCTGCAGCAAAGGACCAGTGC
>Lithocarpus_balansea
GAAAAATATTTCCCAGCCAAATTCGTGAGCAAAACTTCAAACTTCCCCTTTTGAGCCCTT
ACATCGGCACGAAATTGGAATCCAAATTTCGGGTAACTCGGTGGCCCACATTCAAACGAG
GATAACTCTCCCGATTTTTATCAAAAAAATACAAAATTTGTGCTCAAATTCAAGCTCAGG
ATGTCTACTTTCTAAAACACGAAGGCCGCGTCAAAGAATTCCTCACGGTTCAAAAGTTAT
TGACGAAACGGTGGCCAAAGCACACTTTTCGTCACACTTCCTAACCGATTAACGCCCGCG
GATAACTACCCACTCACGTATTTTTTCCCCGAAACTTGATTTTGGGAAGATTGATCTCGC
GATATAG
>Lithocarpus_grandilofolius
CCGAAACCCAAAAAATGACCAAAATACCCCCGAAACCCAAAAAATGACCAAAATACCCCC
GAAACCTAAAAATGACCAAAATACCCTCGAAACAAAAAAATTACCAAAATACCT
>Lithocarpus_hancei
AGACTTCTCCTTTTGAGCCCTTATATCGGCACAAAATTGGAATCCAACTTTCGGGTAACT
CAGTGGCCCATATTCAAACGATAATAACTCTCATGATTTTTGTCGAAAAAATACAAAATT
TGTGTTCAAATTCAAGCTCAGGACGTCTACTTTCTAAAACACTTAGGCCGCGTTAAAGAA
TTCCTTTCGGTTCAAAAGTTATTGACGAAACGGTGGCCAAAGGTCACTTTTCGTCTCATT
TCCTAACCGATTAACGCTCGTGGATAACAACCCACTCACATATTTTTTCCCCGAAACTTG
ATTTTGGGAAGATTGATCTCGCGATGTAGGAAAAATATTTCATGCACAAATTCGTGAGCA
AAACCAC
>Lithocarpus_xylocarpus
TTTACGCCTTTATATCGGCACAAAATTGGAGTCCAAATTTCGGGGAACTCGGTGGCCCAT
ATTCAAACGACAATAACTCTCTTGATTTTTATCGAAAAAACACCAAATTTGTGTTCAAAT
TCAAGCTCAGAATGTCTACTTTCTAAAATATTAAGGCCGCGTTGAAGAATTCCCTACGGT
TCAAAGTTATTGTCGAAAGGGCGACCAAAGGTCACTTTTCGACACATTTCCAAACCGATG
AAGGCTCGTGAATAACTACCCACCAAAGTATTTTTTACACAAAACTTCACTTTGAGAGGA
TTGACCTCAAGATATAGAAAAAATATTTCATCGCCAAATCCGAGAAAAAAACCTTAGACA
TCAATG
>Lithocarpus_calolepis
TTTCGGGGGTATTTTGGTCATTTTTTGGGTTTCGGGGGTATTTTGGTCATTTTTTGGGTT
TCGGGAGTATTTTGGTCATTTTTAGGTTTCGGGGGTTATTTTGGTAATTTTTAGG
>Castanopsis_echinocarpa
AAAATTTTAACACATAATTCCATTATTAAATTAAATGTTAATGTCAACCTCGGCTAAGCC
AGGTCCTCGGACCTTCTGCCTTGGGAAAATTTAACATTCAATTTATTCATTTTTTAAGTG
TTAAATGTCAACTGGGGCTACGCCAGGTCCTTATACCTTCTGCCTTAGG
>Castanopsis_indica
GCTGCCAAGGCCAAGGCATGCAGCCAAGGCCAAGGCAGCCCCCCATGGGCGT
>Ficus_altissima
TATATATCAATTTCTAAAGCCTATAGCCTTCTCTAGCAACAGAAAATTGGTCGGGCTCAA
AATTTCTACAAATTTAAATCATTAAAACATGCTCTAAACATAATTCTAACGATCCACTTT
AAGTCGAAAAACACCTTCCGGATCACGATTGACGATACTCA
>Ficus_langkokensis
TTTTGCTGGTTTCTTGACCCAAAGTTGTTAGGATCTGGTTTTTTGTGTTTTGATCCTTGT

CAAACCTTGTTTAGGTCATTTACAGGCCACCTCACTGGTTTACACGACCTTGATCTGATT
ACATTGCATTTTGGGTCACTTTTTGCCTCATTCGGGCTTGA
>Trigonobalanus_doichangensis
GGACAGACCGAATAAGAGTGAAAACAAAATTTCGCACCGGGATCAGTTCTGCCCAAACCG
TGCCAGACATTCCTTAATAAATATTAAAAAATAGAAATAAAATTGAAAAAATTAAAAAAT
ATAATACTTGCATCATTCCTGGCCAAACCGGGCCG
>Myzus_persicae
TCATTTGGTTGAATAGAACCGGAGAACGAGAACTTTGAATATCCCGAAATTCGATTAATT
TTACCCATTTTCAAAATTGTCTCACGACCTGTGTATTTGGGCTAACCAAGTCAGAGTGGT
ATCAAAAGAAAGCTATTAACTTCCTCTTGAGTTTGGTGAAGGTTTGAAG
>Ficus_microcarpa
AAAAGAATATGCAGATGAGGAAAATAGAGGAGGTTAAAAAAAAAAAAAAAAAAAAAAAAA
AAAAAAAAAAAAAAAAAAAATGGTTGTTGTCCGAAATCTTCAATACTGTAGGGTTTAACT
AAGTTTACTAGGTACTGCTAGAAGACGAAATTTACTTCGGACTACTACTCCTTTTTTTTT
TTATCGTTCGGACCGGTGCTGCAAACTCTGTTAAAAAAAAATCCAGACCATC
>Ficus_tinctoria
TACCATTTTTAGAACACGGTCAATCCCGTTATGACCGTCTGTTGGGAAGAATCGAGAAAA
ATACAAAAAAAATCATTTCGGGCCATTTTTAACACACGGTCAAAACCGTTATGACCGTCT
GGTCATAACAGTTATGACCACAAATGGAAAAAAATCGAAAAAAATACAAAAAAAATCATT
TCGGGCCATTTTTAACACACGGTCAATCCCGTTATGACCGTCTGGTCATAACAGTTATGA
CCACAAATGGAAAAAAAGCGAAAAAAACTGGAAAAAATCTATTTT
>Anas_platythynchos
ACAACTGAACTCCCCAGGGGAGCCTTTACGCAGCCCCACATGCAACTGGACCCTCCGGGT
TCGCTCTTTCACAGCAACCCTAAATATAGCCCTAGCCGCATGGCAGAAGACTGAAAAATC
GGGCCCCAGAAAACGCGTTGCAAGCCCCTTTCGCCCATCCAAAAGTGCCCCCAAAGGAAG
GCTTTGGGT
>Harpegnathos_saltator
TATCGAAGGTTAAAGTTTTCCAAGCTTTCTCCGTCGATATCTCCGCTTCTACTGGGAGTA
GAGAGTCGTCACTTACTCCAAAATGTAGGGTTTTGAAAGGACTTTAATTTGGTACCAGTA
CCAAGCCTGTATCTCTAACCGTTTAGGAGA
>Camponotus_floridanus
AAGCATTCATTTCTAGTTAGTTCAGTAGTTCGCATCATCGCATGATATCCCGGAAAAAAG
CGTTTAATTCTCGTTTGTACGTCGTATAGGCGTGCGTTATTCGGATTAA
>Linepithema_humile
TTCTTCGTATGTTTCTGGTCTCAAATTAATTTATAACTTCAGTACTATCGAAATATATAG
GAAAATGAATTTGTTGCTCGTTTTAAAGTATCGAGAAGCGACACTGTCGTAACAAATTCA
TACAGCACACATTAGAAAAGTTATAATAGTAAAATAATTT
>Carica_papaya
GTCCGCAGGTTTATTCGCAAATGTGAGTCACGGCGCCGGGGCATTTTACTTGATATTAGA
ACGAGAACGAGAGGTGCAGTGTGCACGCACAAGGAAACAAATGACGGGAGGGGGAAGAAA
GGAGAAGGAGTGTATGTATTTGCAGCGCGAAATTAAACAGGTGCGATCATACCAGCACTA
ATGCACCGGATCCCATCAGAACTCCGCAGTTAAGTGTGCTTGGGCGAGAGTAGTACTCGG
ATGGGTGACCTCCTGGGAAGTCCTCGTGTTGCACCAATTTTTTTTCAGATGGGTCGTTCG
ACGTTACGCGCGACTGGAATTATG
>Boechera_holboellii
AGAACCTTGTTCTAGAAGGATAATTAGTAAGAACCTTGTCCGAGAAGGATAACTAGTGAG
AACCTTGTTCTAGAAGGATAACTAGTG
>Mimulus_laciniatus
ACATAAGTAAAAGTACAAAAGCATGCACAAAAGTACTCACGCAAAAGTGCATTCGCAAAA
AGGATCATGAAAACACAAACTACAATCCTAGGACATTAATACAAATCAACAAACATGCAC
AAACACACTCCCTCAAATGGACATGCACAAACGTACATGTAAAAAAACATGCGCAAAAGT
GCATCCGCAACAACGTTCTTAAAAACACGAACTACAATACTATGACTTTAATACGCATCA
ACAAACAACAGCAAAAACACTAGATAAAAAAGATACAATGGAGTGTAGAAATAAAACTCG
ACGTTTACGTTTACGTGGATTTTTGTTTGGATTAATTTATATTTCTGTTTTAATTGGTGG
ATTTGGTATCTTATAGGTACAAATCGAAAATTAGGTGTCCAGGAAGCAAAAATGATCAAA
GGGTAAGAAAAGAGTTGCAATTAAGAAAATAAAATCAAATTAGAAGAGAGAATCCCTCAA
CATCTTTGGTAGTGTCCACGAAGTCGAGCATAACTCTCTCATCCGGACTCCAAATCAACT

GGTTCCGGCGGCATTAGAAAGCTATTTCAGCGGGCTACAATTCCCGTCTAACGTCAAAAT
TCCAAATTCGGACTCGAACATGGTCAAAATTGGATAACAAAAGCGATCATGAAAATACGT
ACTACAATTCAAGGACATGAATGCGCATCAAGAAACGTGCACAAAAACACTCGCTAGAAA
AGATACGC
>Mimulus_guttatus
CTTTGATCATTTTTGCTTCCTGGACACCTAATTTTCGATTTGTACCTAAAAGATACCAAA
TCCACCAATAAAAACAGAAATATAAATTAATCCAAACAAAAATCCACGTAAACGTAAACG
TTGAGTGTTATTTCTACACTCCACTGTATCTTTTGTATCTAGTGTTTTTGCTGTTGTTTG
GTTATGCGTATTAAAGTCCTAGTATTGTAGTTCGTGTTTTTAAGAACGTTGTTGCGAATG
CACTTTTGCGCATGTTTTTTTACATGTACGTTTGTGCATGTCCATTTGAGCGAGTGTGTT
TGTGCATGTTTGTTGATTTGTATTAATGTCCTAGGATTGTAGTTTGTGTTTTCATGATCC
TTTTTGCGAATGCACTTTTGCGTGAGTACTTTTGTGCATGCTTTTGAACTTTTACTCATG
TGCGTATCTTTTCTAGCGAGTGTTTTTGTGCACGTTTCTTGATGCGCATTCATGTCCTCG
AACTGTAGCACGTATTTTCATGATCGCTTTTGGAATCCAATTTTGACCATGTTCGAGTCC
GAATTTGGAATTTTGACGTTAGACAGGAATTGTAGCCAACTGAAATAGCTTTCTAATGCC
ACCGGAACCAGTCGATTTGGAGTCCGGATGAGAAAGTTATGCTCGACTTCGTGGACACTA
CCAAAGATGTTGAGGGATTCTCTCTTCTAATTTGATTTTATTTTCTTAATTGCAACTCTT
TTCTTACC
>Mimulus_dentilobus
ATGAGAAAGTTATGCTCGACTTCGTGGACACTACCAAAGATGTTGAGGGATTCTCTCTTC
TAATTTGATTTTATTTTCCTAATTGCAACTCCTTTCTTACTCTTTGATCATTTTTGCTTC
CTCGACACCTAATTTTCGATTTGTACCTATAAGATACCAAATCCACCAATTAAAACAGAA
ATATAAATTAATCCAAAAAAAAATCCACGTAAACGTAAACGTAGAGTGTTATTTCTACAC
TCCACTGTATCTTTTTTATCTAGTGTTTTTGCTGTTGTTTGTTGATGCGTATTAAAGTCC
TAGTATTGTAGTTCGTGATTTTAAGAACGTTGTTGCGAATGCACTTTGGCGCATGTTTTT
TTACATGTACGTTTGTGCATGTCCATTTGAGCGAGTGTGTTTGTGCATGTTTGTTGATTT
GTATTAATGTCCTAGGATTGTAGTTTGTGTTTTCATGATCCTTTTTGCGAATGCACTTTT
GCGTGAGTACTTTTATGCATGTTTTTGTACTTTTACTTATGTGCGTATCTTTTCTAGCGA
GTGTTTTTGTGCACGTTTCATGATGCGCATTCATGTCCTCGAACTGTAGTACGTATTTTC
ATGATCGCTTTTGGAATCCAATTTTGACCATGTTCGAGTCCGAATTTGGAAATTTGCCAT
TAGACGGGAATTGTAGCCAACTGAAATAGCTTTCTAATGCCGCCGGAACCAGGTCATTTG
GAGTCCGG
>Sorghum_propinquum
TCAAATGGAAGCTCGCTTTGGTCTGTTTGGAGACAGTGCTAATCTCGATGCAAGATAGGT
GCACGGTTTGCATGGAACATACCATATGCTAAGAAATCAATTTGGACGCACCCGATGGAA
CTCCTAGATGACGTGTG
>Panicum_hallii
AAAGTCGATGCAAAACTGGCCGAACTGGTGCCATTAACGCACAAGTTCGCTAAACAAAGT
CGCGTCGGAATTTTTCGCAACGAACGCACCCGATCCACTCCATTGGACCCAAAACTCATG
TTTTGGGGCGTTTCGGACCGTTTCGTTACTGCACG
>Staria_viridis
TTGCGAAAAAATCCACCCGAGTTTCGCTACCCGGCAATAGTGCATTCGGGTGCCGAAAT
GCACCCGTTTTGCATCATTTTTCGTGCCGGAACCGAATGCCCAAAAACACTCCCAAACAT
GTTCTAGGGTATAATTAGGAAGATTGCATGCGTTC
>Phaseolus_vulgaris
TAAAAGAAGTATTAAACATATCAAAACACAATTTTAACACATGAAATTGTTTTTCAAAGA
TATCATAAGTGTTTCAATCAATTAATAGACTTAAATTGTAAAAAACATATCAAAACACAA
TTTTAACACATGAAATTGTTTTCAAAGATATTCATAAGTTTTAAAACAATTAATAGACTA
ACATTG
>Arabidopsis_thaliana
GGTTGGTTAGTGTTTTGGAGTCGAATATGACTTGATGTCATGTGTATGATTGAGTATAAG
AACTTAAACCGCAACCCGATCTTAAAAGCCTAAGTAGTGTTTCCTTGTTAGAAGACACAA
AGCCAAAGACTCATATGGACTTTGGCTACACCATGAAAGCTTTGAGAAGCAAGAAGAA
>Cajanus_cajan
ACTTGCTACACCTGGGGAGACTAATAACCAACACAGATGCACAACATAGCATGTAATTGG
TTTACTGTTCATTGGTTCTCTCTAATTCTTCAACTGACTT
>Lemna_gibba
CACACGCACGTATGCACACACATACGCACGCACACACGCACGTACGCACGCACGTACACA

CGTA
>Medicago_truncatula
ATAAGGTCATTTTGAACGGTCGGATTGAACGTGGCTGGTGTCGTTCACGATCTAGGCACG
TTTAGGTCCCCGCAGTGAACTAGTTCCTAAGTTGACTAGTCAATTAGGTGATAGTTCGTC
CGGATGACGTACCTCCGTGAACCCGATCTGAGAAATTCAAGTTTCTGCATCCTTCTATGT
TTG
>Solanum_pimpinellifolium
CCCCAAATGCATCAAACCAACCAATGGGAGACCTACATCTCCTACCATACAATGCTTCAA
ATGGAGCCATATCAATGCTTGAGTGATAGCTATTATTGTATGAAAACTCCGCTAAGGGTA
GGAAGCTATCCCAATGACCACCAAACTCTATCACACACGCACGAAGCATATCCTCCAACA
CTTGAATCGTTCTCCCAGACTGACCATCGGTCTGGGGATGGAACGCAGGACCGCCCCCCC
CCCAAACCCGACCCACCCCCCCCCACCC
>Neolamprologus_brichardi
CGGGCCGCCTGGCCACCCTTTATTCGGGTGTCCGCTCCCCAGGCTCCGAAAGCCTTGCCA
GCCCTTGTGATCGGGTGGCACCGCCCACCAGGCGTGTGAAAAAAAGAAGAAAAAACCCGG
AC
>Mustela_putorius_furo
AGGTTCAGTTCAGGCTTACGGTTAGGGTCAGAGTTAGGTTCAGGGTCAGGGTCAGCGTTA
GTAGGTTCAGGTTCTGTTTTATGAGTAGGTTCAGGATCAGGGTTAGCTTTAGGATT
>Pundamilia_nyererei
CAGTGAGAAACGCACTGTCTTGGCGAAATAAAGCGTTTTTGTACAACTTCATATAAATCG
CTGTAACTTTTGATAGAAGACTCAGACAAACATGTTTATGGCTTTATCTTATAGAACTCA
ATATCCCCGTGCTGGGCAAACAGGTTTTGCAGCCGTTTGAGCTAAGATTTTAAAATTATT
CACATAATGAAAACCTATACTTTGTTTCAGGCGAGTTTCCCATTCAAATGCATGT
>Zea_luxurians
TCTCCACCAGAAATCCAAGAATGTGATCTATGGCAAGGAAACATATGTGGGGTGAGGTGT
ATGAGCCTCTGGTCGATGATCAATGGCCACACAACCCCCATTTTTGTCAAAAATAGCCAT
GAACGACCATTTACGGATTTTTGACCAAGAAATGG
>Theobroma_cacao
AAAAAATGTAAAAAAATTCAACTTAAAATCAATAATGAGATTATAAAACCATTTAAAGCG
AGTAATAAGGCTTACAAAGGCATTGGTGACTATAAATGGACTTTTCTTGTCAATGGATGG
GATTCGCTTCACGTTTCTCGAGGGAAACATCCAATTATAATCTGTTTTAAC
>Salix_purpurea
AAATCCAAGGCTAACACCCCCTCCAAAAACCAAGTTCAACCATTTTGGGGGGAAAAGTCT
ATTACCCATAAATTTGAAGTCTAATACCAACTAAAAGGGATTGGATATGAACAAGGTTAG
AAATCTAAGGCTAATACTCATTTAAAATGGGGCTCGTCGAGTCAGGACCAATTTGTCCG
>Branta_leucopsis
CAGGGAAAAAAAAAAAAAAAAAAAAAAAAAAAAAAAAAAAAAAAAAACGAAGCAAAG
>Aegilops_tauschii
TTCTGACATCATTTGTTATTTTTCAGGCATTTACCGAATTATTTAAGAGCTAAAAGACCC
TAAAATTGAAAAGCACTACAAAATGAACTCTGAAAAGGTTGAAAGTTGGCATGGTATCAT
CATTTCATCCACATAGCATGTGCAAGAAAGTTGAGAGGGTTACGGCAAAAACTGGATGCA
CTTCGTGTACAAAACGGACAATCTCTTTCAAAGTATCAGGATTTCATACGGAAACTCGTC
TGTTACAAAGGGATTTCATTTTTTAAAACTTATTTGAACTCCTGACTTTTTGTGTGTTCA
AAATGCACCATTCAAAGCCACATCATCATTTTTCAATCCT
>Lottia_digitalis
CAATAGGGTTAATCCTTGGTTGATTACGAACACTCACACCAAGTTTCGTCAAAATCCGTC
AAGAATTGTGCTTTCTAGAGTGTTTACGAGCTAAAAATTGAACGCGTTTTCAAGAACAAG
GGCAATAATTCATACTAAATATTTTTCGATTTCGCTGATTTT
>Gallus_gallus
TGATTTTCGGGTTAAATGGGGGATTCTTGAAGAGAAAATGCA
>Mayetiola_hordei
AACTTCATTTCTCACTGATCTAATGACATCGGTTTTGTAATTCTAGACATTTTTCAAGGC
AAATGCTGATTGGTTATCTCTCAGAGAATCATTTCTGAATTATGTATATAGCTATCGGCG
ACCCTGAATTTCCAATCCAATCATATATTGGTCATAGATTTGCTTCAAAAGTTTTGAAAA
TATTCGAAAAACTTCATTTCTCACTGATCTAATGACATCGGTTTTGTAATTCTAGACATT
TTTCAAGGCAAATGCTGATTGGTTATCTCTCAAGAATATAGCCATCAGCGATTCTGAATT

TCCAATCCAATCATATATTGGTCATAGATTTGCTTCAAAAGTTTTGAAAATATTCGAAA
>Eucalyptus_globulus
CAAAAAATGGCCGGAAAGGGAATTTTCTATGGAAAAACAGGGAATTTTCTCTGTGGAATC
TTCGTTCTCTTGTTTCGCCGGGCGTTCAGGGTGCTGTGATGTCCAAAATGATGGGTCGGA
CTGATCTAATGCTTTTGGAAGCCATGTTTCCCAACAGATTTCCAACTGTCGGGGTTTTTC
AGC
>Lottia_scutum
CAAAAAATGGCCGGAAAGGGAATTTTCTATGGAAAAACAGGGAATTTTCTCTGTGGAATC
TTCGTTCTCTTGTTTCGCCGGGCGTTCAGGGTGCTGTGATGTCCAAAATGATGGGTCGGA
CTGATCTAATGCTTTTGGAAGCCATGTTTCCCAACAGATTTCCAACTGTCGGGGTTTTTC
AGC
>Chinchilla_lanigera
CTAAGTTCACTATTCCCAGGAAGCACTCACTGTAGGAAACCTATACCACCCACAGGAAAC
ATTCTGCCTTGCTCAGGTAAGGGGGGATTCCACACATTCTAAGTTGACTATTCCCAGGAA
GCACTCACTGTAGGAAACCTATACCACCCACAGGAAACATTCTGCCTTGCTCAGGTAAGG
GGGGAAACGCACTCACTCTTGTGCTCATAGGCGCCTATGTGAAAGGAATGCCATGGGTGC
ATTGCTCTGTGAAAACAAGCAGGGAGAGCATAGCCAATGTAGGCCCATACTTAGTTTCAC
TAGGGAAGCTCACCACAGCACTTAGAAGTTTGGGAGTGTTTCCGGGAGTTTGGGGCAGAA
AGTTGTTCTTTTCGTTCTAAGTGGGATTTACTGTTTTCTAAGGGAGCATGTATAGGACTG
GTGTGCGGTGTATTCCACCCATT
>Trichechus_manatus_latirostris
TGCTATCACAAATGTTCTCTCAGTGGGAGAAGGCAGTAGAGAGAAGGCCTAGCAGGAATT
GGAATCCCTGTTTCAAAGGGAACTTTGCATAACAGGAATGAAGGTATTGCTAGCTTACAT
AGCCAAACAGGCAGATTGTTTGCCTTCAGCTAGAGAACAGCAGAAGAGGTTCAGTTTTGC
TAACTAAACCTTGTGTGCTTAACACGAGCATGTAGCTTCTTCCCTGCCTGAAAGAGAAAG
CAAGAGCTTGCTGCTCTCAGTCTGGGGATGAAGGGAAAAAGATTCACTTTCTGTGTTTGA
AAATCCCTCTGTGTTACATGCCTAAGAAAGTTGCCTGTGCATTGTCTACCTGGATAAAGT
GTATGACTTTTCTCCCTTTCAAGTAGTTGAAGCCGTTCAGGGTTTGCAGCATTCCGGGAG
GCATTCTGTGCTGCGCGGCTGAGAATACCGCTTTACAGACGGTATCGCTAAAGGAGAAGA
GAAACCGCGTTTCTGCCTTTCTGGAACACGAATGAGGCAAAACCGTAGTTGTGCTGTGTA
AAAACTTTGTCAGCAGCAAGTGTAAGTTCCCCATTCACCACATGAAAGAGAAAGCAAAAT
ACAGCGTTCTCCTTCATGCTGCTTAACTGAATCTACTGAAGTTTTCAGTTTTGTTAGAAG
AAGCAGTCCGCCCACCACAAGC
>Leptonychotus_weddellii
AGAAATATAGAAGATAAATGAGGCAGAAGAGAGAAGC
>Saimiri_boliviensis
ATTCCAGAGTGGATGGGAGAACAGGCTGTAAAGAGAAATACCTTGCTCTAAAACCAAAAC
GGAGCTATCTAGCAGAATGGTTGTCAATGTGTGTATTCAACTTCCAGAGTTAAACTGATG
TGTGTTTGCAGCAGTTCAGAAACCCCTTTCTTTGGAGAATGCAGAAACAGTCATTTCCAGC
CCTAGAGACGCATATAAGAGTTAGCAGTAAGAATGCGTTTCAAACACGGAAAAAAGATGT
GTGTAAGCACGCAGTTAAGAAACCCTTTCTTAGGAGAATGTAGAAACAGTCTCAGCGAGG
TAGAAACACGTTTCTTCTAAAAGCTGCGTTTGGAC
>Cricetulus_griseus
TTAGGG
>Drosophila_bipectinata
CCTGCCGAGGCGTGGAGTGCCCGGCCGATCCCCTGCCAGCTGATACCCCGCCAATACGTG
AAGTGCCTGGCCGATCTCCCTGCCAAGGCCTGGAGTGCCCGGCCGATCCCCCTGCCAAGG
CGTGGAGTGCCCGGCCGATTCC
>Drosophila_elegans
GTTTAGTTCTTCGACATATAGCAATGGTTAAATATTTCAGAATTACGGTTTAAATTTCAT
CAAAATCGGACGACTATATCATATAGCTCCCATAGGAACAATCGAAAAATAAATGAAAAA
AATTATAACTTTTCTGTTTTTAACTTTTT
>Drosophila_ficusphila
TTTTCGAATTTTTGGTCAATATTTTGTAATTTTTTATGACCCCCGACCTGTCAAAATTTG
CAAAAAATGGGTTTGCAGAAAAGTGACCAGATCCCAGCACTGCTTAGCCCATAACTTTTG
AAATTTTTACCCGATTTAAAAGTGGAATACCTCTCTGAATTTGTTATTAAAATATCTATC
TAGCTGCATTATTGGTTTA

>Drosophila_kikkawai
ATACTCAATTGTAAATTTGTAAACAAAAAGTTCACAACTCAGCTAATAATGCATGTATCC
ACTTCAGAATTTGTATTTATATTAGTTTTTACAAGGCTAATACATTTGC
>Drosophila_rhopaloa
TTTCCGAGGACTTGTAACAGAGTGACTCAAAATTGGACTTAGAGAGTCCATAACTTTACC
AAACTTAAGCCGATTCCAAAGTGGCATACCTCTAAAGACTTCTGATTCGATTCTCTAAAA
ATCTGCATTCAAATTTTTCAATTTAACGTTGATCAAATTTTTGACCCATTTTCATGTCAG
TTATTTCTAA
>Drosophila_takahashii
ATTGGAATGTCCGATTTAAGCCATAATTGCTGAGTTTATAGGTCTTGATGAGTAGAACAA
AGTTGCCATACAATCCTTCTTTCTATCTCTTGTAGTTTTTCAAAACCGTCTTATATCTGA
AAATAAGGAAAAACATGGTAACGGATGAAAATGAAAAGACAAGTAGCTAAGACGGCCAAT
AACTTTTG
>Drosophila_eugracilis
GGTTTACCATTTTCCTCCTTTAAGTGACAGTATTATGGCTATAACTAAGCCAATAGTAGC
CGAATCTGGAATGGCATAGTCGTTGGATTCTTTATTAATTTAAACTT
>Drosophila_biarmipes
TTTGCCATCGATCTGCATCATTTAAAAGAAAATTTATTTTTTGACCCACTTTTTCATTTT
TTACAAGGAGGTGTGTCTTAATTTTTTAGAATTCGGATTTTAGCCAAAAAACACATTTTC
TTGCGGTTTTTCAATCGTAGTTTATCCAATTTAAGGAGTACAGCTAAAAGACCGACTGTT
TTGGAAAGCTGGCTTAA
>Boechera_stricta
ATTTCATGAACTTATTTTCCTCCTTCTCGATTCTTATGAGTTATCGCTGTTTTCAATGGA
AGGTCACGAAATCTCGAAACGCAATGTCATAATAAAGTTTGATATATTGAAACTTACACC
ACTTATGAGCATATTTATCCCTTAATTCACTTTGGTGGTTTTCAAC
>Cannabis_sativa
TCAAGACAGCTTTCAAACATCCTCAATCAACCAACGTGAACGCACCCAATTGAGGGTGGT
GACACCGGATGATTTCAAATGAGTGTCCCCCATCACTCCGACAATGAAGTCGG
>Clonorchis_sinensis
GGGTTTGTGATGCGGTTGCCACGTGTTGTGTGACCGCGACGAGCG
>Ctenopharyngodon_idella
ATTGCGAGATATAAAGTCAGA
>Acropora_digitifera
GAAAATGCAAAAATGGCCACCATGCAAAGGCTATAGCCCATGCAAAATCGTCACTTTGGG
TCAAAAATTAAAAATGCAAAAAACATGCCAAAAACCATTCTACAAATCATTTACAGTTGT
TCTGTGCAAAAAACCGCTCCAAAAAACACCAAATATTCGAAAAATGAGAGCATTTT
>Daubentonia_madagascariensis
GGTTAATCACTGCTGAAAAACCGCGTTTACACTGCCAGAGAATCACATTCGCAGCAAGGC
GTTAGAAGAGGCAGGAGAAGAAAACATCGGTTTTTCTGCCAGCCAGCCGCTTATGGAGAG
TGCACGGGAGTTTCACAGGAAACACTGCCTTTCCCAGAAAGAAAGCACCTACTTCTTAGA
TTGATTGCAGGCTTGCTCTATTCTGTGAAAGGAAGCAGAATATACACTGCCTTCCTCCTA
GCTTCCTCCTAGGATGAATATTGAAACCTG
>Dirofilaria_immitis
ACAAATTCATCGTATATATATATGATTAGACGGGTCACAATGCATACCTATGATTAGACA
AACACAGACGAGTCATAATCCGTATCTATGATTAGACAAATATATACGACAAATTCATCG
TATATATATATGATTAGACGGGTCACAATGCGTATCTATGAGTTATACAAATATATACG
>Perdix_perdix
TTTGGGGAGAAAAGTGAAGATTCGAGCAGAAAAATGGGGATTTGAGGGGAAAAATGGGGA
TT
>Heliconius_cydno
AAACCATGAAACATGTGTTCAATTAAATACTTTTAAGAGTCTAAATTTCAAATTTTATAA
AAATCGGTGACGATTTAAGAATTTTTCCATACAAACTTTCAACCCCTTATTTGCACCCTT
AGGGGGAGAATTCTAGA
>Heterocephalus_glaber
AGGGGCTTCTGGGCCACCATCCCTACTGCTTCCCGGCCCAGAGGA
>Heterodera_glycines
CGTCATTTTTTTACGGAAGAATAACCGGAATACAAATTAATATTTTCAATTAATTTTTGG

CACTATACTGCTATAGTGGCTTGGCCTTGGCTTGGAATTTTTCTGTAGCTCAGCTCCCTG
ACAGTCGTTTTTTGATTTGAAGGTACCATTCGAAAGAGCTCTTTTTCTAGGCCTAATGGC
TAAGGTGG
>Lepisosteus_oculatus
GGCAGTAAAACCACATGTAACAAAACGGGCATAACAGGCACACTTTTCAAAACGTGTTTT
TCAGCCCGAAATAAACGCGTTTTTTCAGACAACTGAATACCCATGTAGCAAGCAAGGCTC
AGAGAGCCCACACACTCAGATCTTAAAGTGTTTTTCAGCAGAAATAAACATGTTTCTGCA
AGTTTGGTCAACCCCATGTAAGTTATGGGGCCAACAGAAATAAACAAAACGTTTGGTCAA
CCCCAAATAATACGC
>Leucoraja_erinacea
TGGTATGGTCAGACGTGGGGGCAAGCCTGCCGCTGATAAAGGAAAGGACAATCGTGATTA
TCTTGCTGCCGCATTCCCTGAGCTTAATGCTGGGGACCGTGCTGGTGCTCATGCCTCCTC
CGC
>Linum_usitatissimum
GTTCTGTCTATTTATAACTCTTTTATGTCGGTGATATACACTTTACGAGTATGTTATGCG
TCTTTTATTTATGTCTTTGTTACGTCACTTACGATTAGTTGAATTCATCATC
>Lytechinus_variegatus
TTGAATTACCATCATAACTTTGAAAGTTTATGGATCTAGTTCATGAAACTTGGACATAAG
AGTAATCAAGTATCACTGAACATCCTGTGCGAGTTTCAGGTCACATGATCAAGGTCAAAG
GTCATTTAAGGTCAATGAACTTTGGCCGAATTGGGGGTATCTG
>Manihot_esculenta
TTTGGGATGGAAGCTATTAGGCCGAAATCGGGCACCGGATGTAACATGGGTGATAGACTT
GGCAGTT
>Musa_acuminata
GCTGAGAAAAGGGCAGTCAAATGCCAAGAAAACAGAACGAATACCAGGTGCTAAACCATA
GCAGATTCTAAACTTGGGGTTGATCTCTTTAGGGGATCGGCCTCCTTGGAACTCTATAGG
GGGAATTACTCCAAGTGGCTGTCAAAA
>Patiria_miniata
CTGACAAATCGGCAAGGTTCAGACTTGAAACTTTTGGAGTGAGCCCCAGACATAGGTACA
TGATGCCCCCAGTTTCGTTTTTGTGCAGTAAGTCCCATAGCAAGGGCTGTCAGCGCCAGC
CGACATGGCTCTCATGTGTCGCGGTCTAAAGGTACCTTAACATGGATGGATTTTGACAAA
CTATAACTTGTTGGATGGCGGATGAATGGCTCAACAAG
>Phoenix_dactylifera
TCGACTCGCCTCTCAGAGACGAAAATACCGTCTTCTGTCTTTTTGGAGTTGCGCTGTCTC
GGAGTCGACTCGAACTTTGCGGGAGTCGACTCGGCTCTCAGTGTCTGAAATTGGCTCTCT
GACTTTTTGCTTGTATTCCTCTGGGAGTCGACTCTAGCTTCCTTTGGAGTCGACCTGCCA
ACCATCGGAGTCGACTCGAGTTCCTCAGGAGTCGACTCGGCTCTCAGAGTCCAAAATAGC
TCTCTGACTTTTGCCTTGTTCTCCCTAGGAGTCGACTCGTACCACACTGGAGTCGACTCG
AAAAGCATCGGAG
>Pogonomyrmex_barbatus
ACCTGACGGTCTCGGCGGTACCTCCGCGCGCGGCGTGTTGCTTTCGCTCGCGGCGGT
>Radix_balthica
AATAATCTAAAAAATCAATAATTTAATTACGGTACATGCGATCGATCTGAAATTTTGATA
CATTGTTTTTATCATCGGTACGAAACTGCCTGCAAAATTTCAGCTCGCTAGCTTACGAGC
AAGTGGGTTAAAAATCGATCAAAAGTTTTGGCGACAAGCAAGCAAGCAAGCAAGGTCGGC
CGCAGACACCACGAGTTAATATAAAGGATTTAATAAAAAAAACCCGTAAAAAACCAAGTT
TTTGACTATAAATCCTTTATGTACGAGACGTAACTAAAAGAATGTTCATTATAATACGCA
GCGCAAGACCATCCAAAATACAAAATTGCAGTTGTGTCGTAAAACATGTTTAATATAATG
CGCACCGATCAGGAATATGTTTATAAAAAGAGCTGTCAATAAAATACGCACCTTATTGCG
CGACAGAAAGGTTGTTCAATATAATACGCACCGCGACGCTCTATCTCAAAAAATAGCATG
ATACATAGGAATAAATAA
>Ovis_aries
TCCACAGGAAAGGCGAGAGGAACTCCAGGGTCGTGCCACCATTCCAAGAGTCCCCCAGAT
GTGTCAGTCCATTCCAGAGGAACCTGTTTTCCCTGCACTGCCTTGACGTTCAAGCCGAGG
ATCGACTCCCACCACGTGTGCACGTGGGACAGCCCTGTGGGAAAGCCTCGTGGGAAAGCC
TCGTGGGAAAGACACGAGGGAAAACCATAGATGCTTTGATCCACGGGGCGGACTGCGTGA
CACTGCTGCTACCGCTCTGGAGGAAAGCGCAAGTGCATGCCCGCATTCGAGACGAGGACT

GACTCCCCTGGGGAGACTCCAGAAGTACCCCAAGATCCATGTCAGCACTGGAGAGGAATC
CTCAGGTTCCGGCCCTGAGTTCACACAAGGTCTTAGGCCCCGGCATCAGCGGGAGAGGAA
TTCCGAAAGGCCCCCGAGGAACTCGCATGGGGACTGGCCTTTCCTGAGGCCACCAGAGCG
GGTCCCTGAGGGCCCCGTCGTAAGTCGAGAGCACCTGCCGCAACTCGAGAAAATCCAGGA
GGTTTTGCCCTCCAGGCGAGATGAGGCCCATTTCCGCTGAGGCTTCTCGAGGCTAATCAC
ATCTAACCCCTGGAACTTCCAAAGGGTCCTTCACACCCTTGCTGCAACTCAAGAAGTTCC
CGACACACCCGTCTCCACTCGAGAGGAAGCACGAGGGTCCCGAACACATCCAGGGGAGCC
CCGTTTCCGCCTCCGAGCTCGAGATGAGGGATCCTTTCCCTGTTTCGTAGGGAAAGAATT
CCCGGCGTTCCCGTCGCATCTCAAGAGGAGGCGCTC
>Eucidaris_tribuloides
TGGTTTTCTACCATATATGCTTGCATCTGACAGTATTAAGAAGAACACCAGACCGCAATC
AAACAAAACGAATGCATCACTATCTGATGAAACGAAAAAATATGTTTACACCCACTGGGA
AGTAGATTTCAGCGAGATATTGTCATTGGTAGAAGATAGTTAGGGATAGGGTAGAGTTAG
ACGTTAGCATGTAGATTAGGGTTAGCGACAAGATATGCTTGCATCTGACAGTCCATTGAA
GAAGATTATCAGACCGCAATGAAACAAAACGGATGCATTACTATCTGATGAAACGAAAAA
ATATGTTTACACCCACTGGGAAGTAGATTTCAGCGAGATATTGTCATTGGTAGGGTTAGG
GTTAGGGTTAGGGTTAGGGTTAGGGTTAGGGTTAGGGTT
>Oryza_brachyantha
TTGTATGAATGGTGAAGTTGTTGATGGAATCCAAGTGTACCCTTCGTGCTCGAAAATTTC
GCTACCGAAGTTGTGTTCGAGCACGAAGTGTAAACCAAGTGGATACACAATTTGTGTAGT
TATCCCAAAGTTCATAGTCGAATCTTGGTAGACT
>Anopheles_merus
CTTTCCCATCCATCCATATGGCCATGGTTGAAATTTTGACGAAAATGCAAGGTCAACCAT
TATCGTTCTTATATCATTGAGGCTTCTTGAATAACCAAACCAAAGCCCTGGAACACAATA
ATAAGGATCTTGGCGATGAAAAAGTCTCAAAATGGGTCATAGTACCCTTTATACACATGC
TAAAATTGATAGGTTTTCCCGAGTACAATGCATTCAGAGGCCATTTACTAGGCTACCAGT
GAGTCTTATCTTTAAACCTATGTGCGGATCTAGAAGACCACTTCAATACCTTTCCAAAAC
TGTCTTAATCGTCTCGCTAGGACACATGGTTACGAAGTTATGGCCATTTGAAGGAATGTC
CAA
>Zostera_marina
CGCACTAGAGTGCATTCCTTGGTCGGGATCACACGAATTGCACGCATGAAGTACTTAGTC
GGGAT
>Miscanthus_giganteus
ACACTAACACTATCTCCAAACGGACCGAAACGAGATTCCACATGACCCACGTCACCTAGG
AGTTCCATCGGGTGCGTCCAAAATGATTTCTGAGCCTATGGTACGTTTGGCGCAAACCGT
GCACCTATCTTGCATCA
>Hordeum_vulgare
TCATGCCTCTAGTTGGCTAGCCAGTTGATCAAAGATAGTCAAGGTCTTCTGACTATGAAC
AAGGTGTTGTTGCTTGATAACTGGATCACGTCATTAGGAGAATCACGTGATGGACTAGAC
CCAAACTAATAGACGTA
>Macaca_fascicularis
AAGAAGCTTTCTGAGAAACTGCTTTGTGTTCTGTGAAATCATCTCACAGAGTTACAGCTT
TCCCCTCAAGAAGCCTTTGCTAAGACAGTTCTTGTGGAATTGGAAAAGAGATATTTCGAA
GCCCATAGAAGACTATAGGGAAAAAGGAAATATCCTCAGATAAAAAAGAGA
>Thellungiella_parvula
TTTGAGAAGATACTAAAATCTAGAAACTAAAAGACCAAGAGTGAGAACATGATCATGAAA
GGAGTCTCTTAGAGATCCTAAAGGGAGTTTTAAATCCCAAAATCCTTACACACATACTTT
TGTTGAATCAAACAAACTAAATGTATGGACACAACTAGGCAAAAGGATTAGGGAATCCTA
GATGCATTAGGACATGCATATCTAGTTGGTACTCATTGT
>Bison_bison
AATGGAAGATTGGACTTCCCTGGGCCAAACACAAGAGGCATCCTGAATTCCCCGTCGTAA
TTCGAGAATCCTGCCGACACTCGAGAAAATCCACGTGTTCCCCCGTCATCGCAAGATGAA
GCCCTTTCCCGCTACAGTGTCTCAGGAGAAGTCCCACGTTAGGTATTGGAGGTCGAAACG
GTACTTGGCACCTTGATGCGACCACAAAGTGCCCCGACATCCCGGTCCTCCCTCGAGAGG
AACACCGAAGTTTTCCGGCACCACTTCCTCTGAGCCCCTTCTACCCTCCTGATCTGGACA
GGAGGGTCGACTCCCCTGCTTTGTCTGGAAGGGGTTCCCGACCTTCCGGTCGCACCTCAG
GATGAGGCCGGGTCTCACGACGACATTTCAGACGTGGCCTCGTGGGTGGTTCCACATTCC

GAAGGACCCCGATTTCCCGGTCCCCTCTTGATAAGAACCCGATGCCCGGACACCTCTTCG
AACCTCCACCCTGTGAATGAAGTCAACACGAAAGGGCAGTGACTCGCCCGTGCATCGTCG
GGAAAAAACCCCCAGGTTCCAAATACCGCTCGACAAGTGGCCTGTCTCCCCGGGAAACAC
CTCGAGAGGCAAGCGGAGTTCCATGCCTCACCCAAGACGAGGCCTGACTCTCCCTGTCCC
AAGTCTGCAGGGACCTTGCGATCAGAGTCTGAAGTCAGAGGAACCCTGAGGTTCCTGCCT
CAACTGGAGATGAGGCCCTCTTCCAATGCACCAAACCCAGTGGGAGTGCCGAGAAGCCCT
CCCACCTCCAGTTTTCCCTGACTTCTCAGAGCCACCATGAGAAGCCCCCTGAGGTCACCT
GCACAAGTCAAGGGAAGCCAGGTTTTCTGCCTCAACCCGAGAAAGACCTCGAGAGACCTT
CTTCAACACGTCTCGAGCCAGATTCCCCTAACAGTGACTCGAGAGCAATGACGCGCTCCC
CCTCGCCATTGCGCATGGAGACCCCGACTTCCCTGGCGCCCACGAGAGCTCACTGACCTC
GCCGTCGTACACTAGGAAAAACCGCACACTGTGGCGCCAGCTCGAGAACAACCCTGAGCC
CTCCCCATCATCGCGAGTTGAGGGCCTTCGTCTCCTGTATGGCCTAGAGACCAATCTCCT
CGACTCTCTCTCAAACGCCTCAGGAGGCTTGACTCCCTTTAGTCCACCCAGTGAGCTCCA
AGAGATACCCGTCGCGACTCGAGAGCAGAGCGGGGTTCTTTGCTTCCACTCGACATGAAT
GCTGTCTCCCCGGGTGCGTCTGGAATTGCAACCCTGAGATCCCTTTCGCCCCCTGGAGAG
GAACACTGGCTTCTGGACACGAAGCCTAGATGAGGTCTATTGGCCCTGCAGTCACTCGAG
AGCAATCCCCAGCTTTCCTTCGCAACTCG
>Bos_grunniens
GGTGGCCAAGTACCGTTTCGACCTCCAATTCCTAACGTGGGACTTCTCCTGAGACGCTGT
AGCGGGAAAGGGCTTCATCTTGCGATGACGGGGGAACCACGTGGTTTTTCTCGAGTTGCG
GCGGGATTCTCGAGTTACGACGGGGAATTCAGGATGCCTCTTGTGTTGGCCCAGGGAAGT
CCAATCTTCCATTCGAGTTGCGAAGGAAAGCTGGGGATTGCTCTCGAGTGACTGCAGGGC
CAATAGACATCATCTAGGCTTGTGTCCAGAAGCCAATGTTCCTCTCCAGGGCCGACAGGG
ATCTCGGGTTGCATTCCAGACGCACCCGGGGAGACAGGCATTCATTCTCGAGTGGAAGCA
AAGAACCCCCGCCTGCTCTCGAATCGCGACGGGTATCTCTTGGAGCTCACTGGGTGGACT
AAAGGGAGTCAAGCCTCCTGAGGCGCTTGGAGAGAGGTCGCGAGATTTGGTCTCTAGGCC
ATGCAGGAGACGAAGGCCCCTCATCTCGCGATGACGGGGGAGTCTCGGGGTTGTTCTCGA
GCGGCGGCCCCAGGGTGCGGTTTCTCACGAGGTACGACGGCGAGGTCAGTGAGCCTCTCG
TGGGGCCCAGGGAAGTCGGGTCTCCATGCAAGTGGCGAGGGGGAGCGCGTCATTGCTCCT
CGGAGTCATGGTAGGGGAATCTGGCCTCGAGACGTGTTGAAGAAGGTCTCTCGAGGGGGT
CTTTCTCGGTTGAGGCAGGAAACACTGGTTCCCTCGACTTGTGCAGGTGACCTCAGGGGG
CTTCTCATGGTGGCTCTGAGAAGTCAGGGAAACTGGAGGTTGGGAGGGGCCTCTCGGGAC
TCCATGGGGTTGGTGCATTGGAAGAGGGCCTCATCTCCAGTTGAGGCAGGAACCTCAGGG
TTCCTCTGATTTCAGACCGATCGCAGGGTCCCTGCAGACTTGGGACAGGAGAGTCAGGCC
TCGTCTTGGGTTGAGGCATGGAACTCCGCTTGCCTCTCGAGGTGTCCCCGGGGAGAGAGG
CCCATTGTCGAGCTGTATTTGGAACCTGGGGGTTTTCCCCGAACGATGCACGGGCGAGTC
ACTGCCCCTTCGTGTTGACTTCATTCACAGGGTGGAGTTCGGAAGAGGTGTCCGGGCATC
GGGTTCTTATCAAGAGGGGACCGGGAAATCGGGTGTCTTACGCAATGTGGAACCACCCAC
GAGGCCACGTCCTGGAATGTCGTCGTGAGACCGGCCTCATCCTGAGGTGCGACCGGAAGG
TCGGGAACCCCTTCCAGAAAAAGCAGGGGAGTCGACCCTCCTGTCCAGATCAGGAGGGTA
GAAGGGGCTCAGAGGAAGTGGTGGCCGGAAAACCTCAGGGGTTCCTCTCCGAGGGAGACC
GGGATGTCGGGAACTTTGTGGGTCGCATCAAG

References for Supplementary Table S2

(Wade et al., 2009; Hasson et al., 2011; Alkan et al., 2011)
(Matzke et al., 1990; Shang et al., 2010)
(Tek et al., 2010)
(Lee et al., 1997; Alkan et al., 2007; Hasson et al., 2011)
(Alkan et al., 2011)
(Alkan et al., 2011)
(Haaf et al., 1995; Alkan et al., 2007; Locke et al., 2011)
(Zhong et al., 2002; Nagaki et al., 2004)
(Cheng et al., 2002; Lee et al., 2005)
(Heslop-Harrison et al., 1999; Hall et al., 2003; Zhang et al., 2008)

(Krzywinski et al., 2005)
(Tarès et al., 1993; Beye and Moritz, 1995)
(Cellamare et al., 2009)
(Alkan et al., 2011)
(Abad et al., 1992; Abad et al., 2000; Sun et al., 2003)
(Haaf et al., 1995; Samonte et al., 1997; Alkan et al., 2007)
(Musich et al., 1980; Alkan et al., 2007)
(Fishman and Saunders, 2008)
(Guenatri et al., 2004; Kuznetsova et al., 2006)
(Cellamare et al., 2009)
(Alkan et al., 2011)
(Haaf et al., 1995; Samonte et al., 1997; Alkan et al., 2007)
(Musich et al., 1980; Alkan et al., 2007)
(Viñas et al., 2004)
(Tek and Jiang, 2004)
(Jiang et al., 1996; Zwick et al., 2000)
(Fischer et al., 2000; Roest Crollius et al., 2000)
(Ugarković et al., 1996)
(Harrison and Heslop-Harrison, 1995; Lim et al., 2007; Koo et al., 2011)
(Teschke et al., 1991; Niedermaier and Moritz, 2000)
(Alkan et al., 2011)
(Meyer et al., 2010)
(Ekes et al., 2004)
(Kikuchi et al., 2011)
(Tanaka et al., 1999; Pathak et al., 2006)
(Kipling et al., 1995)
(Spence et al., 1998)
(Jantsch et al., 1990; Bratanich et al., 2000)
(Gaillard et al., 1981; Płucienniczak et al., 1982; Taparowsky and Gerbi, 1982)
(Sternes and Vig, 1995)
(Buckland, 1983; Novak, 1984)
(Argout et al., 2011)
(Lee et al., 2011)
(Carone et al., 2009)
(Samonte et al., 1997; Alkan et al., 2007)

Supplementary Figures.

Figure S1 - group alignment (from Figure 2 - The Tree):
For each of the 26 groups of species with sequence similarity as depicted by the red bars in Figure 2, the alignment is shown in top-to-bottom order.

Figure S2 - primates and cichlids:
The colored bars represent the lenght of the centromere repeat sequences and the colors represent sequence similarity.
A There were 21 primates in the 282 species. All the Old World Monkeys (macaques and baboons) and apes (gibbons, gorillas, chimpanzees, bonobos, oerangutans, and humans) have similar 171 bp centromere repeat sequences. The New World Monkeys (spidermonkey, marmoset, squirrelmonkey) have a 343 bp repeat. This 343 bp repeat is a

doubled version of the 171 bp repeat (ligher blue bars, Figure 5D). The (candidate) centromere repeat sequences of the more basal tarsiers and prosimians did not show sequence similarity with the apes or monkeys or between themselves.
B The centromere repeat sequences of the Lake Malawi cichlids (*Metriclima zebra, Melanochromis auratus, Labeotrophus fuelleborni, Rhamphochromis esox,* and *Pundamilia nyererei*) and the nile tilapia (*Oreochromis niloticus*) showed sequence similarity, eventhough the nile tilapia centromere repeat is 20 bp shorter than the 237 bp repeat found in the Lake Malawi cichlids. Surprisingly, the centromere repeat sequence of the Lake Tanganyika cichlid (*Neopamprologus brichardi*) did not show sequence similarity with either the Lake Malawi cichlids or the nile tilapia.

Figure S3 - correlations between karyotype, genome size and our found parameters (length, GC content, genomic fraction):
Genome size and karyotype were correlated to tandem repeat length, GC content and genomic fraction, but no correlation was observed.

Figure S4 - Nile tilapia (*Oreochromis niloticus*) tandem repeat alignment:
In Nile tilapai (*Oreochromis niloticus*) is a close relative to the cichlids and the centromere repeat sequence is similar to the candidate centromere repeat sequence in Malawi cichlids, except that the most abundant tandem repeat in Nile tilapia has a 206 rather than a 237 bp repeat. In addition, a 237 bp repeat is also present in the Nile tilapia genome, but is less abundant. The alignment shows a 20 bp indel.

Figure S5 - Bovideae fractions of short (680 bp) and long repeat (1410 bp):
In Hereford cattle (*Bos taurus taurus*), Nellore cattle (*Bos taurus indicus*), yak (*Bos grunniens*), bison (*Bison bison*), and waterbuffalo (*Bubalus bubalis*) two predominant tandem repeats were found. One was around ~680 bp in length (named short) and one was around 1410 bp (named long). No sequence similarity was found between these two repeats, but all five species had these repeats, albeit in different ratios. In waterbuffalo the short repeat was the most abundant, whereas in the other species the long repeat was the most abundant.

Table S1 - all species info:
For each of the 282 species the basic phylogenetic, repeat, and genome information is given as well as the accession numbers used. The accession numbers for the NCBI Trace Archive (Sanger data) and DDBJ DRA (Illumina and 454 data) is given seperatly.

Table S2 - literature comparison:
For 60 of the 282 species literature data was available. For 51 of the 60 species we found the published repeat.

Table S3 - PRICE vs Sanger discordance explanation:
For 10 of the 37 we found a different repeat between PRICE assembled contigs and tandem repeat directly derived from Sanger sequences. Each case is individually addressed.

Table S4 - Number of species with data from a particular sequencing technology.

References

Abad JP, Agudo M, Molina I, Losada A, Ripoll P, Villasante A. 2000. Pericentromeric regions containing 1.688 satellite DNA sequences show anti-kinetochore antibody staining in prometaphase chromosomes of Drosophila melanogaster. *Mol Gen Genet* **264:**371-377.

Abad JP, Carmena M, Baars S, Saunders RD, Glover DM, Ludeña P, Sentis C, Tyler-Smith C, Villasante A. 1992. Dodeca satellite: a conserved G+C-rich satellite from the centromeric heterochromatin of Drosophila melanogaster. *Proc Natl Acad Sci U S A* **89:**4663-4667.

Alkan C, Cardone MF, Catacchio CR, Antonacci F, O'Brien SJ, Ryder OA, Purgato S, Zoli M, Della Valle G, Eichler EE, *et al.* 2011. Genome-wide characterization of centromeric satellites from multiple mammalian genomes. *Genome Res* **21:**137-145.

Alkan C, Ventura M, Archidiacono N, Rocchi M, Sahinalp SC, Eichler EE. 2007. Organization and evolution of primate centromeric DNA from whole-genome shotgun sequence data. *PLoS Comput Biol* **3:**1807-1818.

Argout X, Salse J, Aury JM, Guiltinan MJ, Droc G, Gouzy J, Allegre M, Chaparro C, Legavre T, Maximova SN, *et al.* 2011. The genome of Theobroma cacao. *Nat Genet* **43:**101-108.

Benson G. 1999. Tandem repeats finder: a program to analyze DNA sequences. *Nucleic Acids Res* **27:**573-580.

Beye M, Moritz RF. 1995. Characterization of honeybee (Apis mellifera L.) chromosomes using repetitive DNA probes and fluorescence in situ hybridization. *J Hered* **86:**145-150.

Bratanich AC, Ellis JA, Blanchetot A. 2000. Representational differential analysis detects amplification of satellite sequences in postweaning multisystemic wasting syndrome of pigs. *J Vet Diagn Invest* **12:**328-331.

Buckland RA. 1983. Comparative structure and evolution of goat and sheep satellite I DNAs. *Nucleic Acids Res* **11:**1349-1360.

Carone DM, Longo MS, Ferreri GC, Hall L, Harris M, Shook N, Bulazel KV, Carone BR, Obergfell C, O'Neill MJ, *et al.* 2009. A new class of retroviral and satellite encoded small RNAs emanates from mammalian centromeres. *Chromosoma* **118:**113-125.

Cellamare A, Catacchio CR, Alkan C, Giannuzzi G, Antonacci F, Cardone MF, Della Valle G, Malig M, Rocchi M, Eichler EE, *et al.* 2009. New insights into centromere organization and evolution from the white-cheeked gibbon and marmoset. *Mol Biol Evol* **26:**1889-1900.

Chen D-, Ronald PC. 1999. A Rapid DNA Minipreparation Method Suitable for AFLP and Other PCR Applications. *Plant Molecular Biology Reporter* **17:**53-57.

Cheng Z, Dong F, Langdon T, Ouyang S, Buell CR, Gu M, Blattner FR, Jiang J. 2002. Functional rice centromeres are marked by a satellite repeat and a centromere-specific retrotransposon. *Plant Cell* **14:**1691-1704.

Ekes C, Csonka E, Hadlaczky G, Cserpán I. 2004. Isolation, cloning and characterization of two major satellite DNA families of rabbit (Oryctolagus cuniculus). *Gene* **343:**271-279.

Fischer C, Ozouf-Costaz C, Roest Crollius H, Dasilva C, Jaillon O, Bouneau L, Bonillo C, Weissenbach J, Bernot A. 2000. Karyotype and chromosome location of characteristic tandem repeats in the pufferfish Tetraodon nigroviridis. *Cytogenet Cell Genet* **88:**50-55.

Fishman L, Saunders A. 2008. Centromere-associated female meiotic drive entails male fitness costs in monkeyflowers. *Science* **322:**1559-1562.

Gaillard C, Doly J, Cortadas J, Bernardi G. 1981. The primary structure of bovine satellite 1.715. *Nucleic Acids Res* **9:**6069-6082.

Guenatri M, Bailly D, Maison C, Almouzni G. 2004. Mouse centric and pericentric satellite repeats form distinct functional heterochromatin. *J Cell Biol* **166:**493-505.

Haaf T, Mater AG, Wienberg J, Ward DC. 1995. Presence and abundance of CENP-B box sequences in great ape subsets of primate-specific alpha-satellite DNA. *J Mol Evol* **41:**487-491.

Hall SE, Kettler G, Preuss D. 2003. Centromere satellites from Arabidopsis populations: maintenance of conserved and variable domains. *Genome Res* **13:**195-205.

Harrison GE, Heslop-Harrison JS. 1995. *Centromeric repetitive DNA sequences in the genus .*

Hasson D, Alonso A, Cheung F, Tepperberg JH, Papenhausen PR, Engelen JJ, Warburton PE. 2011. Formation of novel CENP-A domains on tandem repetitive DNA and across chromosome breakpoints on human chromosome 8q21 neocentromeres. *Chromosoma* **120:**621-632.

Heslop-Harrison JS, Murata M, Ogura Y, Schwarzacher T, Motoyoshi F. 1999. Polymorphisms and genomic organization of repetitive DNA from centromeric regions of Arabidopsis chromosomes. *Plant Cell* **11:**31-42.

Jantsch M, Hamilton B, Mayr B, Schweizer D. 1990. Meiotic chromosome behaviour reflects levels of sequence divergence in Sus scrofa domestica satellite DNA. *Chromosoma* **99:**330-335.

Jenkins G, Hasterok R. 2007. BAC 'landing' on chromosomes of Brachypodium distachyon for comparative genome alignment. *Nat Protoc* **2:**88-98.

Jiang J, Nasuda S, Dong F, Scherrer CW, Woo SS, Wing RA, Gill BS, Ward DC. 1996. A conserved repetitive DNA element located in the centromeres of cereal chromosomes. *Proc Natl Acad Sci U S A* **93:**14210-14213.

Kikuchi S, Tsujimoto H, Sassa H, Koba T. 2011. JcSat1, a novel subtelomeric repeat of Jatropha curcas L. and its use in karyotyping. *Chromosome Science* **13:**11-16.

Kipling D, Mitchell AR, Masumoto H, Wilson HE, Nicol L, Cooke HJ. 1995. CENP-B binds a novel centromeric sequence in the Asian mouse Mus caroli. *Mol Cell Biol* **15:**4009-4020.

Koo DH, Hong CP, Batley J, Chung YS, Edwards D, Bang JW, Hur Y, Lim YP. 2011. Rapid divergence of repetitive DNAs in Brassica relatives. *Genomics* **97:**173-185.

Krzywinski J, Sangaré D, Besansky NJ. 2005. Satellite DNA from the Y chromosome of the malaria vector Anopheles gambiae. *Genetics* **169:**185-196.

Kuznetsova I, Podgornaya O, Ferguson-Smith MA. 2006. High-resolution organization of mouse centromeric and pericentromeric DNA. *Cytogenet Genome Res* **112:**248-255.

Lee C, Wevrick R, Fisher RB, Ferguson-Smith MA, Lin CC. 1997. Human centromeric DNAs. *Hum Genet* **100:**291-304.

Lee HR, Hayden KE, Willard HF. 2011. Organization and molecular evolution of CENP-A--associated satellite DNA families in a basal primate genome. *Genome Biol Evol* **3:**1136-1149.

Lee HR, Zhang W, Langdon T, Jin W, Yan H, Cheng Z, Jiang J. 2005. Chromatin immunoprecipitation cloning reveals rapid evolutionary patterns of centromeric DNA in Oryza species. *Proc Natl Acad Sci U S A* **102:**11793-11798.

Lim KB, Yang TJ, Hwang YJ, Kim JS, Park JY, Kwon SJ, Kim J, Choi BS, Lim MH, Jin M, *et al.* 2007. Characterization of the centromere and peri-centromere retrotransposons in Brassica rapa and their distribution in related Brassica species. *Plant J* **49:**173-183.

Locke DP, Hillier LW, Warren WC, Worley KC, Nazareth LV, Muzny DM, Yang SP, Wang Z, Chinwalla AT, Minx P, *et al.* 2011. Comparative and demographic analysis of orang-utan genomes. *Nature* **469:**529-533.

Matzke MA, Varga F, Berger H, Schernthaner J, Schweizer D, Mayr B, Matzke AJ. 1990. A 41-42 bp tandemly repeated sequence isolated from nuclear envelopes of chicken erythrocytes is located predominantly on microchromosomes. *Chromosoma* **99:**131-137.

Meyer JM, Kurtti TJ, Van Zee JP, Hill CA. 2010. Genome organization of major tandem repeats in the hard tick, Ixodes scapularis. *Chromosome Res* **18:**357-370.

Musich PR, Brown FL, Maio JJ. 1980. Highly repetitive component alpha and related alphoid DNAs in man and monkeys. *Chromosoma* **80:**331-348.

Nagaki K, Cheng Z, Ouyang S, Talbert PB, Kim M, Jones KM, Henikoff S, Buell CR, Jiang J. 2004. Sequencing of a rice centromere uncovers active genes. *Nat Genet* **36:**138-145.

Niedermaier J, Moritz KB. 2000. Organization and dynamics of satellite and telomere DNAs in Ascaris: implications for formation and programmed breakdown of compound chromosomes. *Chromosoma* **109:**439-452.

Novak U. 1984. Structure and properties of a highly repetitive DNA sequence in sheep. *Nucleic Acids Res* **12:**2343-2350.

Pathak D, Srivastava J, Premi S, Tiwari M, Garg LC, Kumar S, Ali S. 2006. Chromosomal localization, copy number assessment, and transcriptional status of BamHI repeat fractions in water buffalo Bubalus bubalis. *DNA Cell Biol* **25:**206-214.

Płucienniczak A, Skowroński J, Jaworski J. 1982. Nucleotide sequence of bovine 1.715 satellite DNA and its relation to other bovine satellite sequences. *J Mol Biol* **158:**293-304.

Roest Crollius H, Jaillon O, Dasilva C, Ozouf-Costaz C, Fizames C, Fischer C, Bouneau L, Billault A, Quetier F, Saurin W, *et al.* 2000. Characterization and repeat analysis of the compact genome of the freshwater pufferfish Tetraodon nigroviridis. *Genome Res* **10:**939-949.

Samonte RV, Ramesh KH, Verma RS. 1997. Comparative mapping of human alphoid satellite DNA repeat sequences in the great apes. *Genetica* **101:**97-104.

Shang WH, Hori T, Toyoda A, Kato J, Popendorf K, Sakakibara Y, Fujiyama A, Fukagawa T. 2010. Chickens possess centromeres with both extended tandem repeats and short non-tandem-repetitive sequences. *Genome Res* **20:**1219-1228.

Spence JM, Blackman RL, Testa JM, Ready PD. 1998. A 169-base pair tandem repeat DNA marker for subtelomeric heterochromatin and chromosomal rearrangements in aphids of the Myzus persicae group. *Chromosome Res* **6:**167-175.

Sternes KL, Vig BK. 1995. Satellite I DNA in transformed rat cells. *Cancer Genet Cytogenet* **79:**64-69.

Sun X, Le HD, Wahlstrom JM, Karpen GH. 2003. Sequence analysis of a functional Drosophila centromere. *Genome Res* **13:**182-194.

Tanaka K, Matsuda Y, Masangkay JS, Solis CD, Anunciado RV, Namikawa T. 1999. Characterization and chromosomal distribution of satellite DNA sequences of the water buffalo (Bubalus bubalis). *J Hered* **90:**418-422.

Taparowsky EJ, Gerbi SA. 1982. Structure of 1.71 lb gm/cm(3) bovine satellite DNA: evolutionary relationship to satellite I. *Nucleic Acids Res* **10:**5503-5515.

Tarès S, Cornuet JM, Abad P. 1993. Characterization of an unusually conserved AluI highly reiterated DNA sequence family from the honeybee, Apis mellifera. *Genetics* **134:**1195-1204.

Tek AL, Jiang J. 2004. The centromeric regions of potato chromosomes contain megabase-sized tandem arrays of telomere-similar sequence. *Chromosoma* **113:**77-83.

Tek AL, Kashihara K, Murata M, Nagaki K. 2010. Functional centromeres in soybean include two distinct tandem repeats and a retrotransposon. *Chromosome Res* **18:**337-347.

Teschke C, Solleder G, Moritz KB. 1991. The highly variable pentameric repeats of the AT-rich germline limited DNA in Parascaris univalens are the telomeric repeats of somatic chromosomes. *Nucleic Acids Res* **19:**2677-2684.

Ugarković D, Podnar M, Plohl M. 1996. Satellite DNA of the red flour beetle Tribolium castaneum--comparative study of satellites from the genus Tribolium. *Mol Biol Evol* **13:**1059-1066.

Viñas A, Abuín M, Pardo BG, Martínez P, Sánchez L. 2004. Characterization of a new HpaI centromeric satellite DNA in Salmo salar. *Genetica* **121:**81-87.

Wade CM, Giulotto E, Sigurdsson S, Zoli M, Gnerre S, Imsland F, Lear TL, Adelson DL, Bailey E, Bellone RR, *et al.* 2009. Genome sequence, comparative analysis, and population genetics of the domestic horse. *Science* **326:**865-867.

Young HA, Hernlem BJ, Anderton AL, Lanzatella CL, Tobias CM. 2010. Dihaploid stocks of switchgrass isolated by a screening approach. *BioEnergy Research* **3:**305-313.

Young HA, Lanzatella CL, Sarath G, Tobias CM. 2011. Chloroplast genome variation in upland and lowland switchgrass. *PLoS One* **6:**e23980.

Zhang, P., and Friebe, B. 2009. *FISH on Plant Chromosomes. In Fluorescence In Situ Hybdrization (FISH) - Application Guide, Liehr T, ed (pp. 365 - 394)*. Springer Berlin Heidelberg.

Zhang W, Friebe B, Gill BS, Jiang J. 2010. Centromere inactivation and epigenetic modifications of a plant chromosome with three functional centromeres. *Chromosoma* **119:**553-563.

Zhang W, Lee HR, Koo DH, Jiang J. 2008. Epigenetic modification of centromeric chromatin: hypomethylation of DNA sequences in the CENH3-associated chromatin in Arabidopsis thaliana and maize. *Plant Cell* **20:**25-34.

Zhong CX, Marshall JB, Topp C, Mroczek R, Kato A, Nagaki K, Birchler JA, Jiang J, Dawe RK. 2002. Centromeric retroelements and satellites interact with maize kinetochore protein CENH3. *Plant Cell* **14:**2825-2836.

Zwick MS, Islam-Faridi MN, Zhang HB, Hodnett GL, Gomez MI, Kim JS, Price HJ, Stelly DM. 2000. Distribution and sequence analysis of the centromere-associated repetitive element CEN38 of Sorghum bicolor (Poaceae). *Am J Bot* **87:**1757-1764.
